# Early radiology reports and ECG machine measurements do not measurably improve 30-day ICU mortality prediction from the first 6 h of the stay: a multimodal ablation with external validation of the structured EHR backbone

**DOI:** 10.3389/fmedt.2026.1902843

**Published:** 2026-10-28

**Authors:** Aigerim Ismukhamedova, Anna Bakanova, Saule Belginova, Arkadiusz Gola, Aisat Khalimov

**Affiliations:** 1 School of Digital Technology and Artificial Intelligence, D. Serikbayev East Kazakhstan Technical University, Oskemen, Kazakhstan; 2 Department of Business, Kazakh-American Free University, Oskemen, Kazakhstan; 3 Higher School of Information Technology, Turan University, Almaty, Kazakhstan; 4 Department of Production Computerisation and Robotisation, Politechnika Lubelska, Lublin, Poland

**Keywords:** clinical notes, electrocardiogram, electronic health records, external validation, ICU mortality, modality fusion, multimodal learning

## Abstract

Whether electrocardiographic measurements and unstructured clinical notes add prognostic information to structured electronic health records for early ICU mortality prediction remains unresolved. We trained a modality-attention model on tabular EHR features, machine-derived ECG measurements, printed diagnostic statements, and biomedical language-model representations of notes, all drawn from the first 6 h after ICU admission. Within that window, the only usable text in MIMIC-IV is the radiology report, available for 19.6% of stays. The discharge summary is the only other free-text table, but in 98.6% of stays in which it was charted during the window, it was filed after the window had closed. Mortality was assessed at 48 h, 7 days, and 30 days after ICU admission, with 30 days as the primary endpoint. The 6-h window is the period from which the predictors are drawn, not the interval over which death is predicted. Post-window features, including total ICU length of stay, were excluded. The cohort comprised each adult's first ICU stay in MIMIC-IV v3.1 (65,366 stays), holding out 9,806 (30-day mortality 10.6%), without a minimum stay to avoid day-one survival conditioning. Five variants used five seeds per horizon. EHR-only AUROCs were 0.9371, 0.9106, and 0.8969. Adding ECG, text, or both produced no reliable improvement: at 30 days, every paired AUPRC difference included zero, and the largest gain compatible with the data, 0.0068, was of the order of the study's own training noise. These are confidence limits, not equivalence bounds. No defect was identified in the preprocessing checks performed on the ECG matrix, and the matrix’s highest-ranked input still ranked 106th of 1,827. L2-penalized logistic regression reached 0.8844, and the matched architecture added 0.0126 AUROC (0.0083–0.0168). All learned models outperformed SOFA at 0.731 and SAPS-II at 0.793, with DeLong *p* below 10^⁻⁵⁹^. Transferred without retraining, the models reached AUROC 0.833 on HiRID, 0.774 on MC-MED, and 0.7268 on eICU-CRD; none of these cohorts supplied ECG or notes, so they tested the structured backbone with both extra modalities masked, and the HiRID and MC-MED 30-day outcomes are derived from length of stay. Mean calibration gaps concealed over-prediction up to 0.167 in the highest-risk decile. AUROC was 0.853 for individuals aged 80 or above; race-stratified AUROC remained 0.878–0.914 compared with 0.897 overall. These findings pertain to early radiology reports and ECG summaries within a 6-h window.

## 1 Introduction

Identifying ICU patients at high risk of in-hospital death during the first hours after admission can support triage, monitoring intensity, and family communication. Most operational risk scores in critical care, including SOFA (1), SAPS-II (2), and APACHE-II, rely on a small set of structured physiologic variables and were developed for cohort description rather than individual prediction. Machine-learning models trained on the extensive MIMIC family of databases (3–7), using algorithms such as XGBoost (8) and LightGBM (9), have repeatedly demonstrated superior discrimination compared with these scores. However, most reported pipelines remain restricted to structured EHR data.

A natural next step is to investigate whether additional, lower-cost modalities (such as 12-lead ECG machine measurements and free-text clinical notes) provide complementary information that enhances discrimination, calibration, or robustness beyond what a strong EHR-only model captures. This question is not purely methodological: ECG and notes are routinely available, but pipelines that integrate them are seldom validated under realistic conditions of modality availability, domain shift, and demographic heterogeneity. Multimodal models are widely expected to outperform single-modality models (10), and frameworks that combine tabular, imaging, and free-text inputs on the MIMIC family report gains from fusion (11). Prior multimodal work has mostly focused on a single internal benchmark and has rarely separated the contribution of each modality from the contribution of the fusion architecture (6, 7, 12, 13). The gap addressed here is therefore narrower and more specific: Whether ECG machine measurements and early clinical text provide additional information to a strong structured EHR model when every predictor is limited to what is available in the first 6 h of the ICU stay, when the contribution of each modality is separated from that of the fusion architecture, and when the resulting models are tested outside the development institution.

Our previous work on machine-learning-based medical decision-support systems and healthcare data integration informed the methodological design of the present study (14–16). In particular, these studies highlighted the importance of careful feature engineering, model interpretability, and the integration of heterogeneous clinical data sources in healthcare decision-support applications.

We therefore designed a controlled experiment built around five model variants that share the same EHR backbone and the same evaluation cohorts: A (EHR-only), B (EHR+ECG), C (EHR+text), D (EHR+ECG+text), and D_robust (D with modality dropout during training). By varying only the set of input modalities and keeping the fusion architecture, training objective, and hyperparameter search fixed across variants, we quantify the marginal value of each modality. We then test transportability on three external cohorts: a multicenter US database (eICU-CRD), an academic US emergency-to-ICU cohort (MC-MED), and a European tertiary ICU (HiRID, Bern).

This work makes four contributions. First, it isolates the incremental value of ECG machine measurements and clinical notes over structured EHR within a single, controlled multimodal pipeline, finding it indistinguishable from zero, which is the paper’s result rather than a limitation. Second, it benchmarks the proposed models against SOFA and SAPS-II on the same cohort using paired DeLong testing. Third, it evaluates transfer to three independent ICU databases without retraining. None of these databases supplies ECG measurements or clinical notes at inference, so the evaluation concerns the structured EHR backbone with both extra modalities masked rather than the multimodal model. Fourth, it integrates SHAP-based interpretability and a demographic fairness audit to characterize where the model’s predictive value is most and least reliable.

## 2 Materials and methods

### 2.1 Study design and evaluation framework

This study was based on a development and external validation design intended to assess early ICU mortality prediction across several critical care data environments. The task is admission-window risk scoring: Every predictor is drawn from the first 6 h after ICU admission, and the cohort includes patients who die within that window, so the analysis is not prediction among 6-h survivors. A landmark analysis restricted to survivors is reported as a sensitivity analysis in Section 3.1 in two forms: A re-scoring of the models fitted on the full cohort and a retraining, at the 30-day and 48-h horizons, of every model on a cohort reconstructed at the landmark. The publicly accessible MIMIC-IV v3.1 database (3) was used as the development cohort, while three independent datasets, eICU-CRD v2.0 (17), MC-MED (18), and HiRID v1.1.1 (19, 20), were used for external validation. Across all cohorts, the analysis was restricted to adult patients and the first ICU admission of each patient to ensure independent observations. The adult criterion did not remove any admissions from MIMIC-IV, since its ICU module includes only adult patients, but it was retained to maintain consistency across datasets. The restriction to first ICU admissions reduced the MIMIC-IV cohort from 94,458 ICU stays to 65,366 unique patients.

No minimum ICU length-of-stay criterion was applied. Excluding short admissions would have removed patients who died soon after ICU admission and would have introduced survivorship bias. This decision was especially important because such patients represent a clinically relevant target group for early risk prediction. For this reason, all predictors were derived exclusively from information collected during the first 6 h after ICU admission, regardless of the total duration of the ICU stay. The 6-h window was chosen to provide early predictions that could inform triage, monitoring intensity, and early escalation decisions, which are made during the initial hours of the stay and before the 24 h of data required by SOFA and SAPS-II have accumulated. A longer window would contain more information but would deliver predictions after many of these decisions have already been made, and its predictors would increasingly reflect treatment that has already been administered. The consequences of this choice for the text modality are described in Section 2.2 and in the Limitations.

The MIMIC-IV cohort was randomly partitioned at the patient level into training, validation, and held-out test sets using a 70/15/15 split. This approach prevented information leakage between subsets and preserved the independence of model evaluation. The final development subsets included 45,756 patients for training, 9,804 for validation, and 9,806 for held-out testing. The 30-day mortality rate in the test set was 10.6%. The same inclusion criteria were applied to the external datasets. In eICU-CRD, the cohort decreased from 200,859 admissions to 138,868 first ICU admissions. In MC-MED, retaining only the first encounter reduced the sample from 2,766 to 2,575 patients. No additional filtering was required for HiRID because each patient identifier corresponded to a single admission. The resulting cohort characteristics are summarized in Table 1.

**Table 1:** Cohort characteristics across the four datasets.

Cohort	*N* (ICU stays)	Death within 48 h	Death within 7 days	Death within 30 days (primary)	In-hospital, any time	Median age	% Female	Country/setting
MIMIC-IV v3.1 (test split)	9,806	3.5%	6.9%	10.6%	11.0%	66	43.4%	US (Beth Israel Deaconess)
eICU-CRD v2.0	138,868	3.6%	6.8%	9.2%	—	—	—	US (208 hospitals)
MC-MED (ICU subset)	2,575	4.1%	6.8%	10.0%	—	—	—	US (Stanford Health Care)
HiRID v1.1.1	33,666	3.6%	5.4%	6.1%	—	—	—	Switzerland (Bern)

External event times come from hospitaldischargeoffset (eICU-CRD), hospital length of stay from ED arrival (MC-MED), and the last recorded observation of a patient who died (HiRID); the latter two are approximations, as neither database publishes a death timestamp.

The prediction target was in-hospital death within a fixed time window after ICU admission. Deaths occurred across a wide temporal range, from several hours after admission to approximately 8 months later. The median time to death was 4.4 days, with an interquartile range of 1.4–10.4 days and a 95th percentile of 28 days. A single in-hospital mortality endpoint would therefore combine events that may have substantially different relationships to the information available during the first 6 h of care. To address this issue, separate models were trained for prediction horizons of 48 h, 7 days, and 30 days. The 30-day endpoint was selected as the primary outcome because it captured 95.7% of all in-hospital deaths while remaining clinically relevant for early risk stratification. Patients discharged alive before the end of the prediction window and patients still hospitalized but alive at its conclusion were considered non-events. Since discharge and death times were available for every admission, outcome status was fully observed without censoring.

Model performance was evaluated using three complementary protocols. The first protocol assessed performance on subsets of the held-out test set defined by actual modality availability during the first 6 h after ICU admission. These subsets included 2,130 patients with ECG data, 1,904 patients with radiology reports, and 783 patients with both modalities. The second protocol evaluated performance on the complete held-out test set, representing the intended deployment scenario. In this setting, missing modalities were handled using modality-mask vectors, while unavailable inputs were replaced with learnable missing-modality embeddings in the fusion module. All primary results are reported under this complete-cohort protocol. The third protocol assessed transportability by applying the trained models directly to eICU-CRD, MC-MED, and HiRID without retraining and without access to external labels. This design evaluated external transfer under masked modalities, following the principle described by Steyerberg and Harrell (21). The overall evaluation framework is shown in Figure 1, and the complete cohort flow is presented in Figure 2.

**Figure 1:**
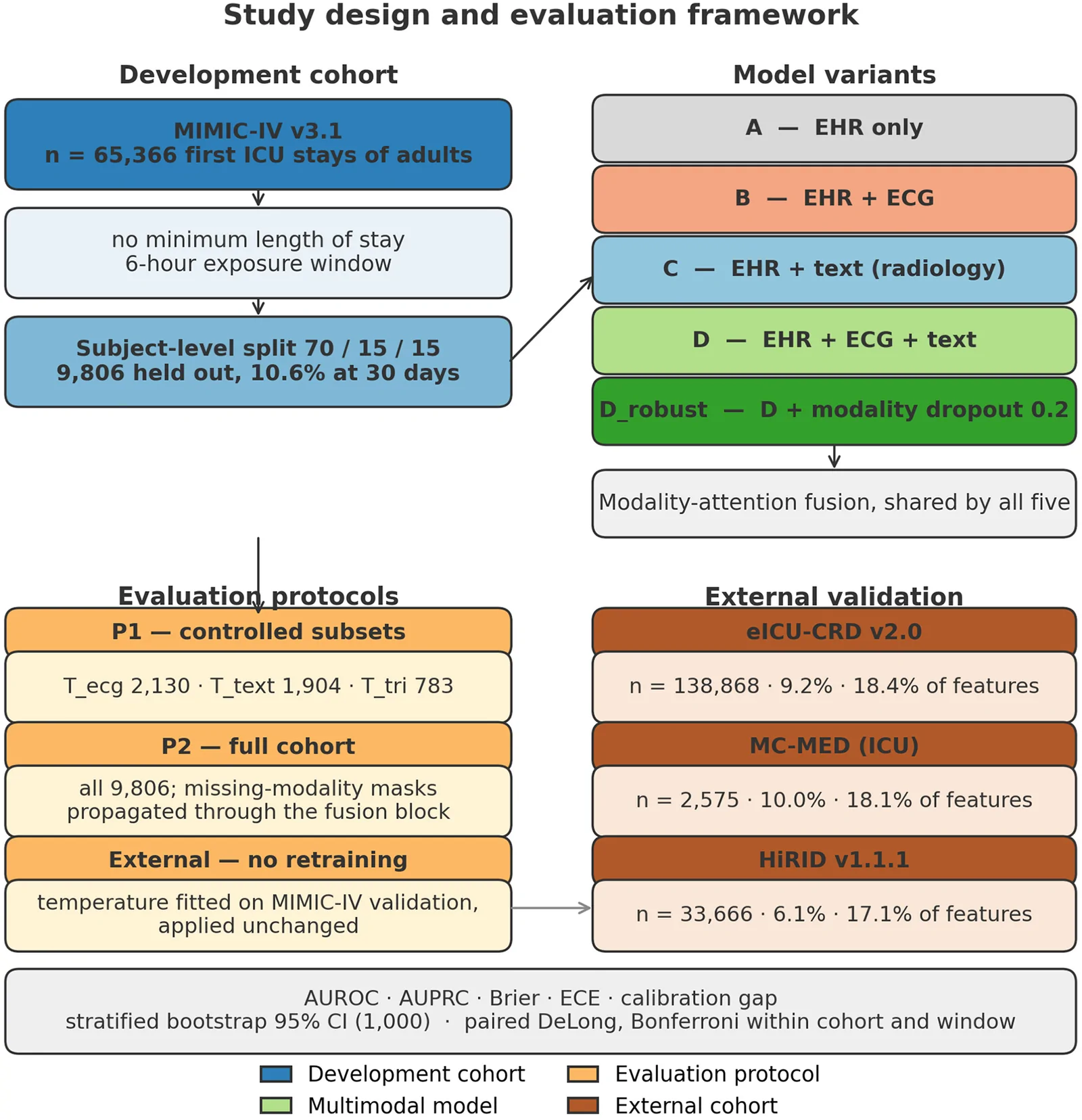
Study design and evaluation framework. The development cohort (MIMIC-IV v3.1) is split at the patient level into a 70/15/15 train/validation/test ratio; the three external cohorts (eICU-CRD v2.0, MC-MED, HiRID) are used without retraining. Five model variants (A: EHR-only, B: EHR + ECG, C: EHR + text, D: EHR + ECG + text, D_robust: D with training-time modality dropout) share the same fusion architecture. Three evaluation protocols are applied: P1 (real modality-availability subsets), P2 (full cohort with missing-modality masks), and external evaluation without retraining. None of the three external cohorts supplies ECG measurements or clinical notes, so the external arm evaluates the structured EHR backbone with both extra modalities masked.

**Figure 2:**
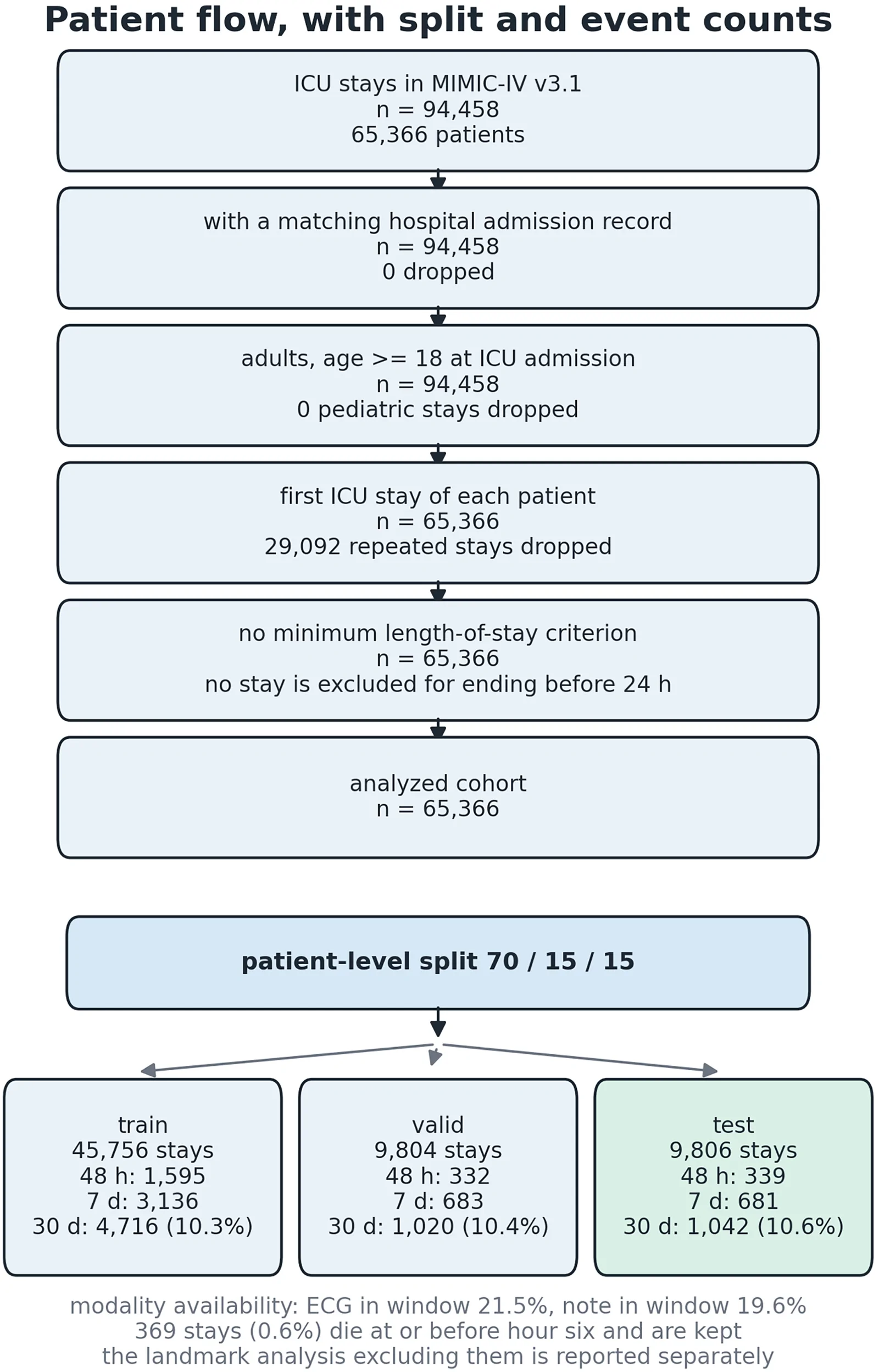
Patient flow. Exclusions are applied in the order that the cohort builder applies them (age, then one stay per patient), followed by the patient-level split with the event count at each of the three outcome windows. No minimum length-of-stay criterion is imposed. A total of 369 stays, or 0.6%, end in death at or before the sixth hour and are retained, while the landmark sensitivity analysis that excludes them is reported in Section 3.1.

### 2.2 Feature development and modeling

Structured EHR features were extracted from chartevents and labevents recorded during the first 6 h after ICU admission and supplemented with age and sex. To avoid information leakage, item selection was performed only within the training split. The 256 most frequent chart items and the 256 most frequent laboratory items were initially selected, and each item was summarized within the 6-h window using its mean, minimum, maximum, last value, and count. Items without numeric values in the window, mainly free-text chart entries, did not generate feature columns and were therefore excluded. This process retained 122 chart items and 195 laboratory items, producing 1,585 aggregated columns. After adding age and sex, the structured EHR modality contained 1,587 features.

All structured predictors were constrained to information available at the prediction time. This restriction was enforced not only by charting time but also by filing time. Observations with a storetime after the 6-h window were excluded even when their charttime fell within the window. This rule removed 11.0% of in-window chart events and 10.4% of laboratory events. A documented exception was made for 87,051 chart events and 13,331 laboratory events without a filing timestamp, corresponding to 0.33% and 0.48% of records because a missing timestamp was not treated as evidence of late filing. Total ICU length of stay was not used as a predictor because it is unknown at the time of early prediction. Missing numeric measurements were imputed with the training mean, which corresponds to zero after standardization. In contrast, per-item count features retained a true value of zero so that the absence of a measurement remained available to the model as an explicit signal. All numeric features were z-scored using training-split statistics and clipped at 10 standard deviations in both directions. The structured preprocessing procedure is summarized in Table 2.

**Table 2:** Preprocessing as the analysis pipeline performs it.

Step	Method	Parameters	Justification
Item selection	Top-*N* by event frequency in the training split	256 chart, 256 lab; items with no numeric value drop out, leaving 122 and 195	Bound the matrix without consulting the outcome
Within-window aggregation	Mean/min/max/last/count over 6 h	All retained EHR items	Short-term level, extremes, trend endpoint, and measurement frequency
Missing values	Training-mean imputation for value columns; count columns keep their true zero	Mean from the training split only	An unmeasured value is not an extreme value; an unmeasured *item* is an observation about the patient
Normalization	Z-score, then clipped	Mean/std fit on the training fold only; clip at ±10 SD	Required for gradient-based encoders; the clip bounds transfer artifacts
Outlier handling	The ±10 SD clip above; no IQR rule and no winsorization are applied	-	Stated explicitly
ECG statements	Multi-hot over the 100 most frequent normalized statements on the training split	100 statements; undetectable *P* wave kept as an explicit indicator	The statement channel is where the model's ECG attribution resides (Section 3.3)
Calibration (applied at evaluation time, not in the feature builder)	Single-parameter temperature scaling on the mean logit	One temperature per variant per outcome window, fitted on the MIMIC-IV validation split	Probability rescaling; monotone in the logit, so discrimination is unaffected

Every rule is fitted on the training split alone and applied unchanged to the validation and test splits, as well as to all three external cohorts.

For the ECG modality, 12-lead machine-derived measurements were obtained from the MIMIC-IV-ECG matched subset (22). ECG studies recorded within the 6-h window were averaged at the stay level and projected into a 128-dimensional embedding using a feed-forward encoder. The raw ECG table required additional preprocessing because it contained sentinel codes, fiducial timing fields, and machine-generated diagnostic statements. Sentinel values were used instead of nulls when measurements failed, including 29,999 when no *P* wave was detected and 32,767 or 65,535 for other failures. Fiducial columns represented onsets and offsets relative to an arbitrary reference and were interpretable only through derived differences. The table also included 18 columns containing diagnostic statements as free text, which would be lost under simple numeric coercion. To address these issues, sentinel and physiologically implausible values were set to missing. Clinically interpretable intervals were then derived, including heart rate, PR interval, *P* duration, QRS duration, QT interval, and QTc using Bazett's formula. The three frontal-plane axes were retained, including negative values.

Failure to detect a *P* wave was preserved as a binary indicator because it is clinically informative rather than merely a missing value. This pattern appeared in 25.8% of records. The absence of a detectable *P* wave is frequently associated with atrial fibrillation, although the indicator records the algorithm's failure to detect a *P* wave rather than a diagnosis. In addition, the 100 most frequent normalized diagnostic statements were multi-hot encoded using frequencies calculated in the training split. These statements covered 86.8% of all printed diagnostic statements in the cohort. The final ECG matrix contained 110 columns.

Two imputation rules were deliberately used within the ECG modality. Derived measurements that the machine could not produce were imputed with the training mean, while diagnostic statements that were not printed were encoded as zero. The same zero encoding was applied to all statement columns for stays without an ECG in the 6-h window. As a result, statement features also conveyed information about whether an ECG was ordered, an issue revisited in Section 3.3. The rebuilt ECG values were physiologically plausible. For heart rate, the median was 79 bpm and the interquartile range was 67–95 bpm. For PR interval, the median was 158 ms and the interquartile range was 140–182 ms. For QRS duration, the median was 94 ms and the interquartile range was 86–112 ms. For QT interval, the median was 400 ms and the interquartile range was 360–436 ms. For QTc, the median was 458 ms and the interquartile range was 436–484 ms. For the QRS axis, the median was 13° and the interquartile range was −19° to 50°. This study used only machine-derived ECG measurements and statements. Raw ECG waveforms were beyond its scope.

The text modality was defined to match the 6-h feature window. MIMIC-IV contains only two free-text tables: discharge summaries and radiology reports. Nursing notes, physician notes, and progress notes available in MIMIC-III have no direct counterpart in MIMIC-IV, and the resource does not include admission notes. Discharge summaries were excluded because they are written after the hospital course has ended and therefore cannot support early prediction. Across the 94,458 ICU stays in the source database, 1,211 stays had a discharge summary charted within the 6-h window. This represented 1.3% of stays, but 98.6% of those summaries had a filing timestamp after the end of the prediction window. Mortality in this subgroup was 34.85%, compared with 12.02% overall, indicating substantial outcome-related leakage. The text modality was therefore restricted to radiology reports whose examination and filing times both occurred no later than ICU admission time plus 6 h. These reports physically existed at the moment of prediction. Under this definition, text was available for 12,830 of 65,366 stays, corresponding to 19.6% of the cohort, and for 1,904 of 9,806 test stays. For each stay, up to eight reports were concatenated in chronological order. The resulting text was truncated to 256 word-piece tokens, encoded with a frozen biomedical transformer (23), and mean-pooled into a 128-dimensional embedding.

The three modality embeddings were passed to a modality-attention fusion block designed for partially observed modality sets. Attention weights were conditioned on the patient-level availability mask, ensuring that absent modalities received zero weight and available modalities were reweighted accordingly, following the logic of fusion architectures for incomplete multimodal data (24). In the implementation, an absent modality is excluded from both the attention keys and the pooling of the attended embeddings, so it contributes nothing to the fused representation; the learnable missing-modality embedding only fills its slot in the input stack. The three-element availability mask is then concatenated to the pooled representation before the classification head, making the availability of modalities explicitly reach the prediction. This is why the availability indicators appear as separate inputs in the attribution analysis (Section 3.3). A patient without ECG or text is therefore scored based on the structured EHR embedding and the availability mask alone. The fused 128-dimensional representation is then passed to a single classification head. The robustness variant, D_robust, used the same architecture but applied stochastic modality dropout during training at a rate of 0.2 for both the ECG and text channels. During each training step, the ECG and text channels of every patient in the batch were independently marked as absent with probability 0.2, in addition to their true absence, by setting the corresponding availability mask to zero. The purpose was to expose the fusion block during training to the missing-modality patterns it encounters at inference, both in the MIMIC-IV test set, where most patients have neither an ECG nor a radiology report within the window, and in the external cohorts, where both are absent, so that predictions would not depend on a modality being present. Whether this conferred any advantage is tested in Section 3.6. The five model variants and their modality inputs are summarized in Table 3.

**Table 3:** Model variants and modalities.

Variant	EHR	ECG (machine measurements)	Clinical notes	Training-time modality dropout
A	✓			
B	✓	✓		
C	✓		✓	
D	✓	✓	✓	
D_robust	✓	✓	✓	ECG 0.2, text 0.2

Each model variant was trained as an ensemble of five seeds, numbered 42–46. Training used AdamW with a learning rate of 3×10^⁻⁴^, weight decay of 10^⁻⁵^, a batch size of 24, and gradient accumulation over two steps. The selected epoch was determined by validation AUROC. Training was limited to 20 epochs with a patience of 5. For every trained model, the training loss, validation loss, and validation AUROC were recorded at each epoch. This produced records for 75 models in the main grid and for an additional 10-model sensitivity arm. The retrained landmark analysis of Section 3.1 adds 50 further models at the 30-day and 48-h horizons, trained under the same protocol. After training, each ensemble was calibrated on the validation set using single-parameter temperature scaling.

### 2.3 Baselines

Three baseline approaches were used to contextualize model performance. SOFA and SAPS-II were chosen because they are the severity scores most widely used in critical care and can be reconstructed from the same MIMIC-IV tables for every test patient, indicating what a learned model adds to current practice. APACHE IVa is not available in MIMIC-IV and was compared using eICU-CRD, which provides it (Section 3.8). The L2-penalized logistic regression was included for a different reason: It uses exactly the same inputs as the EHR-only neural model,therefore isolating the contribution of the model architecture. SOFA and SAPS-II were reconstructed from raw MIMIC-IV tables for every patient in the held-out test set. Discrimination was assessed from raw score values using AUROC, AUPRC, and DeLong testing. Because raw point scores are not probabilities, Brier scores were calculated from the published probability transformations of each score. SAPS-II used the transformation by Le Gall et al. (2), while SOFA used the logistic conversion based on Vincent et al. (1) and Lambden et al. (25). These transformations are monotonic, so they do not affect AUROC or AUPRC. Results are reported in Table 4.

**Table 4:** Benchmark against established clinical risk scores on the same MIMIC-IV held-out test set (*n* = 9,806).

Predictor	AUROC [95% CI]	AUPRC	Brier	ΔAUROC vs. model A
Model A	0.8969 [0.8878, 0.9062]	0.5809	0.0645	reference
Model B	0.8964 [0.8867, 0.9055]	0.5778	0.0647	−0.0005
Model C	0.8971 [0.8878, 0.9062]	0.5813	0.0643	+0.0002
Model D	0.8969 [0.8876, 0.9060]	0.5794	0.0645	+0.0000
Model D_robust	0.8972 [0.8883, 0.9065]	0.5778	0.0646	+0.0003
SOFA (raw)	0.7310 [0.7150, 0.7462]	0.2756	0.0929	−0.1659
SAPS-II (raw)	0.7934 [0.7789, 0.8063]	0.4044	0.1827	−0.1035
Logistic regression, 5 bagged fits	0.8843 [0.8749, 0.8945]	0.5436	0.0677	−0.0126 [−0.0168, −0.0083]
Logistic regression, single fit	0.8844 [0.8751, 0.8946]	0.5459	0.0675	(not fit-matched)

Scores are computed for the first 24 h of the stay as they prescribe, whereas the learned models consider only the first 6 h. The AUROC, AUPRC, and ΔAUROC columns use the raw score. The Brier column uses its published probability transformation (2) for SAPS-II; the Lambden/Vincent logistic conversion for SOFA (1, 25), since a Brier score is undefined on a raw scale. The transformations are monotonic, so the two conventions give identical discrimination (Section 2.4). The learned models are scored with the same ensemble probabilities used in Table 5, calibrated for Brier and uncalibrated for discrimination. Every AUROC interval in this table, including both logistic-regression rows, represents the 2.5th to 97.5th percentile of the stratified bootstrap of 1,000 resamples defined in Section 2.4. The interval beside the bagged fits is the interval of that model, while the paired difference against model A is given in the final column.

A third baseline used L2-penalized logistic regression fitted on the same 1,587 standardized structured EHR features and the same patient-level split as the EHR-only neural model. Penalty strength was selected based on validation AUROC, without *post-hoc* calibration. This baseline separated the contribution of neural architecture from that of the feature set. The fitted linear models were also applied without refitting to aligned external cohorts and evaluated against the same horizon-specific labels as the neural models.

### 2.4 Statistical analysis

Statistical procedures are reported in accordance with TRIPOD + AI (26). All metrics were computed on the held-out test set of the development cohort or on the external cohorts, never on data used for fitting or selection. A feature that was never measured was imputed with the training mean, and its count column retained a true zero, consistently during training and external inference. Section 3.4 shows how much a mismatch in that single rule can affect an external result.

Model performance was assessed using AUROC, AUPRC, Brier score, expected calibration error with ten equal-width bins, and calibration gap, defined as the mean predicted probability minus the observed event rate. Uncertainty was estimated using a stratified bootstrap over patients with 1,000 resamples drawn separately within event and non-event strata to preserve the event rate (27). The 2.5th and 97.5th percentiles were reported. Each patient contributed one ICU stay in the development cohort, so patient and observation were the same resampling unit, and no cluster-aware correction was required. External cohorts were resampled by their own admission unit: patient stays in HiRID and eICU-CRD and emergency-department visits in MC-MED, without assuming one stay per patient.

Predictions from the five ensemble seeds were averaged to obtain mean predicted probabilities. For calibration metrics, temperature scaling was applied using the sigmoid of the mean logit under a temperature fitted on the MIMIC-IV validation split. One temperature was fitted for each model variant and outcome window and then applied unchanged to every cohort. Across the 15 temperatures, values ranged from 1.0471 to 1.2284. Discrimination metrics were consistently computed from mean probabilities, while calibration metrics used temperature-scaled probabilities.

Pairwise AUROC differences between models evaluated on the same patients were tested using the paired DeLong test (28). A paired test was used because all variants score the same patients, meaning their AUROC estimates are correlated, and an unpaired comparison would overstate the uncertainty of their difference. AUPRC differences were summarized as paired bootstrap intervals on the same resamples rather than as *p*-values, since no equivalent analytic test was used. An interval excluding zero was described as nominal and was not treated as surviving correction. Bonferroni correction was applied across the 10 model pairs formed by the five variants, within each cohort and outcome window. This rule was used for both internal comparisons in Section 3.1 and external comparisons in Section 3.4. Comparisons across windows, cohorts, and demographic subgroups were not corrected because they addressed different questions and were reported as exploratory.

SHAP values (29) were computed with shap.GradientExplainer applied directly to the trained network rather than to a surrogate model. Explanations were generated separately for the EHR branch of model A and for the EHR and ECG branches of model D. Expected gradients were estimated using a class-stratified sample of 2,000 held-out patients, a background of 500 training patients, and 50 gradient samples per point. Two explainer setups were used. The first masked the other modalities and supported readable within-branch rankings. For model D, it also held the ECG availability mask at one for every patient, meaning the first setup is not the model that produced the predictions. Cross-modality statements therefore relied on a second setup, which attributed predictions over the concatenated EHR matrix, ECG matrix, text embedding, and two availability indicators using each patient's own notes and masks. That setup was checked against the evaluation pipeline before any attribution was used. Only that setup supported pooled rankings. The SHAP output for the EHR backbone of model A is shown in Figure 3, and the full fidelity procedure is provided in the Supplementary Methods.

**Figure 3:**
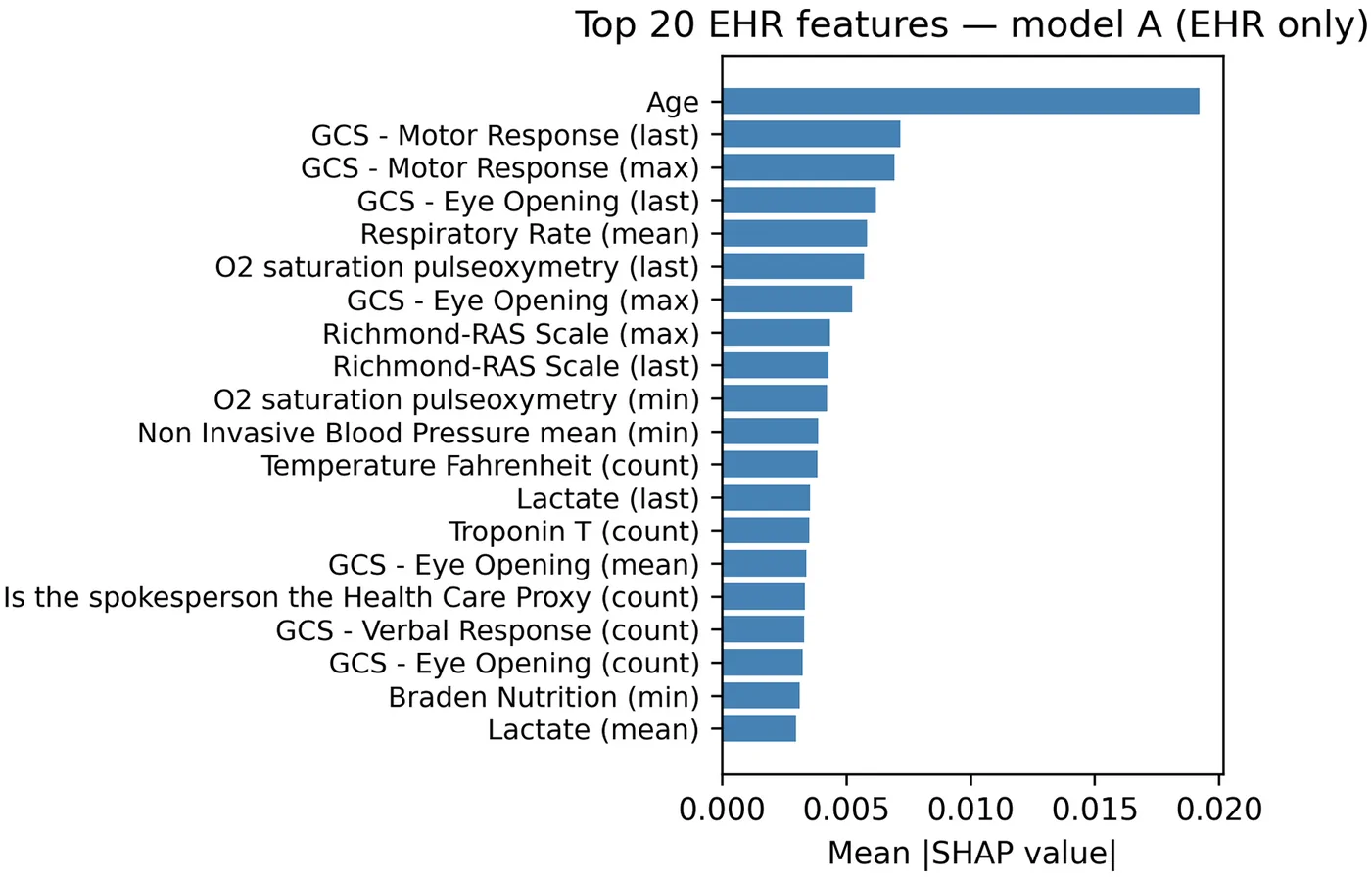
SHAP global feature-importance ranking for the EHR backbone of model A, computed with shap.gradientExplainer over a class-stratified sample of 2,000 of the 9,806 held-out patients, against a background of 500 training patients. The six leading features are Age, GCS—Motor Response (last), GCS—Motor Response (max), GCS—Eye Opening (last), Respiratory Rate (mean), and O2 saturation by pulse oximetry (last). Total ICU length of stay is not a feature, and heart rate ranks 23rd as a window maximum and 31st as a window mean. Supplementary Figure S1 shows the ECG branch of model D, where the machine statements “sinus rhythm,” “sinus tachycardia,” and “abnormal ECG” carry the largest attributions. Figure 6 presents model D's top 20 with both branches on one axis. No ECG input appears over the full held-out set; the highest (the printed statement “sinus rhythm”) ranks 106th, and the first derived measurement ranks 597th, though the statement ranks thirteenth among patients who have an ECG and second only under a background matched on ECG availability (Section 3.3).

## 3 Results

### 3.1 Internal performance and modality contribution on MIMIC-IV

Across the five model variants evaluated on the complete held-out test set for the primary 30-day outcome, performance was nearly identical. Under protocol P2, the test set included 9,806 patients and 1,042 events. AUROC values ranged only from 0.8964 to 0.8972, and the largest difference between any two variants was 0.0008, as shown in Table 5 and Figure 4. When each model was compared with the EHR-only baseline on the same patients, no added modality demonstrated a reliable AUPRC gain. Adding ECG machine measurements produced a paired ΔAUPRC of −0.0032, with a 95% confidence interval from −0.0081 to 0.0018. Adding prospectively available radiology text produced a ΔAUPRC of +0.0004, with an interval from −0.0036 to 0.0044. The tri-modal model produced a ΔAUPRC of −0.0015, with an interval from −0.0062 to 0.0035, and the robustness-trained variant produced a ΔAUPRC of −0.0031, with an interval from −0.0083 to 0.0022. All intervals included zero, no paired DeLong comparison approached statistical significance, and the smallest raw *p*-value across the 10 pairs was 0.12. The contrast isolating ECG within the fully specified model was also null. Moving from the text model to the tri-modal model yielded ΔAUROC of −0.0002 with *p* = 0.764 and a ΔAUPRC of −0.0019 with an interval from −0.0057 to 0.0019.

**Table 5:** Performance on the MIMIC-IV held-out test set for the primary outcome: death within 30 days of ICU admission (P2, *n* = 9,806, 1,042 events).

Variant	AUROC [95% CI]	AUPRC [95% CI]	ΔAUPRC vs. A [95% CI]	Brier	ECE
A (EHR-only)	0.8969 [0.8878, 0.9062]	0.5809 [0.5537, 0.6087]	reference	0.0645	0.0060
B (EHR+ECG)	0.8964 [0.8867, 0.9055]	0.5778 [0.5509, 0.6053]	−0.0032 [−0.0081, 0.0018]	0.0647	0.0055
C (EHR+text)	0.8971 [0.8878, 0.9062]	0.5813 [0.5548, 0.6093]	+0.0004 [−0.0036, 0.0044]	0.0643	0.0049
D (EHR+ECG+text)	0.8969 [0.8876, 0.9060]	0.5794 [0.5520, 0.6064]	−0.0015 [−0.0062, 0.0035]	0.0645	0.0053
D_robust	0.8972 [0.8883, 0.9065]	0.5778 [0.5515, 0.6058]	−0.0031 [−0.0083, 0.0022]	0.0646	0.0049

95% bootstrap CIs (*n* = 1,000) in brackets. Text modality: radiology reports filed within the 6-h window (19.6% of stays). Pairwise DeLong across all 10 pairs: smallest raw *p* = 0.1210 (B vs. D_robust), Bonferroni-adjusted *p* = 1.000. Every paired AUPRC interval against the EHR-only model contains zero. The contrast that isolates ECG in the fully specified model, C to D, gives ΔAUROC = −0.0002 (DeLong *p* = 0.764) and ΔAUPRC = −0.0019 [−0.0057, 0.0019].

**Figure 4:**
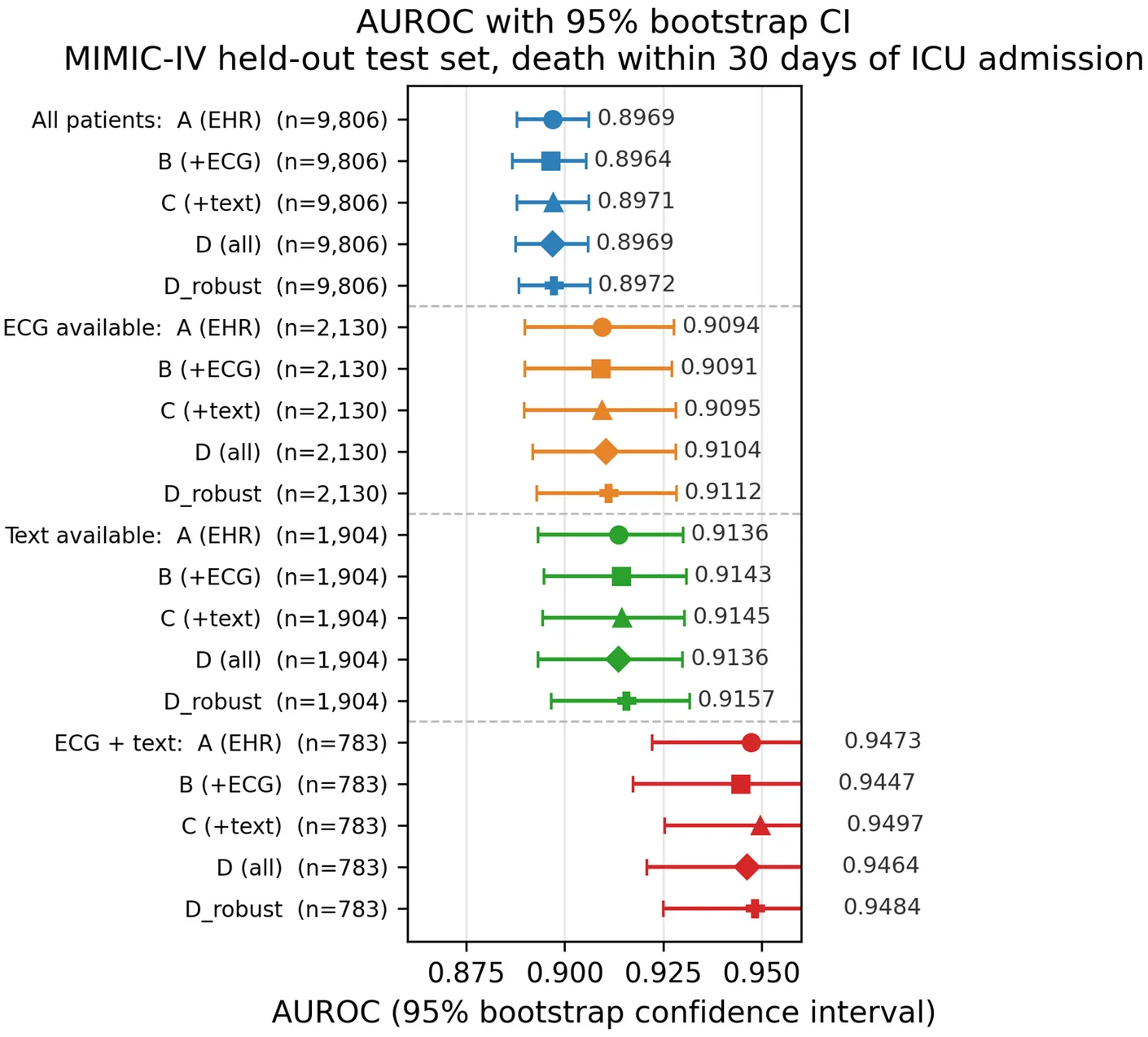
Forest plot of AUROC with 95% bootstrap cis for the five model variants against the primary 30-day outcome, on the MIMIC-IV held-out test set and on the three modality-availability subsets. The variants span 0.8964–0.8972 on the full test set, while absolute performance differs between subsets because of differences in patient populations.

The same pattern was observed at the 48-h and 7-day horizons. At 48 h, ΔAUPRC versus the EHR-only baseline was −0.0064 for ECG, with an interval from −0.0146 to 0.0019, +0.0088 for text, with an interval from 0.0007 to 0.0174, and +0.0050 for the tri-modal model, with an interval from −0.0051 to 0.0147. At 7 days, the corresponding values were −0.0052, +0.0022, and −0.0028. Only one of the twelve contrasts excluded zero, namely the 48-h text model with ΔAUPRC +0.0088. This contrast showed no ranking improvement, with ΔAUROC +0.0006 and DeLong *p* = 0.68, and it did not survive Bonferroni correction across the 10 pairs in its window. The corresponding text effects at 7 and 30 days included zero. Two DeLong tests reached nominal significance at 48 h, both unfavorable to ECG, with *p* = 0.021 for A versus B and *p* = 0.043 for C versus D. Neither survived correction. Overall, these results indicate no reliable benefit or harm from either added modality.

Although neither added modality improved on the EHR-only baseline, text and ECG were not interchangeable. When compared directly on the same patients, the text arm exceeded the ECG arm in AUPRC across all three windows. The difference was +0.0036 at 30 days, with a confidence interval from −0.0015 to 0.0090, +0.0074 at 7 days, with an interval from 0.0002 to 0.0147, and +0.0152 at 48 h, with an interval from 0.0046 to 0.0260. The sign was consistent, and the interval excluded zero at two of the three windows. Both modalities are therefore null against the EHR-only baseline, while text is ranked above ECG when the two are compared directly, a contrast on which the mechanism in Section 4 focuses. The null is better interpreted through its precision than through non-significance: At the primary 30-day horizon, the largest AUPRC improvement compatible with the five modality-addition contrasts was +0.0068, and the largest compatible AUROC improvement was +0.0019. These are confidence limits rather than equivalence margins, and the AUROC limit is comparable to this study’s own training noise, so the AUPRC limit is the more informative one. Supplementary Results S-R6 provides the limits for all 10 pairs and the two conditions they encompass. The decision not to impose a minimum length of stay also affects interpretation: 369 stays ended in death at or before the sixth hour, accounting for 16.3% of all 48-h events and 55 of the 339 in the held-out set. Retaining them avoids conditioning the cohort on surviving the prediction window (Section 2.1). Their influence is greatest at the 48-h horizon and smallest in the primary 30-day analysis, where they account for 5.4% of events.

Two sensitivity analyses supported the same conclusion. A landmark analysis restricted to the 9,751 held-out patients alive at sixth hour reduced discrimination, as expected, because the excluded patients were among the most predictable events. This analysis re-scores the models that have already been fitted. It does not retrain them in a survivor cohort, and 314 of the 369 patients who died at or before hour six remain in the training and validation data. The EHR-only AUROCs were 0.9280, 0.9038, and 0.8917 at 48 h, 7 days, and 30 days, respectively. None of the 12 modality contrasts had an interval excluding zero. The 48-h text contrast shifted to +0.0111, with an interval from −0.0016 to 0.0227, indicating a wider interval around a slightly larger effect rather than convergence toward zero. This analysis shows that the null result was not an artifact of retaining patients who were already dead at the landmark, but a re-scoring cannot demonstrate what the models would have learned without those patients.

The cohort was therefore reconstructed at the landmark, and every model at the 30-day and 48-h horizons was retrained on it, ensuring that patients who died within the window were excluded from both training and evaluation. Removing them left 64,997 stays, 45,500 of which were for training, with the same 9,751 held out, and the outcome still measured from ICU admission. Fifty models were trained, with five seeds for each of the five variants at the 30-day and 48-h horizons. The 7-day horizon was not retrained. The EHR-only model achieved an AUROC of 0.8897 at 30 days and 0.9302 at 48 h, compared to 0.8917 and 0.9280 for the re-scoring, and no paired AUPRC contrast against the EHR-only model had an interval excluding zero at either window (Supplementary Table S9). The one contrast that excludes zero on the full cohort does not survive retraining. The 48-h text effect decreased from +0.0088 on the full cohort, with an interval from 0.0007 to 0.0174, and +0.0111 on the re-scored landmark, with an interval from −0.0016 to 0.0227, to −0.0058 after model refitting, with an interval from −0.0221 to 0.0103. At that window, the paired DeLong test is nominally significant against text rather than for it, with ΔAUROC of −0.0030 and *p* = 0.024, although it does not survive correction across the 10 pairs in that window. With 284 events and a single refit, the estimate is not precise enough to treat the reversal as a finding. Among the 7,729 stays that would have satisfied a minimum 24-h stay criterion, the EHR-only model reached an AUROC of 0.8724, and all modality intervals still included zero.

A null measured on the full cohort could, in principle, be due to dilution: Most patients have neither an ECG nor a note within the window, and their modality channels carry missing-modality embeddings rather than data. The contrasts were therefore repeated on the patients who actually possessed each modality. At the primary 30-day window, these subsets comprise 2,130 stays with an ECG and 197 events, 1,904 stays with a radiology report and 208 events, and 783 stays with both modalities and 66 events. Every one of the twelve paired AUPRC intervals against the EHR-only model on the same patients contains zero in all three subsets. Among stays with a radiology report, adding it changes AUROC by +0.0009, with a paired DeLong *p* = 0.488, and AUPRC by +0.0009, with an interval from −0.0092 to 0.0120. Among stays with an ECG, adding it changes AUROC by −0.0003, with *p* = 0.877, and AUPRC by −0.0005, with an interval from −0.0179 to 0.0161. The full set of contrasts at all three windows is provided in Supplementary Table S7. The absence of a gain is therefore a property of the patients who have the modality, not an artifact of averaging them with patients who do not.

Training behavior provided additional context. Across the 75 models in the main grid, the selected epoch ranged from 1 to 4, with a median of 2. No model reached the 20-epoch budget, and validation AUROC fell from its peak before the patience-5 criterion expired, as reported in Supplementary Table S2 and Supplementary Figure S2. Epoch selection was nonetheless statistically weak: In all 75 runs, the second-best epoch lay within one bootstrap standard error of the selected one. The quantity at stake, approximately 0.001 AUROC, is larger than the modality differences observed in this study and should not be dismissed as negligible; Supplementary Results S-R7 explains why the reported comparisons remain resolvable despite this.

### 3.2 Comparison against clinical scores and a linear model

On the same held-out test set and for the same 30-day outcome, SOFA achieved an AUROC of 0.7310, with a 95% interval from 0.715 to 0.746, while SAPS-II achieved 0.7934, with an interval from 0.779 to 0.806. All learned models outperformed SOFA by approximately 0.17 AUROC and SAPS-II by approximately 0.10 AUROC. Every paired comparison had a DeLong *p* below 10^⁻⁵⁹^, as shown in Table 4 and Figure 5. This discrimination advantage was accompanied by better calibration. SOFA and SAPS-II were poorly calibrated when used as direct probability surrogates, with Brier scores of 0.093 and 0.183, compared with 0.065 for the learned models. Because the clinical scores used their prescribed 24-h window, while the learned models used only the first 6 h, this comparison was conservative with respect to SOFA and SAPS-II.

**Figure 5:**
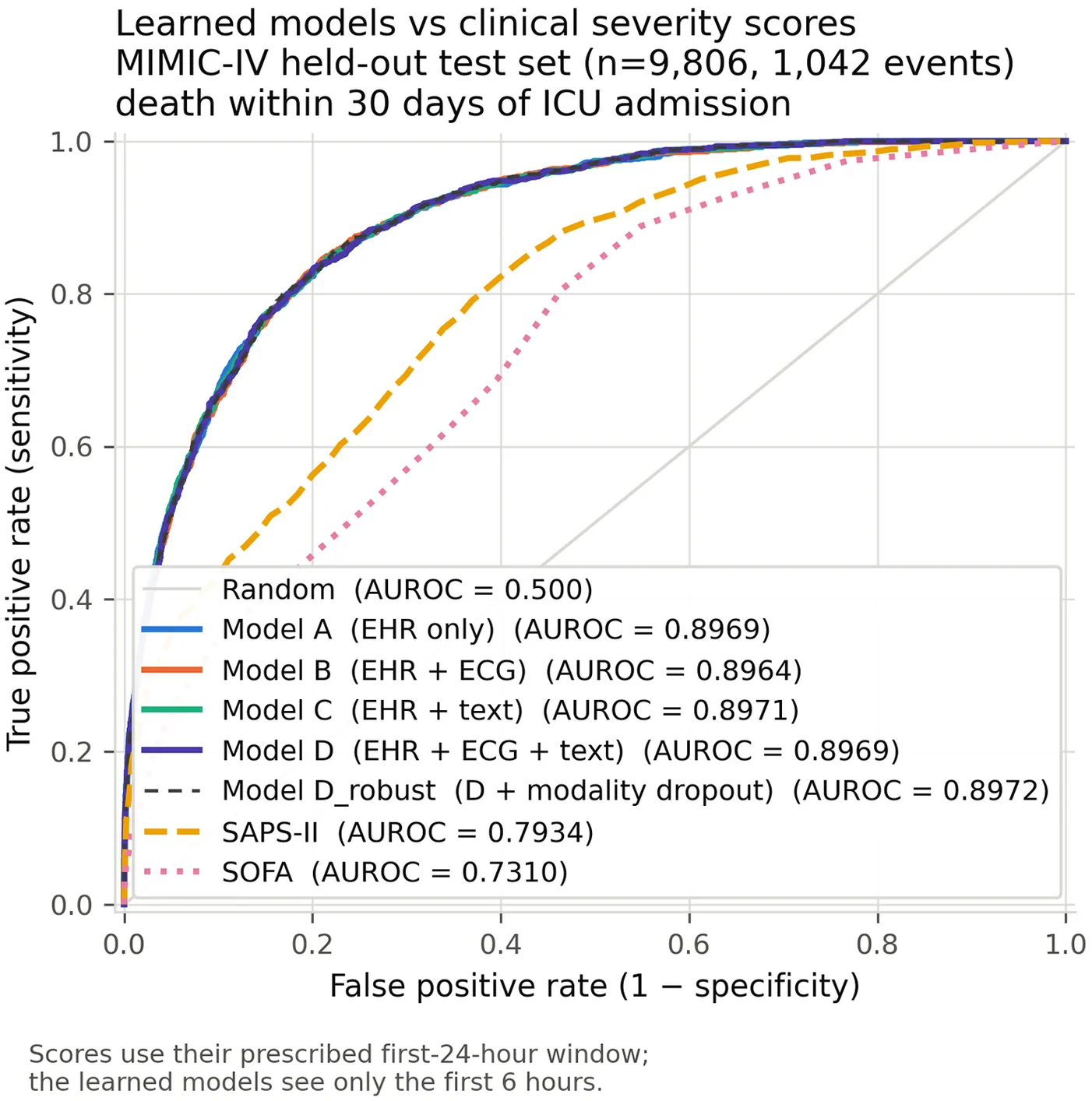
ROC curves on the MIMIC-IV held-out test set for the five learned models (A, B, C, D, and D_robust) and the two clinical risk scores (SOFA, SAPS-II), against the primary 30-day outcome. D_robust is represented as a thin dashed line because it overlaps with the other four. All learned models outperform SAPS-II and SOFA by ΔAUROC ≈ +0.10 and +0.17, respectively (paired DeLong *p* < 10⁻⁵⁹).

The more informative baseline was a linear model trained on the same inputs as the EHR-only neural model. L2-penalized logistic regression fitted on the identical 1,587-column matrix and the same patient-level split reached AUROC values of 0.8844 for 30-day mortality, 0.8991 for 7-day mortality, and 0.9249 for 48-h mortality. Expected calibration error was 0.013, 0.009, and 0.003, indicating good calibration without *post-hoc* adjustment. The selected penalty strength remained within the validation search grid at every horizon.

The comparison with the neural model must account for the five-seed ensemble. A single neural model averaged an AUROC of 0.8908 at the primary horizon, compared with 0.8969 for the ensemble. Thus, 0.0061 of the apparent gain reflected ensembling rather than architecture. Bagging the logistic regression over five fits did not improve performance, yielding 0.8843 compared with 0.8844, which is expected for a model that is not variance-limited. Under a matched five-fit comparison, the neural architecture improved AUROC by 0.0126 at 30 days, 0.0126 at 7 days, and 0.0123 at 48 h. At 30 days, the 95% interval was 0.0083 to 0.0168. The corresponding AUPRC gains were 0.0373, 0.0335, and 0.0347, as reported in Table 4 and Supplementary Figure S3. In the one-fit comparison, which better isolates architecture from ensembling, the neural model improved AUROC over logistic regression by 0.0065 at 30 days, 0.0057 at 7 days, and 0.0047 at 48 h. The corresponding intervals were 0.0022–0.0105, 0.0002–0.0111, and −0.0025–0.0123.

The neural architecture therefore contributed meaningfully beyond the structured EHR feature set, but the gain was modest. Its advantage was about 1.3 AUROC points over a linear model using the same inputs, with roughly half attributable to ensembling. Unlike the modality effects, this difference had intervals excluding zero at all three horizons. It is not expressed as a multiple of the modality effects because architecture comparisons and modality ablations test different contrasts, use different metrics, and are subject to different multiplicity corrections.

### 3.3 SHAP-based interpretation

The highest-ranked structured EHR features in model A were clinically coherent and reflected established markers of ICU mortality. The leading predictor was age, with a mean absolute SHAP of 0.0192, followed by last GCS motor response at 0.0072, maximum GCS motor response at 0.0069, last GCS eye opening at 0.0062, mean respiratory rate at 0.0058, and last oxygen saturation by pulse oximetry at 0.0057. Maximum GCS eye opening, the Richmond agitation-sedation scale at its maximum and its last value, minimum oxygen saturation, minimum non-invasive mean arterial pressure, and a temperature count column completed the top 12, as shown in Figure 3. The ranking was essentially unchanged between model A and the EHR branch of model D, indicating that multimodal fusion did not alter the main structured predictors.

Two aspects of this ranking are important. First, total ICU length of stay no longer appeared among the predictors. It represents the entire ICU stay and is not available within the prediction window. Second, several highly ranked variables were counts rather than measured values. These columns captured how often a quantity was charted during the first 6 h. A linear baseline fitted only on the 317 count columns, with all measured values removed, reached an AUROC of 0.7900 and an AUPRC of 0.3422. This was above SOFA at 0.7310 and close to SAPS-II at 0.7934. Removing count columns from the full matrix reduced AUROC by 0.0109, with an interval from −0.0157 to −0.0062 (Supplementary Table S3). Measurement frequency was therefore a substantial signal. It reflects the care process, since unstable patients are measured more often, and should be treated as a transportability concern. The same ablation showed that adding the length-of-stay feature back to the rebuilt matrix changed the linear baseline by only −0.0017 AUROC, with an interval from −0.0026 to −0.0009.

Model D was interpreted in the same form in which it generated predictions. Attribution was computed over the concatenated EHR matrix, ECG matrix, text embedding, and two availability indicators, using each patient's own note and modality masks. The attribution wrapper reproduced the evaluation pipeline's per-patient probabilities with a mean absolute difference below 10^⁻⁶^, so the explanations correspond to the model underlying Table 5. In the pooled ranking across branches, the highest ECG input was the printed statement “sinus rhythm,” which ranked 106th of 1,827 features in Figure 6. This was an order of magnitude below the leading structured features. The first derived ECG measurement, heart rate, ranked 597th. No ECG input appeared in the top 20. Within the ECG branch, the leading features were “sinus rhythm,” “sinus tachycardia,” “abnormal ECG,” heart rate, and QT interval, as shown in Supplementary Figure S1. Thus, the model read the ECG channel but assigned it little weight in the full prediction context. The attributions cannot fully distinguish whether the model used ECG content or simply the fact that an ECG was obtained. The results are therefore interpreted as bounds rather than causal claims. In the complete held-out set, the ECG availability indicator ranked 16th, with a mean absolute SHAP of 0.0035, and the text availability indicator ranked 18th, with 0.0034. Both were above every individual ECG measurement.

**Figure 6:**
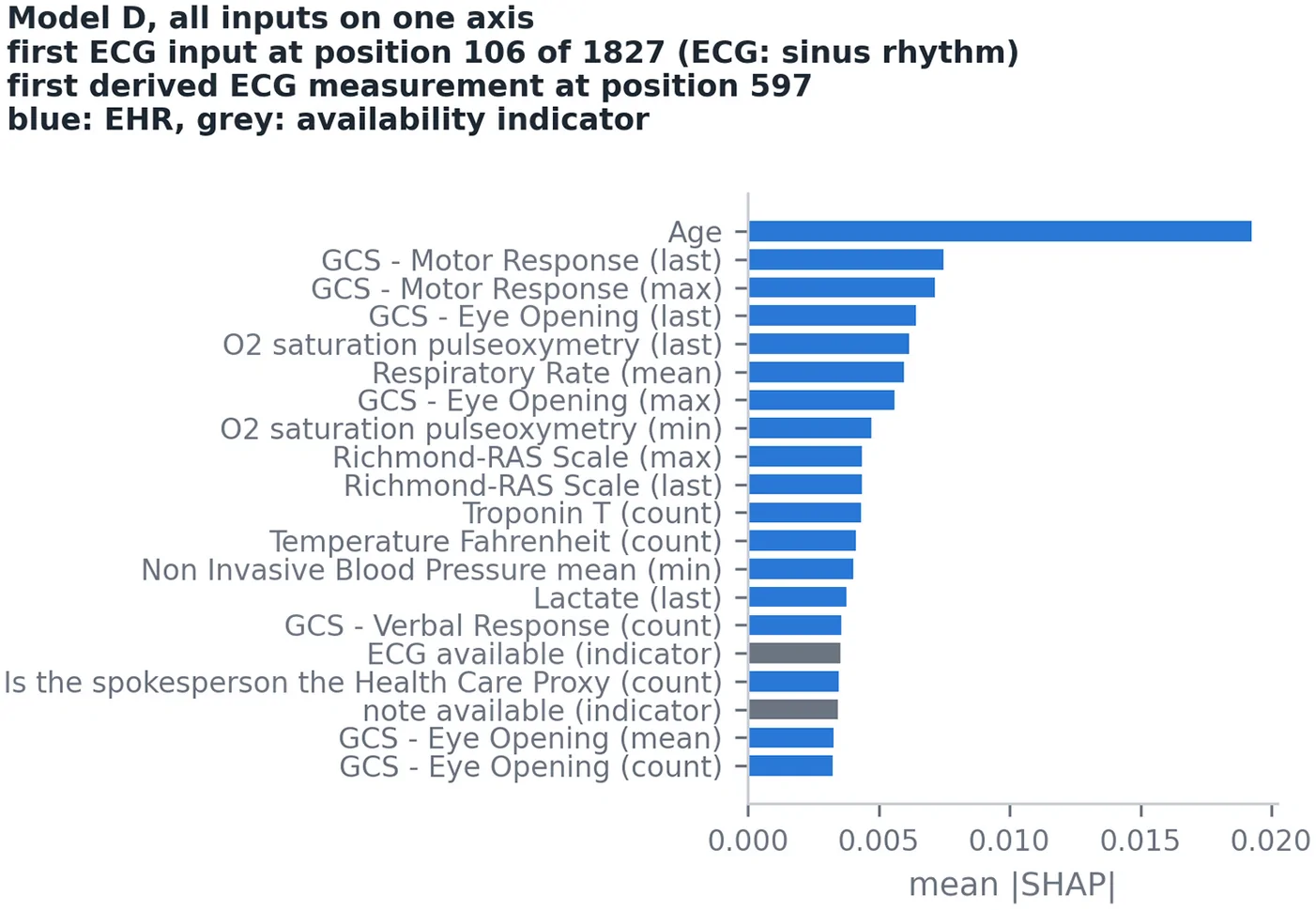
Model D's 20 highest-ranked inputs are displayed with both branches on one axis, allowing the position of an ECG feature against the structured record to be read directly rather than recomputed from a table. Attributions are computed using each patient's note and own availability masks, across the concatenation of the EHR matrix, ECG matrix, text embedding, and two availability indicators. The wrapper reproduces the evaluation pipeline's probabilities exactly. No ECG input appears in the top 20; the highest, the printed statement “sinus rhythm,” ranks 106th of 1,827, and the first derived measurement ranks 597th, while the indicators of ECG and text availability rank 16th and 18th.

However, a stay without an ECG carries a constant imputed vector, so the availability indicator and the 110 ECG columns vary together when the mask changes. Three explainer settings were used to address this issue, with full results in Supplementary Results S-R5. Two findings were stable across all settings. The model read the ECG channel, and no ECG input approached the top of the ranking when modality availability was allowed to vary. Per-column rankings also understate branch-level contributions. Summed across 110 columns, the ECG block carried 0.0102 compared with 0.0035 for its indicator. The text embedding carried 0.0020 across 128 dimensions, while the structured block carried 0.6599. The best-ranked text dimension was 1,073rd of 1,827 features.

These attribution results sharpen the negative modality finding. Among patients with an ECG, the model read the channel and ranked its leading printed statement thirteenth of 1,827 inputs across all branches, yet adding ECG did not improve prediction. In this subgroup, ΔAUPRC was −0.0005, with an interval from −0.0179 to 0.0161. A likely explanation is that rate, rhythm, and hemodynamic state were already represented in the structured record during the same 6-h window. The 12-lead machine summary therefore repeated information already available to the model.

Two fidelity checks assessed whether the attributions described the trained model. In 2,000 held-out patients, summed expected-gradient attributions tracked the completeness identity with Pearson correlations ranging from 0.9463 to 0.9664 across the five seeds of model D. The mean absolute residual was between 0.28 and 0.35 of the reconstructed quantity. Across independent explainer draws, it decreased to 0.15–0.16 at 200 gradient samples, while rankings remained stable with a Spearman *ρ* of at least 0.9977. Because individual explanations would carry substantial noise, all rankings are reported as means over 2,000 patients and five seeds. A second check replaced the k most strongly attributed features with their training means. This change affected predicted probabilities 38.6 times more than replacing k randomly chosen observed features, and 6.45 times more at *k* = 100. The features identified by attribution were therefore the features actually used by the network.

### 3.4 External validation across three independent cohorts

The same model checkpoints trained on MIMIC-IV without exposure to external cohorts were evaluated on eICU-CRD v2.0, MC-MED, and HiRID. None of these databases supplies ECG measurements or clinical notes, so this analysis evaluates the transfer of the structured EHR backbone while masking both extra modalities, rather than assessing the incremental contribution of ECG or text. Calibration metrics used the temperature fitted on the MIMIC-IV validation split for each variant and applied it unchanged to every external cohort. No external outcome was used for model fitting or recalibration. All model variants operated with missing-modality embeddings. Differences between variants therefore reflected only how the shared EHR backbone had been shaped during training. External labels were reconstructed at each horizon using each cohort’s own timing information. A limitation of this reconstruction is that neither HiRID nor MC-MED provides a death timestamp. For these two cohorts, time to event was therefore derived from length of stay. This variable did not enter the model, but it did define the outcome window used for evaluation.

Because two of the three external outcome definitions are approximate, the sensitivity of the results to plausible errors in the derived event time was measured instead of being assumed. Holding the predictions fixed and rebuilding the labels from a perturbed event time leaves the primary 30-day rows unchanged under every shift tested: On HiRID, the label for 0 stays the same when the assumed time of death is moved 48 h later; on MC-MED, the label for 1 stays the same; and on eICU-CRD, where the event time is exact, 47 of 138,868 events change, with AUROC moving by +0.0006. The 48-h rows behave differently, and the cohort with an exact event time shows that this is a property of the window rather than of the approximation: On eICU-CRD, a 24-h shift moves 1,917 of 5,055 events and AUROC by +0.0518. The same shift moves 454 of 1,204 events on HiRID (+0.0287 AUROC), and a 6-h shift moves 33 of 106 on MC-MED (+0.0340). The 48-h external rows therefore carry an uncertainty in the event time that the primary 30-day rows do not, while the primary 30-day result is insensitive to the event-time perturbations tested here (Supplementary Table S8).

For the primary 30-day outcome, the EHR-only model achieved AUROC values of 0.8333 on HiRID, 0.7744 on MC-MED, and 0.7268 on eICU-CRD. The corresponding cohort sizes and event rates were 33,666 with 6.1%, 2,575 with 10.0%, and 138,868 with 9.2%. Calibration gaps were −0.0184, +0.0159, and +0.0187. Across 48-h, 7-day, and 30-day horizons, the EHR-only model reached AUROC values of 0.8540, 0.8115, and 0.8333 on HiRID. The corresponding values were 0.7835, 0.7801, and 0.7744 on MC-MED, and 0.7597, 0.7120, and 0.7268 on eICU-CRD. All five variants were evaluated for every cohort and horizon, yielding 45 external rows in Table 6. At the primary horizon, AUROC ranged from 0.8023 to 0.8333 on HiRID, from 0.7727 to 0.7754 on MC-MED, and from 0.7219 to 0.7268 on eICU-CRD. Discrimination decreased by 6–17 AUROC points relative to the development cohort. This loss was largely attributable to feature attrition, especially in eICU-CRD, where only 18.4% of the feature matrix could be reconstructed. Nevertheless, the models remained informative in all three external cohorts. Aggregate calibration gaps concealed deployment-relevant structure, which is examined in Section 3.9.

**Table 6:** External validation under fixed outcome windows.

Cohort	Outcome window	Variant	*N*	Event rate	AUROC [95% CI]	AUPRC	Brier	Calibration gap	Gap before rescale
MIMIC-IV (internal)	30 days (primary)	A	9,806	10.6%	0.8969 [0.8878, 0.9062]	0.5809	0.0645	−0.0050	—
MIMIC-IV (internal)	30 day (primary)	D	9,806	10.6%	0.8969 [0.8876, 0.9060]	0.5794	0.0645	−0.0013	—
HiRID	48 h	A	33,666	3.6%	0.8540 [0.8437, 0.8634]	0.3720	0.0276	−0.0112	−0.0097
HiRID	48 h	B	33,666	3.6%	0.8313 [0.8195, 0.8423]	0.3568	0.0278	−0.0132	−0.0089
HiRID	48 h	C	33,666	3.6%	0.8718 [0.8624, 0.8801]	0.3804	0.0279	−0.0077	−0.0038
HiRID	48 h	D	33,666	3.6%	0.8778 [0.8687, 0.8861]	0.3734	0.0281	−0.0068	−0.0048
HiRID	48 h	D_robust	33,666	3.6%	0.8723 [0.8628, 0.8810]	0.3868	0.0280	−0.0028	−0.0003
HiRID	7 days	A	33,666	5.4%	0.8115 [0.8014, 0.8225]	0.3653	0.0423	−0.0168	−0.0262
HiRID	7 days	B	33,666	5.4%	0.8079 [0.7977, 0.8191]	0.3483	0.0432	−0.0183	−0.0244
HiRID	7 days	C	33,666	5.4%	0.8089 [0.7984, 0.8200]	0.3607	0.0424	−0.0132	−0.0215
HiRID	7 days	D	33,666	5.4%	0.8141 [0.8039, 0.8245]	0.3469	0.0431	−0.0135	−0.0171
HiRID	7 days	D_robust	33,666	5.4%	0.8126 [0.8026, 0.8237]	0.3552	0.0430	−0.0142	−0.0166
HiRID	30 days (primary)	A	33,666	6.1%	0.8333 [0.8241, 0.8428]	0.3818	0.0472	−0.0184	−0.0242
HiRID	30 days (primary)	B	33,666	6.1%	0.8023 [0.7921, 0.8127]	0.3622	0.0473	−0.0090	−0.0035
HiRID	30 days (primary)	C	33,666	6.1%	0.8276 [0.8183, 0.8374]	0.3751	0.0476	−0.0184	−0.0253
HiRID	30 days (primary)	D	33,666	6.1%	0.8323 [0.8231, 0.8416]	0.3812	0.0467	−0.0063	−0.0148
HiRID	30 days (primary)	D_robust	33,666	6.1%	0.8035 [0.7935, 0.8134]	0.3557	0.0477	−0.0115	−0.0111
MC-MED	48 h	A	2,575	4.1%	0.7835 [0.7345, 0.8305]	0.1870	0.0412	+0.0102	+0.0102
MC-MED	48 h	B	2,575	4.1%	0.7853 [0.7345, 0.8318]	0.1883	0.0431	+0.0170	+0.0165
MC-MED	48 h	C	2,575	4.1%	0.7764 [0.7265, 0.8234]	0.1834	0.0424	+0.0091	+0.0091
MC-MED	48 h	D	2,575	4.1%	0.7815 [0.7317, 0.8276]	0.1876	0.0446	+0.0183	+0.0160
MC-MED	48 h	D_robust	2,575	4.1%	0.7809 [0.7328, 0.8265]	0.1894	0.0460	+0.0193	+0.0204
MC-MED	7 days	A	2,575	6.8%	0.7801 [0.7428, 0.8141]	0.2426	0.0619	+0.0107	+0.0019
MC-MED	7 days	B	2,575	6.8%	0.7790 [0.7417, 0.8131]	0.2438	0.0632	+0.0160	+0.0113
MC-MED	7 days	C	2,575	6.8%	0.7791 [0.7413, 0.8138]	0.2458	0.0630	+0.0154	+0.0066
MC-MED	7 days	D	2,575	6.8%	0.7745 [0.7368, 0.8091]	0.2372	0.0643	+0.0182	+0.0149
MC-MED	7 days	D_robust	2,575	6.8%	0.7784 [0.7414, 0.8124]	0.2400	0.0634	+0.0186	+0.0155
MC-MED	30 days (primary)	A	2,575	10.0%	0.7744 [0.7446, 0.8047]	0.3234	0.0819	+0.0159	+0.0052
MC-MED	30 days (primary)	B	2,575	10.0%	0.7733 [0.7434, 0.8042]	0.3246	0.0839	+0.0232	+0.0213
MC-MED	30 days (primary)	C	2,575	10.0%	0.7743 [0.7436, 0.8045]	0.3214	0.0831	+0.0171	+0.0070
MC-MED	30 days (primary)	D	2,575	10.0%	0.7754 [0.7449, 0.8058]	0.3177	0.0849	+0.0300	+0.0192
MC-MED	30 days (primary)	D_robust	2,575	10.0%	0.7727 [0.7425, 0.8034]	0.3223	0.0833	+0.0208	+0.0176
eICU-CRD	48 h	A	138,868	3.6%	0.7597 [0.7514, 0.7676]	0.2978	0.0348	+0.0361	+0.0284
eICU-CRD	48 h	B	138,868	3.6%	0.7558 [0.7474, 0.7636]	0.2937	0.0356	+0.0361	+0.0346
eICU-CRD	48 h	C	138,868	3.6%	0.7443 [0.7357, 0.7524]	0.2850	0.0388	+0.0482	+0.0449
eICU-CRD	48 h	D	138,868	3.6%	0.7450 [0.7366, 0.7533]	0.2815	0.0417	+0.0552	+0.0510
eICU-CRD	48 h	D_robust	138,868	3.6%	0.7491 [0.7405, 0.7571]	0.2957	0.0396	+0.0503	+0.0521
eICU-CRD	7 days	A	138,868	6.8%	0.7120 [0.7058, 0.7185]	0.2831	0.0597	+0.0306	+0.0086
eICU-CRD	7 days	B	138,868	6.8%	0.7000 [0.6938, 0.7065]	0.2776	0.0601	+0.0336	+0.0187
eICU-CRD	7 days	C	138,868	6.8%	0.7140 [0.7080, 0.7205]	0.2816	0.0601	+0.0293	+0.0111
eICU-CRD	7 days	D	138,868	6.8%	0.7166 [0.7106, 0.7230]	0.2880	0.0611	+0.0428	+0.0358
eICU-CRD	7 days	D_robust	138,868	6.8%	0.7064 [0.7002, 0.7127]	0.2755	0.0606	+0.0366	+0.0288
eICU-CRD	30 days (primary)	A	138,868	9.2%	0.7268 [0.7217, 0.7317]	0.3101	0.0765	+0.0187	+0.0069
eICU-CRD	30 days (primary)	B	138,868	9.2%	0.7268 [0.7220, 0.7319]	0.3143	0.0774	+0.0276	+0.0287
eICU-CRD	30 days (primary)	C	138,868	9.2%	0.7225 [0.7174, 0.7276]	0.3070	0.0767	+0.0179	+0.0061
eICU-CRD	30 days (primary)	D	138,868	9.2%	0.7219 [0.7170, 0.7270]	0.3011	0.0783	+0.0263	+0.0137
eICU-CRD	30 days (primary)	D_robust	138,868	9.2%	0.7252 [0.7203, 0.7300]	0.3127	0.0776	+0.0275	+0.0257

Each model is scored against the outcome window for which it was trained. External event times come from each cohort's own records (Methods). AUROC and AUPRC use the mean predicted probability. Brier and CalGap use the temperature-rescaled probability, based on one temperature fitted on the MIMIC-IV validation split and never on external data. The final column gives the same gap computed from the raw ensemble probability, before that rescale (Section 3.9). At the primary horizon, the five variants lie within 0.0049 AUROC of each other on eICU-CRD (A 0.7268, B 0.7268, C 0.7225, D 0.7219, D_robust 0.7252) and within 0.0027 on MC-MED (0.7727–0.7754), i.e., within the bootstrap interval in both cohorts; per-variant values for all cohorts and windows are given in the released result tables.

Internally, the variants were indistinguishable. Externally, they were separated, but this separation could not be attributed to the added modalities. No internal pairwise comparison survived Bonferroni correction at any horizon. Externally, 49 of 90 paired comparisons survived correction within their cohort and window, which primarily reflects cohort size. Among these, 27 differences were below 0.01 AUROC, a magnitude that lies within the variation produced by seed composition alone. Since no external cohort provided ECG or text at inference, the observed differences could not arise from those inputs. To separate training effects from ensemble composition, each variant’s ensemble was re-formed from subsets of its own seeds on HiRID. Seed composition alone shifted a variant by as much as the between-variant differences. External seed variation was about eight times larger than internal seed variation, and only 4 of 20 between-variant comparisons retained the same sign across all 100 re-compositions, as reported in Supplementary Results S-R2. For this reason, between-variant differences are not interpreted as effects of the modalities used during training. At the primary horizon on HiRID, the order was A at 0.8333, D at 0.8323, C at 0.8276, D_robust at 0.8035, and B at 0.8023. One broader finding remained clear: The external performance of a five-seed ensemble depended far more on which seeds it contained than internal performance did. A single externally validated number can therefore conceal uncertainty that internal validation does not reveal (30). Reporting a single variant per cohort would obscure this.

The transfer of the architectural advantage was also assessed by applying the linear baseline to the external cohorts without refitting (Supplementary Table S1). The advantage transferred, but not uniformly and not always with the same sign. It was largest on eICU-CRD, with AUROC gains of +0.0345, +0.0310, and +0.0388 at 48 h, 7 days, and 30 days. On MC-MED, the corresponding differences were −0.0012, +0.0173, and +0.0129. On HiRID, they were +0.0286, −0.0112, and +0.0119, with the linear model outperforming the five-seed neural ensemble at 7 days. This comparison was generous to the neural model because it used one linear fit against a five-model ensemble, which was worth +0.0061 internally. The deep model therefore helped most where transfer was weakest and showed no reliable advantage where transfer was strongest.

Two design choices account for most of the external picture, and both are quantified rather than asserted. Supplying the total ICU length of stay to the model inflates MC-MED at the 48-h window: The field mapped onto it is total hospital length of stay, which is short for a patient who dies within 48 h. Without it, the cohort scores an AUROC of 0.7835 rather than 0.9418. That field is also measured from emergency department arrival, while the models predict death within each horizon after ICU admission, an anchor shift that matters least at 30 days. The second choice is the missing-value rule. Imputing with raw zero before standardization at training time, while filling unmappable columns with the training mean at inference, leaves most of each eICU-CRD input vector filled by a rule the model never encountered, since only 18.4% of that matrix is mappable. Re-scoring the same models on the same cohort with only that rule reverted gives an AUROC of 0.5939 and a calibration gap of +0.3258, compared to 0.7268 and +0.0187 when both sides use one rule: a single factor worth 0.1329 AUROC.

### 3.5 Demographic fairness

A subgroup audit was conducted on the MIMIC-IV held-out test set for the primary 30-day outcome, as shown in Figure 7 and Table 7. The analysis indicated moderate performance heterogeneity. Against an overall AUROC of 0.8969, discrimination was lowest in patients aged 80 years or older, with an AUROC of 0.8527 and a 95% interval from 0.8288 to 0.8745. This subgroup included 1,840 patients, was flagged for being more than 0.03 below the overall value, and had the highest event rate at 17.1%. The highest discrimination was observed in patients aged 18–49 years, with an AUROC of 0.9545 and an interval from 0.9417 to 0.9676 among 1,968 patients. Thus, performance decreased most in the oldest and highest-risk segment. Race-stratified AUROC estimates ranged from 0.8775 to 0.9138. Calibration gaps exceeded 0.02 in the Black or African American subgroup, with +0.0243 among 895 patients, and in the Other or Unknown subgroup, with −0.0303 among 1,826 patients. Both groups were flagged for *post-hoc* recalibration before deployment. By ICU type, AUROC ranged from 0.8567 to 0.9240, while sex differences were modest. In total, five subgroups were flagged. These exploratory estimates were not multiplicity-adjusted, and intervals for smaller subgroups overlapped the overall estimate.

**Figure 7:**
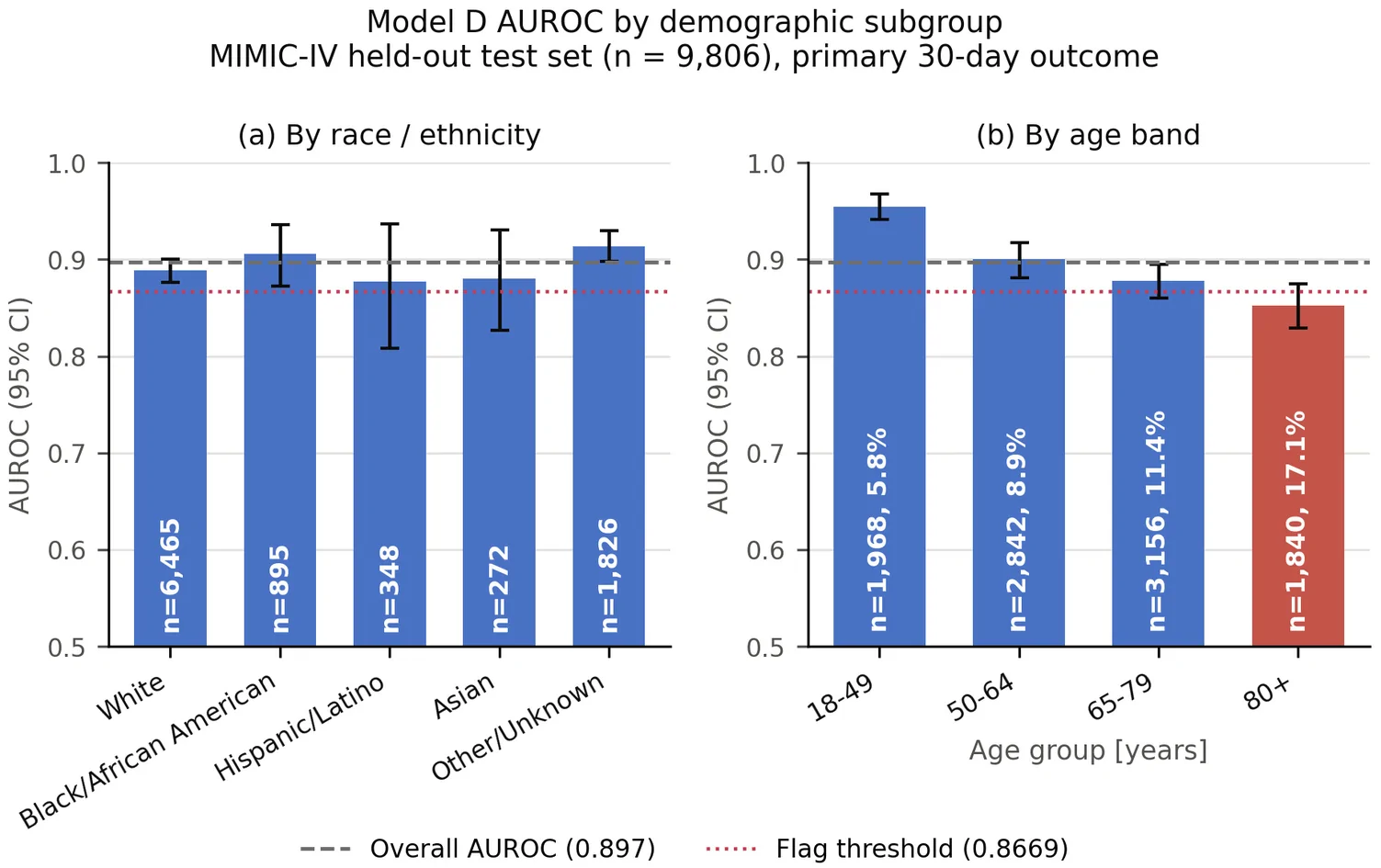
Fairness audit of model D on the MIMIC-IV held-out test set (*n* = 9,806) against the primary 30-day outcome: AUROC by race or ethnicity **(a)** and by age band **(b)**. The dashed line represents the overall AUROC (0.8969), and the dotted line indicates the flag threshold 0.03 below it (0.8669); the flagged bar is drawn in red. The 80+ and MICU/SICU subgroups are flagged for a drop in AUROC, and the Black or African American, Other or Unknown, and other-ICU-type subgroups are flagged for calibration drift, with subgroup intervals overlapping the overall estimate.

**Table 7:** Fairness audit (model D, MIMIC-IV held-out test set, primary 30-day outcome).

Subgroup type	Subgroup	*N*	Event rate (%)	AUROC [95% CI]	AUPRC	Calibration gap	Flagged
overall	All patients	9,806	10.6	0.8969 [0.8882, 0.9058]	0.5794	−0.0013	—
race	White	6,465	9.4	0.8884 [0.8765, 0.9004]	0.5228	+0.0036	—
race	Black/African American	895	7.7	0.9060 [0.8723, 0.9359]	0.5577	+0.0243	Calibration
race	Hispanic/Latino	348	8.1	0.8775 [0.8079, 0.9365]	0.5140	+0.0066	—
race	Asian	272	12.1	0.8806 [0.8265, 0.9306]	0.5449	−0.0180	—
race	Other/Unknown	1,826	16.7	0.9138 [0.8979, 0.9294]	0.7176	−0.0303	Calibration
gender	Male	5,555	10.2	0.9025 [0.8903, 0.9137]	0.5763	−0.0024	—
gender	Female	4,251	11.1	0.8898 [0.8750, 0.9037]	0.5848	+0.0002	—
age_group	18–49	1,968	5.8	0.9545 [0.9417, 0.9676]	0.6472	+0.0039	—
age_group	50–64	2,842	8.9	0.9007 [0.8813, 0.9177]	0.5681	+0.0003	—
age_group	65–79	3,156	11.4	0.8783 [0.8598, 0.8948]	0.5482	−0.0048	—
age_group	80+	1,840	17.1	0.8527 [0.8288, 0.8745]	0.6014	−0.0032	AUROC
icu_type	MICU	1,862	16.7	0.8673 [0.8460, 0.8866]	0.6013	−0.0067	—
icu_type	MICU/SICU	1,559	12.4	0.8567 [0.8297, 0.8811]	0.5217	−0.0045	AUROC
icu_type	CVICU	1,758	3.0	0.9240 [0.8856, 0.9510]	0.4907	+0.0045	—
icu_type	SICU	1,543	13.1	0.8849 [0.8606, 0.9096]	0.6263	−0.0157	—
icu_type	CCU	1,105	12.8	0.8891 [0.8592, 0.9157]	0.6130	+0.0074	—
icu_type	TSICU	1,160	10.3	0.9005 [0.8734, 0.9268]	0.6238	−0.0023	—
icu_type	Other	819	2.4	0.9108 [0.8589, 0.9500]	0.1944	+0.0215	Calibration

Flags: AUROC below the overall value minus 0.03, i.e.,  < 0.8669; calibration gap |·| > 0.02. Discrimination is computed from the mean ensemble probability and calibration from the temperature-scaled one, as in Table 6 and Section 2.4.

### 3.6 Robustness to missing modalities

Model D and the robustness-trained variant D_robust were evaluated against the primary 30-day outcome under four perturbations applied at inference time: the ECG channel masked, the text channel masked, both masked, and the ECG measurements degraded with Gaussian noise. Neither variant was sensitive to any of these perturbations. AUROC stayed within 0.0005 of the unperturbed value for model D, ranging from 0.8964 to 0.8969, and within 0.0005 for D_robust, ranging from 0.8967 to 0.8974. Masking both non-tabular modalities changed model D’s AUROC by 0.0004 and its AUPRC by 0.0007, from 0.5794 to 0.5787, which is smaller than the bootstrap resolution of either metric for this cohort. Removing the ECG channel slightly improved model D’s expected calibration error, from 0.0053 to 0.0043, indicating that modality-dropout training conferred no measurable calibration advantage at this horizon either. The degraded-ECG scenario was indistinguishable from no perturbation across all metrics: It adds Gaussian noise with a standard deviation of 0.1 to measurements that are already standardized, and the two waveform parameters in its configuration are inert because this study has no waveform branch. These results are consistent with the fused model relying almost entirely on its structured EHR branch, which is also why removing the other two channels barely affected it.

### 3.7 Effect of the outcome window

The outcome window is a design choice rather than an inherent property of the data. Therefore, the full set of model variants was trained for three fixed outcome windows, as shown in Table 8 and Supplementary Figure S5, and compared with an open-ended outcome definition. Achievable performance depended strongly on the outcome window. The EHR-only model reached an AUROC of 0.9371 at 48 h, with a 95% interval from 0.9256 to 0.9484. At 7 days, AUROC was 0.9106, with an interval from 0.9000 to 0.9207. At 30 days, AUROC was 0.8969, with an interval from 0.8878 to 0.9062. The corresponding event rates were 3.5%, 6.9%, and 10.6%. Because these models were trained separately, this comparison combines the effects of the label and retraining. When one model was held fixed and only the label changed, the same pattern persisted, with AUROCs of 0.9374, 0.9125, and 0.8969.

**Table 8:** Effect of the outcome window.

Outcome definition	Events in test set (rate)	AUROC, EHR-only [95% CI]	AUPRC, EHR-only	Brier	*Δ*AUPRC,+text [95% CI]	*Δ*AUPRC, tri-modal [95% CI]
Death within 48 h	339 (3.5%)	0.9371 [0.9256, 0.9484]	0.5455	0.0223	+0.0088 [0.0007, 0.0174]	+0.0050 [−0.0051, 0.0147]
Death within 7 days	681 (6.9%)	0.9106 [0.9000, 0.9207]	0.5444	0.0446	+0.0022 [−0.0028, 0.0077]	−0.0028 [−0.0096, 0.0050]
Death within 30 days (primary)	1,042 (10.6%)	0.8969 [0.8878, 0.9062]	0.5809	0.0645	+0.0004 [−0.0036, 0.0044]	−0.0015 [−0.0062, 0.0035]

Same cohort, same 6-h features, same five variants; only the definition of the event changes. ΔAUPRC is the paired difference against model A on identical patients (1,000-resample bootstrap).

Fixing the primary window at 30 days instead of using an open-ended outcome changed the same model only slightly, from 0.8958 to 0.8969. Thus, the fixed window mainly provides a clearer clinical task and interpretable shorter-horizon analyses, rather than a materially different primary estimate. The same temporal gradient appeared within a single model. Among 8,723 held-out stays that survived to hospital discharge, the tri-modal model distinguished deaths within 48 h with an AUROC of 0.9517 (Supplementary Table S4). AUROC declined to 0.8931 for deaths occurring between 2 and 7 days, 0.8523 for deaths between 7 and 30 days, and 0.8382 for deaths after day 30. Mean predicted risk among events also fell from 0.5604 to 0.2432. The model therefore assigned systematically lower risk to later deaths, consistent with predictions based on the first 6 h of acute physiology.

The marginal value of additional modalities was null at the primary 30-day window and did not become consistently positive at shorter horizons. The small positive effects reported previously appeared only with an open-ended outcome and with total ICU length of stay included among predictors. They were not reproduced after both design choices were corrected.

### 3.8 Clinical utility

Discrimination and calibration describe predictive performance, but they do not indicate whether acting on predictions is worthwhile. Therefore, we evaluated deployment-relevant operating characteristics, reported in Supplementary Table S5, and estimated net benefit using decision-curve analysis against treating all patients or treating none (31), as shown in Supplementary Figure S6.

In the MIMIC-IV held-out set, identifying 90% of 30-day deaths required flagging 37 of every 100 patients, with a positive predictive value of 0.26. With a 10% alert budget, the model identified 52% of deaths with a positive predictive value of 0.55, corresponding to about two patients reviewed for each death identified. In the eICU-CRD, the same high-sensitivity strategy required flagging 75 of every 100 patients, making it impractical. With a 10% alert budget, the model identified 34% of deaths with a positive predictive value of 0.32. HiRID and MC-MED showed intermediate results.

In eICU-CRD, a comparison with APACHE IVa was possible because the database includes APACHE IVa predictions. Among 113,127 stays with APACHE IVa scores, APACHE IVa achieved AUROC 0.8706 and AUPRC 0.4792, compared with 0.7209 and 0.2889 for our model. It also demonstrated higher net benefit at every examined threshold, as shown in Supplementary Figure S7. This comparison is partly conservative and partly unfavorable. APACHE IVa uses 24 h of information, whereas our model uses six, but APACHE IVa was evaluated against an outcome for which it was not originally designed.

Decision-curve analysis supported the model more favorably. Acting on its predictions produced positive net benefit and exceeded both default strategies across relevant threshold ranges in all cohorts. This indicates actionable information, but not sufficient clinical utility. Outside the development institution, the alert burden needed for desirable sensitivity remains too high.

### 3.9 Calibration in every cohort

Calibration was assessed at the primary 30-day horizon for the MIMIC-IV held-out set and all three external cohorts. The analysis used the same per-patient predictions as the main performance tables and summarized calibration by decile of predicted risk, as shown in Figure 8 and Table 9. The reliability diagram for eICU-CRD, the largest external cohort, is presented separately in Supplementary Figure S4.

**Figure 8:**
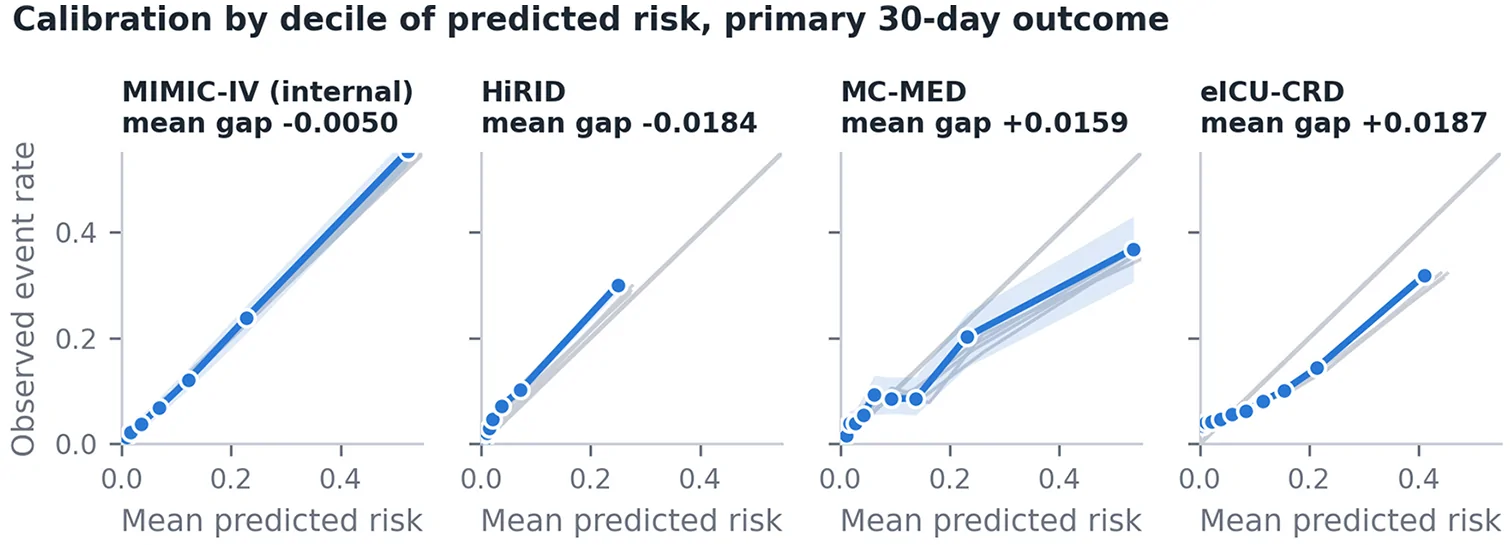
Calibration by decile of predicted risk for the EHR-only model across all four cohorts for the primary 30-day outcome (MIMIC-IV held-out, HiRID, MC-MED, and eICU-CRD), under the variant's temperature, fitted on the MIMIC-IV validation split and applied unchanged to all four cohorts. The other four variants are overlaid in gray behind it, and the shaded band represents the bootstrap interval for each decile’s gap. All panels share both axes, so the reference diagonal has the same slope in each, and equal vertical displacements carry the same meaning. Each panel is annotated with its mean calibration gap. Every cohort’s largest deviation occurs in the top decile: the internal and HiRID curves under-predict there, while MC-MED and eICU-CRD over-predict (Section 3.9).

**Table 9:** Calibration of the EHR-only model at the primary 30-day outcome, by decile of predicted risk, on the MIMIC-IV held-out set and the three external cohorts.

Cohort	Mean gap	First decile	Tenth decile	ECE (equal-count)
MIMIC-IV (internal)	−0.0050	+0.0029	−0.0303 [−0.0592, −0.0039]	0.0063
HiRID	−0.0184	−0.0042	−0.0506 [−0.0645, −0.0365]	0.0184
MC-MED	+0.0159	−0.0137	+0.1670 [+0.1054, +0.2293]	0.0352
eICU-CRD	+0.0187	−0.0288	+0.0916 [+0.0845, +0.0989]	0.0362

The mean calibration gap is the mean predicted probability minus the observed event rate. The gaps for the first and tenth deciles represent are the same quantity within those deciles, with the tenth carrying its bootstrap interval. The expected calibration error is computed over 10 equal-count bins, following the convention used in this section.

One temperature per variant and outcome window, fitted on the MIMIC-IV validation split, is applied unchanged to every cohort, including HiRID and MC-MED. The rescale is monotone, so discrimination is unaffected and only the calibration statistics change: The mean gap is −0.0184 on HiRID and +0.0159 on MC-MED, compared to −0.0242 and +0.0052 from the raw ensemble probability, and Table 6 carries both columns.

The mean calibration gap was small in all cohorts, but it was also the least informative calibration statistic (32). In every cohort, including the development cohort, the largest deviation occurred in the tenth decile of predicted risk, which is the range most relevant for alerting thresholds. Internally, the model under-predicted risk in this decile by −0.0303, which was six times larger than the mean gap. HiRID showed under-prediction across the full risk range, with the largest deviation also occurring at the top. MC-MED and eICU-CRD changed direction across the risk distribution and over-predicted in the highest decile by +0.1670 and +0.0916, respectively. Thus, at clinically relevant operating points, these cohorts were assigned higher risk than they actually carried. A mean gap of +0.0159 should therefore not be interpreted as evidence that the model is calibrated where it would be used. This pattern was not limited to the EHR-only model shown in Figure 8. In the two cohorts with top-decile over-prediction, the same pattern appeared across all variants and was strongest for the tri-modal model.

The temperature fitted in the development cohort did not transfer reliably. Using the same mean-logit aggregation, temperature scaling improved ECE on MIMIC-IV, from 0.0160 to 0.0063 and on HiRID from 0.0272 to 0.0184. It had little effect on MC-MED, changing ECE from 0.0367 to 0.0352, and worsened calibration on eICU-CRD from 0.0286 to 0.0362. The rescaled values are reported because they follow the stated procedure, but these results show that a temperature estimated at the development institution is not a safe default for deployment. Supplementary Results describe recalibration using receiving-site outcomes as an upper bound rather than a deployable method. A one-parameter temperature corrected HiRID but not MC-MED, while a two-parameter Platt map corrected MC-MED and overshot on eICU-CRD.

Two reporting conventions should be noted. Expected calibration error was computed using ten equal-width bins in Tables 4–6 and ten equal-count bins in this section and Figure 8, where the unit of analysis is the decile. The two estimates differ because predicted risks are skewed. In addition, MC-MED and HiRID do not provide death timestamps, so their 30-day labels are derived from length of stay and should be treated as approximate.

## 4 Discussion

This study quantified the incremental value of two non-tabular modalities, namely ECG machine measurements and clinical notes, beyond a strong structured EHR baseline for early ICU mortality prediction. The analysis used a controlled design in which model variants differed only by the set of input modalities.

First, on MIMIC-IV all five variants achieved nearly identical AUROC for the primary outcome, and every paired AUPRC difference against the EHR-only model included zero. Across the three outcome windows, one of twelve intervals excluded zero: the 48-h text comparison, which carried no AUROC difference and did not survive correction (Section 3.1). It is therefore exploratory rather than evidence of a robust modality effect, and a modality effect that is absent across windows should not be presented as a clinically meaningful benefit of multimodality.

Second, no defect was identified in the preprocessing checks performed on the ECG input matrix, so the null ECG result is not explained by any faults those checks would detect. The ECG modality is composed of clinically interpretable intervals, frontal-plane axes, an indicator for undetectable P waves, and a multi-hot encoding of the 100 most frequent printed diagnostic statements, with every model trained on that representation. The result bounds are narrower than the modality as a whole: They concern machine-derived measurements and printed statements read within the first 6 h of the ICU stay, and they carry no implications for raw 12-lead waveforms.

The model did read the rebuilt ECG channel. Within model D’s ECG branch, the leading attributed inputs were “sinus rhythm,” “sinus tachycardia,” “abnormal ECG,” heart rate, and QT interval. However, no ECG input received high weight relative to the structured record. Across branches, the first ECG input ranked 106th in the full held-out set and 13th among patients with an ECG. In all three explainer settings reported in Supplementary Results S-R5, the leading ECG input was a printed diagnostic statement rather than an interval or an axis. The first derived measurement ranked 597th, 129th, and 66th across these settings. Despite evidence that the ECG channel was being used, discrimination did not improve. The most plausible interpretation is redundancy. Rate, rhythm, and hemodynamic state are already documented in the structured EHR during the same 6-h window, so the 12-lead machine summary adds little information that the model does not already possess.

A related mechanism is that a clinical note synthesizes what the clinician has already seen, including the ECG, so the ECG adds little once text is available. This mechanism predicts that text should rank ahead of ECG. Head-to-head comparisons showed this pattern across all three windows, with intervals excluding zero in two of them. This result coexists with both modalities being indistinguishable from the EHR-only model. If the structured record already captures rate, rhythm, and hemodynamic state, then neither ECG nor text adds substantially to the baseline, while the modality reflecting clinical interpretation can still dominate the modality summarizing a tracing. However, this mechanism cannot be tested directly here because it concerns physician notes, and MIMIC-IV does not provide such notes within the 6-h prediction window. The text result should be interpreted with clear boundaries. After excluding discharge summaries, the only text available to a 6-h model in MIMIC-IV is the radiology report. This document describes an imaging study rather than an integrated clinical assessment and is available for fewer than one in five ICU stays. We therefore tested whether the limited value of text could reflect the absence of care-team narratives in MIMIC-IV rather than the 6-h prediction task itself.

This result contrasts with reports that clinical notes and ECGs carry prognostic signals in the ICU. Nursing and physician notes from the first 48 h predict a composite of prolonged stay and in-hospital death with AUCs of 0.826 and 0.796 separately, and 0.839 together (33). Notes from the first 24 h predict mortality at horizons from 48 h to 30 days with AUROC 0.87, falling to 0.82 (34). An ECG risk score learned from the MIMIC-IV electrocardiographic signal, combined with a severity score and age, reaches an AUC of 0.806 for 28-day mortality (35).

Five differences could account for the divergence, and we offer them as hypotheses rather than demonstrated mechanisms, because this study was not designed to separate them. First, the comparator differs: Those models predict from text alone or add an ECG score to a small set of severity variables, whereas the baseline here is a 1,587-feature structured EHR model with an AUROC of 0.8969, making the question asked incremental rather than absolute. Second, the observation window is 6 h compared to their 24–48, which affects both how much has been documented and how much of it has been filed. Third, the availability and nature of the text differ: Only 19.6% of stays here have a radiology report within the window, and a radiology report describes an imaging study rather than an integrated clinical assessment. Fourth, both added modalities may be redundant with the structured record: Rate, rhythm, hemodynamic state, and much of what a report would summarize are already charted within the same 6 h, so a model reading 1,587 structured columns may already contain that information. Fifth, the ECG modality here is a machine summary of intervals, axes, and printed statements, whereas a model learned from the waveform analyzes the signal itself (35, 36). None of the five is demonstrated by the experiments reported here.

The alternative explanation involving care-team narratives was examined in a supplementary MIMIC-III analysis, where nursing and physician notes are time-stamped and available within the same 6-h window for 20.8% and 24.5% of stays. That arm has been rebuilt under the rules of the main analysis: death within 30 days of ICU admission, adults, one stay per patient, total length of stay excluded, and chart observations admitted only if filed before the window closed. It now comprises 17,763 stays with 263 deaths in the held-out set and is reported in Supplementary S1 and Supplementary Figure S8. Neither note type materially changed discrimination. No arm changed AUROC by more than 0.0058 on the full test set. Among the fourteen paired comparisons, only one reached nominal significance: the C_rad arm, with ΔAUROC +0.0058 and *p* = 0.039, but it did not survive correction. Every paired AUPRC interval against the EHR-only model included zero, and five of the seven arms had negative point estimates. The largest AUPRC estimate was +0.0143 for the C_rad arm, with an interval from −0.0068 to 0.0355, compared with +0.0004 for radiology text in MIMIC-IV, with an interval from −0.0036 to 0.0044. Combining note types did not yield any benefit, and doubling the token budget did not change the result. Four limitations remain. MIMIC-III and MIMIC-IV originate from the same institution and cover overlapping calendar years, so this arm is not an independent institutional replication. MIMIC-III records no filing time for laboratory results or for radiology reports, so those two sources are admitted based on charting time alone. The arm has no ECG branch and therefore cannot address the asymmetry between ECG and text. Its power analysis showed that it could exclude only effects larger than about one AUPRC point, while the effects considered here range from 0.004 to 0.015. Read within these limits, the MIMIC-III arm provides supportive, hypothesis-generating sensitivity evidence rather than a definitive demonstration at the task level. It is consistent with the modest text effect being a property of the 6-h prediction window rather than the document type available in a specific database, and it cannot establish that. The companion comparison within MIMIC-IV, in which the apparent benefit of text diminishes as the admissibility definition is tightened, is shown for a fixed held-out test set of 14,232 stays in Supplementary Figure S9.

A further caution concerns what the structured model learns. Part of the signal comes from the care process rather than physiology. The count columns alone reached AUROC 0.7900, which was above SOFA and close to SAPS-II. Removing these columns reduced AUROC by 0.0109. Measurement frequency is institution-dependent and is the component of the signal that transfers least reliably.

A much weaker eICU-CRD result is obtained if the preprocessing rule differs between training and inference, resembling feature attrition and population shift. During training, missing values were imputed with raw zeros before standardization, while the external script imputed unmappable columns with the training mean. Since only 18.4% of the feature matrix is mappable from eICU-CRD, this input-distribution mismatch dominated the result. Applying the same imputation rule during training and external inference recovered about 0.13 AUROC and nearly all of the calibration error, as shown in Section 3.4. We report this failure mode because it is easy to introduce and difficult to detect. It raises no error, does not affect internal validation, and appears exactly like a genuine domain shift. Feature attrition remains real, and the 6–17 AUROC point drop relative to internal performance is a fair estimate of the cost of transfer without retraining. At a usable alert rate, the model identifies about one-third of deaths on eICU-CRD. Identifying 90% of deaths would require flagging three-quarters of the cohort. Although decision-curve analysis still shows a positive net benefit across a wide threshold range, the alert burden would prevent deployment. In the primary window on HiRID, the robustness-trained variant ranked fourth out of five, and the ordering was not stable under ensemble re-composition. Based on the present evidence, no model variant can be recommended over another for transfer.

The comparison with SOFA and SAPS-II confirms findings reported in prior studies using earlier MIMIC versions (5, 12). Machine-learning models substantially outperform these clinical scores for individual mortality prediction, and this advantage is not affected by whether the learned model uses only structured EHR data or a multimodal architecture. Therefore, SOFA and SAPS-II remain useful as cohort-level severity scores, but they should not be treated as substitutes for a learned patient-level predictor.

A more informative benchmark is the linear model trained on the same structured inputs as the EHR-only neural model. This comparison must account for the five-seed ensemble, as discussed in Section 3.2. Approximately half of the ensemble-level advantage comes from ensembling rather than from the architecture itself. Bagging does not improve the linear model because it is not variance-limited. The most accurate summary is that the five-seed neural ensemble outperforms the best linear fit by about 0.012 AUROC, with approximately 0.006 attributable to the architecture. This effect is real and worth reporting, but it should not be overstated. It also does not transfer uniformly. The deep model provides the greatest advantage where transfer is weakest and shows no reliable advantage where transfer is strongest, as shown in Section 3.4.

For teams developing an ICU early-warning system, the practical priorities follow from the effect sizes observed in this study. The first priority is the outcome definition, as it determines the meaning of every subsequent performance estimate. The second priority is ensuring preprocessing consistency between development and deployment. This was the largest isolated effect in the study, as reverting only the missing-value rule reduced AUROC by 0.1329 on eICU-CRD. The third priority is the exclusion of features that post-date the prediction time. The fourth is the model family, which contributed +0.0126 AUROC with an interval that excludes zero. Additional modalities should come last. Based on the current evidence, the two representations evaluated here—early radiology reports and machine-derived ECG measurements and statements read within a 6-h window—should not be expected to improve performance. This is significant because the text branch is computationally expensive. The frozen BiomedBERT component contains 110,171,264 parameters, occupies 440.7 MB in fp32, and represents 99.35% of the model. In contrast, the EHR encoder, ECG encoder, fusion block, and prediction head together occupy only 2.9 MB. A deployment using notes must also support tokenization, transformer inference for each patient, text availability logic, and free-text governance. Comparing checkpoints alone understates this cost because the architecture instantiates and runs the text encoder even for variants without text input.

The fairness audit also indicates that demographic subgroups require explicit calibration. Performance gaps in clinical algorithms are well documented and can lead to unequal care (37, 38). In this study, the subgroup aged 80 years or older had the highest event rate, at 17.1%, and the lowest model AUROC, at 0.853. This is the group in which decision-support errors would be especially costly. Calibration drift in racial subgroups was bounded and occurred against broadly stable discrimination; however, it would still justify subgroup-specific temperature rescaling before deployment.

The proposed pipeline differs from prior work in three main respects, as summarized in Supplementary Table S6. First, the controlled modality ablation across five variants isolates the marginal value of ECG and text more directly than previous benchmarks and yields a bounded null result rather than an apparent benefit. Second, the trained models are evaluated on three independent ICU databases without retraining. Because none of the three provides ECG measurements or clinical notes, that evaluation pertains the structured EHR backbone with both extra modalities masked. Third, the integration of SHAP analysis, subgroup audit, calibration assessment, and robustness analysis provides the necessary auditability layers for clinical deployment.

## 5 Limitations

Several limitations should be considered when interpreting these findings. The first concerns the definition of the text modality. Free-text documentation in MIMIC-IV is limited to discharge summaries and radiology reports. Nursing notes, physician notes, admission notes, and progress notes available in MIMIC-III have no direct counterpart in MIMIC-IV. Because discharge summaries are written after the outcome is known, they cannot be used for early prediction. Under the strict 6-h availability rule, the only usable text source is the radiology report, which is available for 19.6% of stays. Therefore, the estimated value of clinical text in MIMIC-IV specifically refers to early radiology reports. The supplementary MIMIC-III analysis tests whether richer clinical notes might change this conclusion. It has been rebuilt under the timing and outcome rules of the main analysis, with the exception noted in Supplementary S1 that MIMIC-III records no filing time for laboratory results or radiology reports.

The ECG modality also has a restricted definition. It is represented by machine-derived measurements and printed diagnostic statements rather than by full 12-lead waveforms. A waveform-based encoder could extract information that these summaries overlook, especially in deaths related to arrhythmia. The absence of an ECG effect in this study therefore applies to machine-generated ECG summaries and should not be generalized to raw waveform modeling.

The prediction window was fixed at the first 6 h after ICU admission. Longer windows and event-time formulations remain outside the scope of this analysis. Deaths after hospital discharge were not counted. In addition, event times in HiRID and MC-MED were approximate because neither database publishes a death timestamp. For those cohorts, outcome windows were derived from length of stay.

External validation was limited by the structure of the available datasets. All three external cohorts are ICU registries, and no prospective deployment was attempted. Their validation results are also constrained by feature-schema differences rather than by a controlled distribution shift. In eICU-CRD, only 18.4% of the feature matrix could be reconstructed. The external results should therefore be read as estimates of what can be recovered without retraining, not as transportability under comparable data collection. Two eICU-CRD laboratory variables were initially mapped to incorrect MIMIC-IV items and were dropped rather than used. The mapping was corrected, and the whole external validation was re-scored accordingly, with the correction changing AUROC by −0.0011 and every eICU-CRD value reported here reflecting the corrected one.

The stability of external model rankings is another limitation. We bounded the effect by re-forming each ensemble from subsets of its own seeds rather than by training additional independent ensembles. This showed that most between-variant differences in the external cohorts did not survive ensemble re-composition. Independent ensembles for each variant would be needed to resolve the remaining uncertainty. Model selection was also statistically weak given the available validation-split size: The runner-up epoch was within one bootstrap standard error of the selected epoch in all 75 models of the main grid and in 84 of the 85 models of the main analysis (Section 3.1). The consequence is limited to about 0.001 AUROC, and the reported models are five-seed ensembles that average over that noise, but a larger validation split or selection by cross-validated AUROC would make the choice more stable.

The explanation analysis has its own constraints. At the gradient-sample budget used here, a per-patient explanation reconstructs the model's departure from its background mean with approximately one third of that quantity as estimation noise. For this reason, only averages over thousands of patients and five seeds are reported, and no individual explanation is presented. Attribution also identifies what the model uses, not what contains information in an absolute sense. The ECG channel was read by the model but did not improve discrimination, and SHAP rankings alone could not have predicted that combination of findings.

Finally, several preprocessing and coding decisions may affect generalization. Imputing features unavailable in an external database is a modeling choice with material consequences. This study used training-mean imputation and documented how strongly a different rule can distort external performance. Race and ethnicity coding in MIMIC-IV is coarse and partly missing, which limits the resolution of the fairness audit. Other preprocessing choices, including plausibility bounds, top-N item caps, the 100-statement ECG vocabulary, and the 256-token text budget, were fixed using sensitivity-blind defaults.

A further limitation concerns the validation pipeline itself. Preparation of this analysis uncovered several defects in it, each of which changed a reported number: a train-to-inference imputation mismatch worth 0.1329 AUROC on eICU-CRD, temperature scaling that was not applied in two external cohorts, two external laboratory variables mapped to the wrong source identifiers, and features that post-dated the prediction time. Each issue was found by checking an artifact rather than by the analysis failure, and none raised an error. Leakage and preprocessing faults of this kind are a documented source of irreproducible machine-learning results across the sciences (39). We cannot exclude the possibility that further defects of the same kind remain, and a null result is more vulnerable to them than a positive one because a pipeline fault that degrades a modality channel is indistinguishable from that modality carrying no information. This is the reason the ECG and text channels are checked directly in Sections 3.3 and 3.6 rather than inferred from the null, and the reason the analysis code and the result tables are released.

## 6 Conclusion

This study asked whether ECG machine measurements and clinical notes add prognostic information to a structured EHR model for early ICU mortality prediction, using a 6-h feature window after ICU admission and fixed outcome windows. In the held-out MIMIC-IV cohort of 9,806 first ICU stays of adult patients, no multimodal variant outperformed the EHR-only baseline for the primary 30-day outcome, and every paired AUPRC difference against that baseline included zero. The same was true in the landmark cohort alive at sixth hour, in the subset that met a 24-h minimum-stay criterion, among the patients who actually had an ECG within the window, and among those who actually had a radiology report. Across 12 modality contrasts over three outcome windows, one AUPRC interval excluded zero, for text at 48 h, but it did not survive correction. No defect was identified in the preprocessing checks performed on the ECG and text inputs, and the null result is not explained by the model failing to attend to those channels: Among patients with an ECG, the model ranked its leading ECG input thirteenth of 1,827 features, and prediction still did not improve. The most likely reading is that these signals were redundant with structured variables charted in the same window, too rarely present within it, or too weak to register at the cohort level. By contrast, model architecture contributed a measurable gain. L2-penalized logistic regression on the same features and split reached AUROC 0.8844, compared with 0.8969 for the neural ensemble. At a matched number of fits, the architecture added 0.0126 AUROC, with an interval from 0.0083 to 0.0168. No added modality produced anything comparable, so the gains in this setting came from nonlinear modeling and ensembling rather than from multimodal fusion.

Transferred without retraining, the models reached AUROC 0.833 on HiRID, 0.774 on MC-MED, and 0.7268 on eICU-CRD. These are not validations of the multimodal model. None of the three cohorts supplies ECG measurements or clinical notes at inference, so every variant runs on the structured backbone with both extra modalities masked, and differences between variants cannot be attributed to the modalities themselves. Neither HiRID nor MC-MED publishes a death timestamp, so their 30-day outcomes are derived from length of stay, in HiRID from the last recorded observation and in MC-MED from hospital length of stay anchored at emergency-department arrival, and are therefore approximate. Read within those bounds, the models retained useful discrimination in all three databases while transportability varied substantially across health systems. Mean calibration gaps were small but concealed over-prediction reaching 0.167 in the highest-risk decile, which is the range where an alerting threshold would sit: Acceptable average calibration does not guarantee reliable risk estimates for the patients most likely to be acted on. ICU prediction studies are highly sensitive to feature timing, cohort construction, and preprocessing consistency, and apparent external gains or failures can arise from any of the three rather than from genuine differences in generalization.

The findings are limited by what was measured. The text modality consists of the early radiology report, the only free-text document in MIMIC-IV available within a 6-h window, and is present for 19.6% of stays. The ECG modality includes machine-derived measurements and printed diagnostic statements, rather than raw 12-lead waveforms. The prediction window encompasses the first 6 h of the ICU stay. The paired intervals serve as confidence limits instead of pre-specified equivalence margins, and the largest compatible AUPRC gain, 0.0068, is on the same order as the study’s own single-seed training noise, so effects smaller than that cannot be resolved here. Nothing in these results excludes raw waveform modeling, higher-frequency physiologic signals, care-team narratives, richer temporal note representations, or task-specific multimodal pretraining. What they demonstrate is that the ECG and text representations available in this context did not enhance early ICU mortality prediction beyond a structured EHR baseline under strict temporal constraints and external validation.

For the development of an ICU early-warning system, the outcome definition, exclusion of post-window features, and consistency of preprocessing between development and deployment were more significant than the addition of a second or third modality. The practical implication is that future work should prioritize temporally valid study design, transparent handling of missingness, calibration in target populations, and prospective evaluation before increasing model complexity. Multimodal learning remains promising, but its value should be validated against robust EHR-only baselines, with paired uncertainty estimates and external validation, rather than assumed based solely on the availability of additional data streams.

## Data Availability

Publicly available datasets were analyzed in this study. This data can be found here: All four datasets used in this study are publicly available through PhysioNet under credentialed access: MIMIC-IV v3.1: https://physionet.org/content/mimiciv/3.1/, eICU-CRD v2.0: https://physionet.org/content/eicu-crd/2.0/, MC-MED: https://physionet.org/content/mc-med/, HiRID v1.1.1: https://physionet.org/content/hirid/1.1.1/. Access requires researcher credentialing and a signed Data Use Agreement. All preprocessing, feature-engineering, training, external-validation, SHAP, fairness, and statistical-analysis scripts, together with the external feature-mapping dictionaries (one machine-readable file per external cohort) and the result tables from which every reported number is drawn, are publicly available at https://github.com/AikoIs/for_article. The scripts read the credentialed source databases, the derived data directory, and the trained checkpoints, none of which may be redistributed, so the pipeline is auditable but not runnable without independent access to the four databases.

## References

[1] Vincent J-L Moreno R Takala J Willatts S De Mendonça A Bruining H. The SOFA (sepsis-related organ failure assessment) score to describe organ dysfunction/failure. Intensive Care Med. (1996) 22(7):707–10. 10.1007/BF01709751 8844239

[2] Le Gall JR Lemeshow S Saulnier F. A new simplified acute physiology score (SAPS II) based on a European/north American multicenter study. JAMA. (1993) 270(24):2957–63. 10.1001/jama.1993.03510240069035 8254858

[3] Johnson AEW Bulgarelli L Shen L Gayles A Shammout A Horng S. MIMIC-IV, a freely accessible electronic health record dataset. Sci Data. (2023) 10:1 10.1038/s41597-022-01899-x 36596836 PMC9810617

[4] Pang K Li L Ouyang W Liu X Tang Y. Establishment of ICU mortality risk prediction models with machine learning algorithm using MIMIC-IV database. Diagnostics. (2022) 12(5):1068. 10.3390/diagnostics12051068 35626224 PMC9139972

[5] Yang S Cao L Zhou Y Hu C. A retrospective cohort study: predicting 90-day mortality for ICU trauma patients with a machine learning algorithm using XGBoost using MIMIC-III database. J Multidiscip Healthc. (2023) 16:2625–40. 10.2147/JMDH.S416943 37701177 PMC10493110

[6] Harutyunyan H Khachatrian H Kale DC Ver Steeg G Galstyan A. Multitask learning and benchmarking with clinical time series data. Sci Data. (2019) 6(1):96. 10.1038/s41597-019-0103-9 31209213 PMC6572845

[7] Purushotham S Meng C Che Z Liu Y. Benchmarking deep learning models on large healthcare datasets. J Biomed Inform. (2018) 83:112–34. 10.1016/j.jbi.2018.04.007 29879470

[8] Chen T Guestrin C. XGBoost: a scalable tree boosting system. In: Krishnapuram B Shah M Smola AJ Aggarwal CC Shen D Rastogi R, editors. Proceedings of the 22nd ACM SIGKDD International Conference on Knowledge Discovery and Data Mining. New York, NY: ACM (Association for Computing Machinery). (2016). p. 785–94. 10.1145/2939672.2939785

[9] Ke G Meng Q Finley T Wang T Chen W Ma W. LightGBM: a highly efficient gradient boosting decision tree. In: Guyon I Von Luxburg U Bengio S Wallach H Fergus R Vishwanathan S Garnett R, editors. Advances in Neural Information Processing Systems 30. Red Hook, NY: Curran Associates, Inc. (2017). p. 3146–54.

[10] Acosta JN Falcone GJ Rajpurkar P Topol EJ. Multimodal biomedical AI. Nat Med. (2022) 28(9):1773–84. 10.1038/s41591-022-01981-2 36109635

[11] Soenksen LR Ma Y Zeng C Boussioux L Villalobos Carballo K Na L. Integrated multimodal artificial intelligence framework for healthcare applications. npj Digit Med. (2022) 5:149. 10.1038/s41746-022-00689-4 36127417 PMC9489871

[12] Pirracchio R Petersen ML Carone M Rigon MR Chevret S Van Der Laan MJ. Mortality prediction in intensive care units with the super ICU learner algorithm (SICULA): a population-based study. Lancet Respir Med. (2015) 3(1):42–52. 10.1016/S2213-2600(14)70239-5 25466337 PMC4321691

[13] Awad A Bader-El-Den M McNicholas J Briggs J. Early hospital mortality prediction of intensive care unit patients using an ensemble learning approach. Int J Med Inf. (2017) 108:185–95. 10.1016/j.ijmedinf.2017.10.002 29132626

[14] Ismukhamedova A Uvaliyeva I Rakhmetullina Z. Experimental study of a medical data analysis model based on comparative performance of classification algorithms. Indones. J Electr. Eng. Comput. Sci. (2024) 36(1):672–84. 10.11591/ijeecs.v36.i1.pp672-684

[15] Ismukhamedova A Uvaliyeva I Belginova S. Integrating machine learning in electronic health passport based on WHO study and healthcare resources. Inform. Med. Unlocked. (2024) 44:101428. 10.1016/j.imu.2023.101428

[16] Ismukhamedova AM Bakanova AP. Designing the architecture of a medical decision-making system and medical data analysis based on machine learning technologies. In: Ivanovskaya II Posnova MV, editors. Development of Modern Technologies: Theoretical and Practical Aspects. Proceedings of the 5th International Scientific and Practical Conference. Petrozavodsk: MCNP “Novaya Nauka”. (2024). p. 58–72. 10.46916/16102024-2-978-5-00215-552-1

[17] Pollard TJ Johnson AEW Raffa JD Celi LA Mark RG Badawi O. The eICU collaborative research database, a freely available multi-center database for critical care research. Sci Data. (2018) 5(1):180178. 10.1038/sdata.2018.178 30204154 PMC6132188

[18] Kansal A Chen E Jin T Rajpurkar P Kim D. Multimodal clinical monitoring in the emergency department (MC-MED) (version 1.0.1). PhysioNet. (2025). 10.13026/wvyw-g663

[19] Faltys M Zimmermann M Lyu X Hüser M Hyland S Rätsch G. HiRID, a high time-resolution ICU dataset. PhysioNet v1.1.1. (2021). 10.13026/nkwc-js72

[20] Hyland SL Faltys M Hüser M Lyu X Gumbsch T Esteban C. Early prediction of circulatory failure in the intensive care unit using machine learning. Nat Med. (2020) 26(3):364–73. 10.1038/s41591-020-0789-4 32152583

[21] Steyerberg EW Harrell FE. Prediction models need appropriate internal, internal-external, and external validation. J Clin Epidemiol. (2016) 69:245–7. 10.1016/j.jclinepi.2015.04.005 25981519 PMC5578404

[22] Gow B Pollard T Nathanson LA Johnson A Moody B Fernandes C. MIMIC-IV-ECG: diagnostic electrocardiogram matched subset. PhysioNet. (2023). 10.13026/4nqg-sb35

[23] Gu Y Tinn R Cheng H Lucas M Usuyama N Liu X. Domain-specific language model pretraining for biomedical natural language processing. ACM Trans. Comput. Healthc. (2021) 3(1):2 (1–23). 10.1145/3458754

[24] Hayat N Geras KJ Shamout FE. Medfuse: multi-modal fusion with clinical time-series data and chest x-ray images. Proc. Machine Learn. Res. (2022) 182:479–503.

[25] Lambden S Laterre PF Levy MM Francois B. The SOFA score — development, utility and challenges of accurate assessment in clinical trials. Critical Care. (2019) 23(1):374. 10.1186/s13054-019-2663-7 31775846 PMC6880479

[26] Collins GS Moons KGM Dhiman P Riley RD Beam AL Van Calster B. TRIPOD + AI statement: updated guidance for reporting clinical prediction models that use regression or machine learning methods. Br Med J. (2024) 385:e078378. 10.1136/bmj-2023-078378 PMC11019967 38626948

[27] Efron B Tibshirani RJ. An Introduction to the Bootstrap. New York, NY: Chapman & Hall (1993).

[28] DeLong ER DeLong DM Clarke-Pearson DL. Comparing the areas under two or more correlated receiver operating characteristic curves: a nonparametric approach. Biometrics. (1988) 44(3):837–45. 10.2307/2531595 3203132

[29] Lundberg SM Lee SI. A unified approach to interpreting model predictions. In: Guyon I Von Luxburg U Bengio S Wallach H Fergus R Vishwanathan S Garnett R, editors. Advances in Neural Information Processing Systems 30. Red Hook, NY: Curran Associates, Inc. (2017). p. 4765–74.

[30] Futoma J Simons M Panch T Doshi-Velez F Celi LA. The myth of generalisability in clinical research and machine learning in health care. Lancet Digital Health. (2020) 2(9):e489–92. 10.1016/S2589-7500(20)30186-2 32864600 PMC7444947

[31] Vickers AJ Elkin EB. Decision curve analysis: a novel method for evaluating prediction models. Med Decis Making. (2006) 26(6):565–74. 10.1177/0272989X06295361 17099194 PMC2577036

[32] Van Calster B McLernon DJ Van Smeden M Wynants L Steyerberg EW. Calibration: the achilles heel of predictive analytics. BMC Med. (2019) 17:230. 10.1186/s12916-019-1466-7 31842878 PMC6912996

[33] Huang K Gray TF Romero-Brufau S Tulsky JA Lindvall C. Using nursing notes to improve clinical outcome prediction in intensive care patients: a retrospective cohort study. J Am Med Inform Assoc. (2021) 28(8):1660–6. 10.1093/jamia/ocab051 33880557 PMC8324216

[34] Mahbub M Srinivasan S Danciu I Peluso A Begoli E Tamang S. Unstructured clinical notes within the 24 h since admission predict short, mid & long-term mortality in adult ICU patients. PLoS One. (2022) 17(1):e0262182. 10.1371/journal.pone.0262182 34990485 PMC8735614

[35] Wang Q Wang B Chen B Li Q Zhao Y Dong T. Interpretable machine learning model integrating electrocardiographic and acute physiology metrics for mortality prediction in critical ill patients. J Clin Med. (2025) 14(20):7163. 10.3390/jcm14207163 41156033 PMC12565335

[36] Raghunath S Ulloa Cerna AE Jing L Vanmaanen DP Stough J Hartzel DN. Prediction of mortality from 12-lead electrocardiogram voltage data using a deep neural network. Nat Med. (2020) 26(6):886–91. 10.1038/s41591-020-0870-z 32393799

[37] Obermeyer Z Powers B Vogeli C Mullainathan S. Dissecting racial bias in an algorithm used to manage the health of populations. Science. (2019) 366(6464):447–53. 10.1126/science.aax2342 31649194

[38] Seyyed-Kalantari L Zhang H McDermott MBA Chen IY Ghassemi M. Underdiagnosis bias of artificial intelligence algorithms applied to chest radiographs in under-served patient populations. Nat Med. (2021) 27(12):2176–82. 10.1038/s41591-021-01595-0 34893776 PMC8674135

[39] Kapoor S Narayanan A. Leakage and the reproducibility crisis in machine-learning-based science. Patterns. (2023) 4(9):100804. 10.1016/j.patter.2023.100804 37720327 PMC10499856

