# Supplementary material for: Early radiology reports and ECG machine measurements do not measurably improve 30-day ICU mortality prediction from the first 6 h of the stay: a multimodal ablation with external validation of the structured EHR backbone
Source: Front Med Technol. 2026 Oct 28;8:1902843. doi: 10.3389/fmedt.2026.1902843 (PMC13752055; doi:10.3389/fmedt.2026.1902843)
Supplement: Supplementary file 1 [file Datasheet1.pdf]

Supplementary Material

Early radiology reports and ECG machine measurements do not measurably improve 30-day ICU mortality prediction from the first six hours of the stay: a multimodal ablation with external validation of the structured EHR backbone

This file contains the supplementary methods, results, tables, and figures referenced in the main text. This material is reported here rather than in the article so that the article remains at a readable length.

Supplementary Methods

S-M1. Fidelity of the SHAP attributions

Two checks were run on the attributions of model D, on 2,000 class-stratified held-out patients against a background of 500 training patients, for each of the five seeds. They are reported together because they fail independently: the first tests whether the attributions reconstruct the model's output, the second whether the features they name are the ones the network uses.

Completeness. Expected gradients satisfy an additivity identity a patient's attributions sum to that patient's predicted probability minus the mean prediction over the background — exactly only in the limit of infinite gradient samples. The summed attributions track that difference at Pearson r = 0.9463–0.9664 across the five seeds of model D and 0.9563–0.9702 for model A, leaving a mean absolute residual of 0.2824–0.3478 and 0.2717–0.3279 of the quantity being reconstructed.

That residual is estimation noise rather than a systematic gap, and we established it rather than asserting it. Across *independent* explainer draws the residual falls from 0.4197–0.4383 at 25 gradient samples to 0.1533–0.1619 at 200 — the decline of a Monte-Carlo estimate. The ranking is stable throughout: Spearman $\rho \geq 0.9977$ against the largest budget, with 18 to 19 of the top 20 features unchanged at the budget used in the main analysis and 20 of 20 at the largest. The replicates are drawn independently, since budgets sharing a random seed are correlated and make the overlap look uniformly perfect. The budget sweep uses a 400-patient subsample of the same patients, since its cost scales with the sample, so it bounds the estimator's behavior rather than the ranking's sampling error over patients.

The practical consequence is a limit on use rather than on the analysis: a *per-patient* explanation at this budget carries about a third of its magnitude as noise. We therefore present no individual explanation, and every ranking in Section 3.3 is a mean over 2,000 patients and five seeds, where that noise largely cancels.

Deletion. This check does not use the identity. Replacing the *k* most strongly attributed features by their training means, which is what an unmeasured value already is in the scaled matrix, moves the predicted probability 38.6 times as far as replacing *k* features drawn at random from those the patient actually has a value for. At k = 100 the ratio is 6.45. The control matters: drawing uniformly over all 1,827 columns inflates the ratio about threefold, because 57% of the EHR block and about half of the full input are already zero, so most such draws delete nothing. The test covers every attributed input, including the text block and the two availability indicators, so it speaks to the claims made about them.

Wrapper verification. Before any attribution is used, the explained wrapper's per-patient predictions are checked against the evaluation pipeline's. The mean absolute difference is below 10^{-6} on all five seeds, i.e. 0.000000 to the six decimals the result files carry. Two explainer setups exist in

this work and only this one is fidelity-checked: the per-branch wrapper used for the within-branch orderings of **Figure 3** and **Supplementary Figure S1** masks the other modalities out and, for model D, holds the ECG availability mask at one for every patient, so it answers "what would this model attend to if every patient had an ECG and none had a note". No statement about where an ECG feature stands *against* the structured record is drawn from it.

`The explainer draws from numpy's global generator; the script seeds it`, so the shipped attributions are reproducible.

Supplementary Results

S-R1. Landmark sensitivity analysis (patients alive and at risk at hour six)

The primary analysis imposes no minimum length of stay, so 369 stays (0.6% of the cohort) die at or before the sixth hour: 16.3% of all 48-hour events, and 55 of the 339 in the held-out set. A strict landmark design would exclude them. The landmark analysis reported here re-scores the models already fitted on the full cohort; it is not a retraining in a survivor cohort. We do not adopt it as the primary analysis, because excluding them conditions on surviving the window, which is the error a minimum-stay criterion makes, but the choice is arguable and the analysis is therefore reported.

Re-scoring the same models on the 9,751 held-out stays that survive past hour six:

Window	EHR-only AUROC, all stays	alive at hour 6	Events
48 h	0.9371	0.9280	339 → 284
7 d	0.9106	0.9038	681 → 626
30 d (primary)	0.8969	0.8917	1,042 → 987

Discrimination falls, as it must, since the excluded patients are the most predictable events. On the landmark cohort **none of the twelve modality contrasts has an interval excluding zero at any window**, and we are careful about what that does and does not mean. It does *not* mean the null strengthened. The one baseline comparison whose interval excludes zero on the full cohort, text at 48 hours, moves from +0.0088 [+0.0007, +0.0174] to **+0.0111 [−0.0016, +0.0227]**: the point estimate rises by a quarter, and the bootstrap standard error rises from 0.0043 to 0.0062 because 16% of the events are removed, so the interval widens around a slightly larger effect rather than shrinking toward zero. What the analysis establishes is that the null is not an artifact of retaining patients who were already dead when the prediction was due; it does not resolve the 48-hour text signal in either direction; the retrained landmark analysis that does is reported in Section 3.1 and in Supplementary Table S9.

One limit of the design belongs here. This is a re-scoring of the held-out set, not a retraining: 314 of the 369 stays fall in the training and validation splits and remain in the fitted models. It therefore bounds the effect of retaining those patients *at evaluation*, not at fitting.

S-R2. Ensemble re-composition on the external cohorts

Each variant is represented by a single five-seed ensemble, so a rival account of the between-variant spread on the external cohorts is that an ensemble is itself a noisy draw. Bounding that required no retraining, since per-seed external inference is a forward pass over checkpoints already on disk. Each variant's ensemble was re-formed from three of its five seeds, 100 compositions per variant, on HiRID — the cohort carrying the largest between-variant differences.

Three results follow.

Ensemble composition moves a variant about as far as the variants differ from each other. At the primary horizon the spread across the five variants is 0.0310, while re-forming one variant from three

of its five seeds moves it by up to 0.0323; the within-variant range is the larger. At 48 hours the figures are 0.0465 against 0.0380.

The internal yardstick is the wrong one, by roughly a factor of eight. The standard deviation of AUROC across the five individual seeds of one ensemble is at most 0.0040 internally but 0.0223 on HiRID. Ensembling stabilizes a model far less under domain shift than on held-out data from the development institution.

Most of the significant differences do not survive re-composition. Of the 20 between-variant comparisons on HiRID, only **4 keep their sign** when both ensembles are re-formed from three of their five seeds. The bare count overstates the instability, and the distribution belongs beside it: of the 16 that flip, five flip in at most 4 draws of 100 and five in more than 30. It is the latter five that carry the conclusion. The p-values are not in doubt at these sample sizes — they are essentially readouts of n, but they test whether two particular ensembles differ, which is not the question.

One directional statement survives: variant B, trained with ECG, is weaker than variant D on HiRID at 48 hours under every one of the 100 re-compositions, though the magnitude is not estimable, ranging from +0.0022 to +0.0610. The analysis was run on HiRID alone; the 27 of 30 eICU-CRD comparisons that survive Bonferroni have not been subjected to it, and since eICU-CRD is four times larger, its intervals are narrower rather than its estimates more stable, so we do not interpret those differences either.

S-R3. What recalibration would take

The diagnostics below are fitted on each receiving cohort's **own outcomes**. They are therefore an upper bound on what recalibration could achieve, not a method available at deployment, since those labels are exactly what transfer without retraining assumes are unavailable, and no recalibrated performance is reported anywhere in this work as if they were available.

A one-parameter temperature takes HiRID's expected calibration error from 0.0184 to 0.0058. It does nothing for MC-MED under either fitting criterion (0.0352 to 0.0355 by log loss, to 0.0348 fitted to the reported statistic). On eICU-CRD the answer depends entirely on what the temperature is fitted to: by log loss, the usual objective, it makes matters slightly worse (0.0362 to 0.0372), while a temperature fitted to the statistic we report achieves 0.0274. Reporting the log-loss arm alone would support the conclusion that one parameter cannot help there, which is an artifact of fitting one criterion and reporting another.

Adding an intercept, a two-parameter Platt map, takes MC-MED to 0.0125 and eICU-CRD to 0.0196. It removes MC-MED's top-decile over-prediction entirely (+0.1670 to −0.0064) and overshoots on eICU-CRD, turning +0.0916 of over-prediction into −0.0512 of under-prediction, larger in magnitude than the internal deviation Section 3.9 flags and in the band that matters. The practical reading is that MC-MED needs an intercept, eICU-CRD needs a slope chosen for the right criterion, and neither is available without labeled data from the receiving site.

Two further conventions belong here. The deciles of Section 3.9 and **Figure 8** are equal-count bins; with MC-MED's 2,575 patients the tenth holds 258, which is why that gap carries the widest interval in the table. And **Figure 8 draws variant A in color** with the other four variants overlaid in gray, so the spread is visible but not separable by variant; the per-variant values are reported in the result files accompanying this work.

S-R4. Per-variant calibration in the top decile

The top-decile deviation reported in Section 3.9 is not an artifact of plotting the EHR-only model. The tenth-decile gap runs from +0.1670 (variant A) to +0.2207 (variant D) on MC-MED and from +0.0916 (A) to +0.1321 (D) on eICU-CRD; on HiRID it is smallest for D_robust (−0.0117) and largest for A (−0.0506), with D third at −0.0226; D is the best calibrated there by expected calibration error rather than by this statistic. Variant A is therefore representative of the direction but not of the magnitude, and the tri-modal model is the worst calibrated where it matters on both cohorts that over-predict.

S-R5. The ECG attribution under three explainer backgrounds

Section 3.3 reports that the availability indicator for ECG outranks every individual ECG measurement over the full held-out set, and declines to read that as the model attending to ordering behavior rather than to tracings. The reason is that the two are not separable by this estimator: a stay without an ECG carries a constant imputed vector, so the indicator and the 110 measurement columns move together whenever the mask is varied, and expected gradients divide the credit according to what the attribution contrasts against. Three settings bracket the range, all on model D over the same 1,827 inputs, and age is the highest-ranked input in all three. Over the full held-out set against a pooled background the ECG availability indicator ranks 16, the first ECG input (the printed statement “sinus rhythm”) 106 and the first derived measurement (heart rate) 597. Restricted to patients who have an ECG, against the training split at large where 21.6% of stays have one, the indicator takes rank 2, the first ECG input 13 and the first derived measurement 129. Against a background matched on ECG availability, where the mask is held fixed on both sides of the contrast, the indicator falls to rank 1,753 and contributes essentially nothing, as it must, while the first ECG input rises to rank 2 behind age alone and the first derived measurement to 66. The dilution the pooled background introduces is therefore real and measurable, and the matched background removes it. Two statements are invariant across all three and are the ones Section 3.3 and Section 4 rely on: the model reads the ECG channel, and no ECG input approaches the top of the ranking once availability is allowed to vary, which is the situation any deployment faces. In every setting the leading ECG input is a printed diagnostic statement rather than an interval or an axis.

S-R6. The precision of the null, and what it is not

A null is only as informative as its precision, so the bound is reported rather than the absence of significance. At the primary 30-day horizon the largest AUPRC improvement compatible with the five contrasts that add a modality is +0.0068 and the largest compatible AUROC improvement +0.0019. Taken over all ten model pairs the corresponding upper limits are +0.0090 and +0.0025, and both belong to the text-against-ECG contrast rather than to a modality added to the baseline.

Two limits on that statement belong with it. These are confidence limits read off paired comparisons, not pre-specified equivalence margins, so they describe what the data are compatible with and never show that an effect is excluded. And they condition on the five fitted ensembles: the single-seed AUROC standard deviation at this horizon is 0.0015 to 0.0033, the same size as the AUROC limit itself, so this study cannot resolve a modality effect smaller than its own training noise. The AUPRC limit is therefore the informative one, and Section 3.1 quotes it.

S-R7. Epoch selection and how much it can matter

Across the 75 models of the main grid the selected epoch ranged from 1 to 4 with a median of 2, no model reached the 20-epoch budget, and validation AUROC fell from its peak before the patience-5 criterion expired (Supplementary Table S2, Supplementary Figure S2). The selection is nonetheless statistically weak. The bootstrap standard error of validation AUROC on the 9,804-patient validation

split is 0.0056 while the median gap between the best and the second-best epoch is 0.0013, so in all 75 runs the runner-up lies within one standard error of the epoch that was kept.

The quantity at stake, about 0.001 AUROC, is larger than the modality differences this paper draws a conclusion from, and it is not dismissed as negligible. What makes the reported comparisons resolvable in spite of it is that they are paired on identical patients and pooled over five seeds, which cancels the component of the noise the variants share; epoch selection, by contrast, compares two draws of the same quantity on one validation split.

Supplementary Tables

Supplementary Table S1. The linear baseline carried to the external cohorts. The L2 logistic regression fitted on the MIMIC-IV training split is applied without refitting to the same aligned feature matrices and the same horizon labels the neural models are scored against. `Delta` is the EHR-only model minus the linear model, so a negative value means the linear model is ahead. One linear fit is compared against a five-seed ensemble, which favors the neural side by about +0.006 AUROC internally.

Cohort	Window	N	Events	Linear AUROC	Linear AUPRC	Linear Brier	Linear calibration gap	EHR-only model AUROC	Delta
HiRID	48 h	33,666	1,204	0.8254	0.3578	0.0277	-0.0075	0.8540	+0.0286
HiRID	7 d	33,666	1,831	0.8227	0.3802	0.0414	-0.0035	0.8115	-0.0112
HiRID	30 d	33,666	2,062	0.8214	0.3816	0.0476	+0.0211	0.8333	+0.0119
MC-MED	48 h	2,575	106	0.7847	0.2224	0.0362	-0.0088	0.7835	-0.0012
MC-MED	7 d	2,575	174	0.7628	0.2506	0.0578	-0.0116	0.7801	+0.0173
MC-MED	30 d	2,575	258	0.7615	0.3112	0.0812	-0.0008	0.7744	+0.0129
eICU-CRD	48 h	138,868	5,055	0.7252	0.2738	0.0305	-0.0071	0.7597	+0.0345
eICU-CRD	7 d	138,868	9,480	0.6810	0.2613	0.0579	-0.0174	0.7120	+0.0310
eICU-CRD	30 d	138,868	12,837	0.6880	0.2834	0.0763	-0.0098	0.7268	+0.0388

Supplementary Table S2. Training behavior under the revised protocol (selection on validation AUROC, 20-epoch budget, patience 5). Every model's per-epoch curve is available; Supplementary Figure S2 plots the main grid.

Arm	Models	Median selected epoch	Max selected epoch	Median epochs run	Reached the 20-epoch budget	Median AUROC fall from peak
30-day horizon (primary)	25	2	4	7	0	0.0230
30-day, published-text sensitivity arm	10	2	3	7	0	0.0233
48-hour horizon	25	2	4	7	0	0.0242
7-day horizon	25	2	3	7	0	0.0296

Supplementary Table S3. Feature ablations on the L2 logistic-regression baseline, primary 30-day outcome, same split throughout. The count columns record how many times an item was charted in the window; `los is the total length of stay, which is not a predictor.`

Feature set	Columns	AUROC	AUPRC	ΔAUROC vs all [95% CI]
all features	1,587	0.8844	0.5459	reference
values only (counts removed)	1,270	0.8735	0.5180	-0.0109 [−0.0157, −0.0062]
counts only (measurement frequency)	317	0.7900	0.3422	-0.0944 [−0.1065, −0.0841]
all features + los	1,588	0.8827	0.5504	-0.0017 [−0.0026, −0.0009]

Supplementary Table S4. Discrimination of the tri-modal model by time from ICU admission to death, holding the controls fixed: the 8,723 held-out stays that survived to hospital discharge. Using the 8,764 stays without a 30-day event as controls instead would count the 41 in-hospital deaths beyond day 30 as survivors and leave that band with no positives, so those deaths are reported as their own row. Bands are scored against "died in this band," not against the 30-day label. The 48-hour band holds 337 of the 339 48-hour events: two stays carry a recorded death time at or before ICU admission (−10.3 h and −0.1 h) and fall in no band.

Events restricted to	Events	AUROC	AUPRC	Mean predicted risk of the events
death within 48 h	337	0.9517 [0.9409, 0.9609]	0.6314	0.5604
death 2–7 d	342	0.8931 [0.8783, 0.9073]	0.2888	0.3410
death 7–30 d	361	0.8523 [0.8355, 0.8685]	0.1994	0.2629
death after 30 d	41	0.8382 [0.7853, 0.8868]	0.0249	0.2432

Supplementary Table S5. Operating characteristics at the primary 30-day horizon, EHR-only model, temperature-scaled probabilities. The screening posture sets the threshold to identify 90% or 80% of the deaths; the staffing posture sets it to a fixed alert budget.

Cohort	Posture	Threshold	Alerts per 100	Sensitivity	Specificity	PPV	NPV	Number needed to evaluate
MIMIC-IV (internal)	sensitivity ≈ 90%	0.0613	36.6	0.900	0.698	0.262	0.983	3.8
MIMIC-IV (internal)	sensitivity ≈ 80%	0.1246	24.4	0.800	0.822	0.349	0.972	2.9
MIMIC-IV (internal)	alert budget 5%	0.4888	5.0	0.329	0.983	0.700	0.925	1.4
MIMIC-IV (internal)	alert budget 10%	0.3206	10.0	0.520	0.950	0.552	0.943	1.8
HiRID	sensitivity ≈ 90%	0.0116	51.1	0.900	0.514	0.108	0.987	9.3
HiRID	sensitivity ≈ 80%	0.0228	33.3	0.800	0.697	0.147	0.982	6.8
HiRID	alert budget 5%	0.1834	5.0	0.337	0.969	0.412	0.957	2.4
HiRID	alert budget 10%	0.1051	10.0	0.490	0.925	0.300	0.965	3.3
MC-MED	sensitivity ≈ 90%	0.0300	62.6	0.903	0.405	0.145	0.974	6.9
MC-MED	sensitivity ≈ 80%	0.0557	47.5	0.802	0.562	0.169	0.962	5.9
MC-MED	alert budget 5%	0.5096	5.0	0.233	0.970	0.465	0.919	2.1
MC-MED	alert budget 10%	0.3150	10.0	0.368	0.930	0.368	0.930	2.7
eICU-CRD	sensitivity ≈ 90%	0.0210	75.5	0.900	0.260	0.110	0.962	9.1
eICU-CRD	sensitivity ≈ 80%	0.0566	56.0	0.800	0.464	0.132	0.958	7.6
eICU-CRD	alert budget 5%	0.3593	5.0	0.228	0.968	0.421	0.925	2.4
eICU-CRD	alert budget 10%	0.2617	10.0	0.344	0.925	0.318	0.933	3.1

Supplementary Table S6. Comparison with prior MIMIC-based ICU mortality work.

Field	This study (2026)	Yang et al. (2023)	Harutyunyan et al. (2019)	Purushotham et al. (2018)	Pirracchio et al. (2015)

Dataset	MIMIC-IV v3.1 + eICU-CRD + MC-MED + HiRID	MIMIC-III v1.4	MIMIC-III v1.4	MIMIC-III v1.4	MIMIC-II v2.6
N (development)	9,806 (test)	3,745	33,798	35,348	17,152
Modalities	EHR + ECG + Text	EHR only	EHR time series	EHR	EHR
Fusion	Modality attention	n/a	n/a	n/a	n/a
Internal AUROC	0.8969	0.885–0.946	≈0.871	0.866–0.94	0.85–0.94
External validation	**3 independent ICU cohorts, structured backbone with both extra modalities masked**	No	No	No	1 (French ICU, n=200)
Clinical-score benchmark	SOFA + SAPS-II (paired DeLong)	No	No	No	No
Interpretability	SHAP + attention	SHAP	Attention	Feature importance	Variable importance
Fairness audit	Yes (race/age/sex/ICU type)	No	No	No	No

Supplementary Table S7. Paired modality contrasts among the patients who actually hold each modality, at all three outcome windows. Each row compares one variant against the EHR-only model on the same patients: paired DeLong on AUROC, and a paired stratified bootstrap of 1,000 resamples on AUPRC. Subsets are defined by real availability inside the six-hour window. Intervals are nominal and enter no corrected family; where correction is applied, it is Bonferroni across the ten model pairs within a cohort and window, as in Section 2.4. At the primary 30-day window all twelve intervals contain zero.

Window	Subset	n	Events	Pair	ΔAUROC	DeLong p	ΔAUPRC [95% CI]
48 h	ECG available	2,130	68	A vs B	-0.0002	0.939	-0.0144 [−0.0454, 0.0129]
48 h	ECG available	2,130	68	A vs C	-0.0016	0.561	-0.0111 [−0.0305, 0.0075]
48 h	ECG available	2,130	68	A vs D	-0.0081	0.132	-0.0217 [−0.0587, 0.0120]
48 h	ECG available	2,130	68	A vs D_robust	-0.0103	0.053	-0.0344 [−0.0727, −0.0013]
48 h	text available	1,904	72	A vs B	-0.0062	0.015	-0.0268 [−0.0533, 0.0001]

48 h	text available	1,904	72	A vs C	-0.0010	0.710	-0.0006 [−0.0223, 0.0294]
48 h	text available	1,904	72	A vs D	-0.0053	0.139	-0.0007 [−0.0232, 0.0310]
48 h	text available	1,904	72	A vs D_robust	-0.0073	0.020	-0.0136 [−0.0337, 0.0140]
48 h	both available	783	29	A vs B	+0.0005	0.837	-0.0032 [−0.0321, 0.0242]
48 h	both available	783	29	A vs C	-0.0023	0.537	+0.0050 [−0.0182, 0.0315]
48 h	both available	783	29	A vs D	-0.0043	0.355	-0.0001 [−0.0332, 0.0348]
48 h	both available	783	29	A vs D_robust	-0.0070	0.109	-0.0066 [−0.0369, 0.0253]
7 d	ECG available	2,130	146	A vs B	-0.0007	0.806	-0.0059 [−0.0262, 0.0140]
7 d	ECG available	2,130	146	A vs C	+0.0022	0.205	-0.0085 [−0.0214, 0.0025]
7 d	ECG available	2,130	146	A vs D	+0.0018	0.507	-0.0136 [−0.0346, 0.0096]
7 d	ECG available	2,130	146	A vs D_robust	-0.0009	0.726	-0.0156 [−0.0399, 0.0103]

7 d	text available	1,904	139	A vs B	+0.0004	0.860	-0.0029 [−0.0199, 0.0170]
7 d	text available	1,904	139	A vs C	-0.0005	0.795	+0.0092 [−0.0057, 0.0257]
7 d	text available	1,904	139	A vs D	+0.0005	0.842	+0.0148 [−0.0055, 0.0360]
7 d	text available	1,904	139	A vs D_robust	-0.0003	0.917	+0.0201 [0.0020, 0.0415]
7 d	both available	783	51	A vs B	-0.0013	0.712	-0.0008 [−0.0385, 0.0358]
7 d	both available	783	51	A vs C	+0.0018	0.410	-0.0015 [−0.0156, 0.0126]
7 d	both available	783	51	A vs D	+0.0007	0.812	+0.0014 [−0.0320, 0.0288]
7 d	both available	783	51	A vs D_robust	-0.0006	0.840	+0.0120 [−0.0216, 0.0441]
30 d	ECG available	2,130	197	A vs B	-0.0003	0.877	-0.0005 [−0.0179, 0.0161]
30 d	ECG available	2,130	197	A vs C	+0.0001	0.954	+0.0032 [−0.0058, 0.0129]
30 d	ECG available	2,130	197	A vs D	+0.0010	0.631	-0.0003 [−0.0167, 0.0180]

30 d	ECG available	2,130	197	A vs D_robust	+0.0018	0.399	-0.0019 [−0.0197, 0.0156]
30 d	text available	1,904	208	A vs B	+0.0008	0.700	-0.0035 [−0.0159, 0.0081]
30 d	text available	1,904	208	A vs C	+0.0009	0.488	+0.0009 [−0.0092, 0.0120]
30 d	text available	1,904	208	A vs D	-0.0000	0.999	+0.0051 [−0.0046, 0.0157]
30 d	text available	1,904	208	A vs D_robust	+0.0021	0.238	+0.0034 [−0.0104, 0.0163]
30 d	both available	783	66	A vs B	-0.0025	0.434	-0.0006 [−0.0242, 0.0240]
30 d	both available	783	66	A vs C	+0.0024	0.163	+0.0058 [−0.0105, 0.0235]
30 d	both available	783	66	A vs D	-0.0008	0.710	+0.0063 [−0.0154, 0.0290]
30 d	both available	783	66	A vs D_robust	+0.0011	0.694	-0.0003 [−0.0240, 0.0253]

Supplementary Table S8. Sensitivity of the external results to the definition of the outcome window. HiRID and MC-MED publish no death timestamp, so their event times are derived from length-of-stay information; eICU-CRD publishes a discharge offset in minutes and its event time is exact, so it is included as a control. Labels are rebuilt from a perturbed event time while the predictions are held fixed. On HiRID the recorded time is the last observation of a patient who died and is therefore a lower bound, so only positive shifts are meaningful. The MC-MED row marked per patient re-anchors the event time at the end of the emergency-department stay, which the database records for each patient. The 7-day window and the wider shifts are reported in the released result tables.

Cohort	Window	Event-time shift	Events	Stays changing label	AUROC	ΔAUROC
HiRID	48 h	reported labels	1,204	0	0.8540	+0.0000
HiRID	48 h	+6 h	1,123	81	0.8602	+0.0062
HiRID	48 h	+24 h	750	454	0.8827	+0.0287
HiRID	48 h	+48 h	0	1,204	no event remains	—
HiRID	30 d	reported labels	2,062	0	0.8333	+0.0000
HiRID	30 d	+6 h	2,062	0	0.8333	+0.0000
HiRID	30 d	+24 h	2,062	0	0.8333	+0.0000
HiRID	30 d	+48 h	2,062	0	0.8333	+0.0000
MC-MED	48 h	-48 h	134	28	0.7603	-0.0232
MC-MED	48 h	-24 h	117	11	0.7711	-0.0124
MC-MED	48 h	-6 h	106	0	0.7835	+0.0000
MC-MED	48 h	reported labels	106	0	0.7835	+0.0000
MC-MED	48 h	+6 h	73	33	0.8175	+0.0340
MC-MED	48 h	+24 h	73	33	0.8175	+0.0340
MC-MED	48 h	+48 h	18	88	0.8587	+0.0751
MC-MED	48 h	per patient	106	0	0.7835	+0.0000
MC-MED	30 d	-48 h	258	0	0.7744	+0.0000

MC-MED	30 d	-24 h	258	0	0.7744	+0.0000
MC-MED	30 d	-6 h	258	0	0.7744	+0.0000
MC-MED	30 d	reported labels	258	0	0.7744	+0.0000
MC-MED	30 d	+6 h	258	0	0.7744	+0.0000
MC-MED	30 d	+24 h	258	0	0.7744	+0.0000
MC-MED	30 d	+48 h	257	1	0.7734	-0.0010
MC-MED	30 d	per patient	258	0	0.7744	+0.0000
eICU-CRD	48 h	-48 h	7,416	2,361	0.7134	-0.0463
eICU-CRD	48 h	-24 h	6,378	1,323	0.7320	-0.0277
eICU-CRD	48 h	-6 h	5,430	375	0.7498	-0.0098
eICU-CRD	48 h	reported labels	5,055	0	0.7597	+0.0000
eICU-CRD	48 h	+6 h	4,625	430	0.7733	+0.0136
eICU-CRD	48 h	+24 h	3,138	1,917	0.8115	+0.0518
eICU-CRD	48 h	+48 h	11	5,044	0.5143	-0.2454
eICU-CRD	30 d	-48 h	12,871	34	0.7266	-0.0002
eICU-CRD	30 d	-24 h	12,857	20	0.7266	-0.0002
eICU-CRD	30 d	-6 h	12,843	6	0.7267	-0.0000
eICU-CRD	30 d	reported labels	12,837	0	0.7268	+0.0000
eICU-CRD	30 d	+6 h	12,833	4	0.7268	+0.0000

eICU-CRD	30 d	+24 h	12,811	26	0.7271	+0.0003
eICU-CRD	30 d	+48 h	12,790	47	0.7274	+0.0006

Supplementary Table S9. The landmark analysis with the models refitted rather than re-scored. Every stay whose recorded death falls at or before the sixth hour was removed from all three splits, leaving 64,997 stays of which 9,751 are held out, and all five variants were retrained with five seeds at the 30-day and 48-hour horizons, 50 models in total; the 7-day horizon was not retrained. The outcome window is still measured from ICU admission, not from the landmark, so the estimates remain comparable with the main analysis. Each row is the paired difference against the EHR-only model on identical patients, with a stratified bootstrap of 1,000 resamples for AUPRC and the paired DeLong test for AUROC, as in Section 2.4. The re-scored column repeats the corresponding value from the analysis that kept the models fitted on the full cohort. The EHR-only baseline reached AUROC 0.8897 at 30 days (987 events) and 0.9302 at 48 hours (284 events), against 0.8917 and 0.9280 under re-scoring. No interval in this table excludes zero.

Window	Contrast	n	Events	Refitted ΔAUPRC [95% CI]	Refitted ΔAUROC	DeLong p	Re-scored ΔAUPRC [95% CI]
30 d	A to B	9,751	987	-0.0010 [−0.0054, 0.0032]	-0.0002	0.781	-0.0013 [−0.0073, +0.0043]
30 d	A to C	9,751	987	-0.0017 [−0.0068, 0.0032]	-0.0003	0.717	+0.0002 [−0.0044, +0.0049]
30 d	A to D	9,751	987	-0.0010 [−0.0062, 0.0042]	-0.0003	0.706	-0.0014 [−0.0074, +0.0048]
30 d	A to D_robust	9,751	987	+0.0019 [−0.0025, 0.0063]	+0.0006	0.330	-0.0010 [−0.0071, +0.0049]
48 h	A to B	9,751	284	+0.0036 [−0.0082, 0.0164]	-0.0030	0.087	-0.0042 [−0.0142, +0.0047]
48 h	A to C	9,751	284	-0.0058 [−0.0221, 0.0103]	-0.0030	0.024	+0.0111 [−0.0016, +0.0227]

48 h	A to D	9,751	284	-0.0052 [−0.0221, 0.0112]	-0.0019	0.244	+0.0080 [−0.0042, +0.0192]
48 h	A to D_robust	9,751	284	+0.0041 [−0.0135, 0.0200]	-0.0033	0.064	+0.0032 [−0.0088, +0.0150]

Supplementary Figures

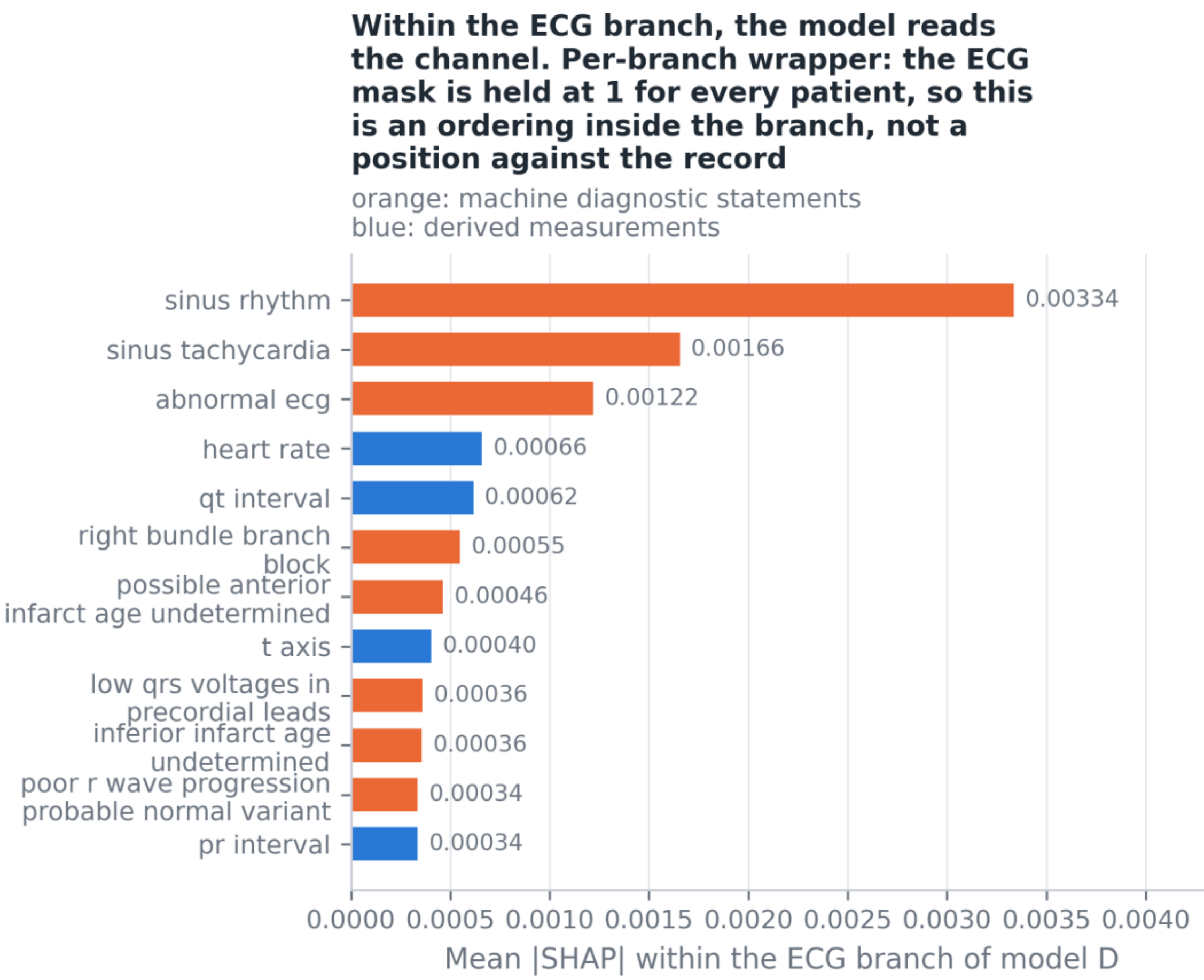

Supplementary Figure S1. SHAP attribution within the ECG branch of model D after the feature rebuild, computed with the per-branch wrapper that holds the ECG mask at one for every patient, an idealized-availability view that shows the ordering *inside* the branch and is not the model that produced Table 5 (Section 3.3), separating the machine's diagnostic statements from the derived measurements. It cannot show where those features stand against the structured record; **Figure 6** does that. The model reads the channel; it does not predict better for it (Section 3.1).

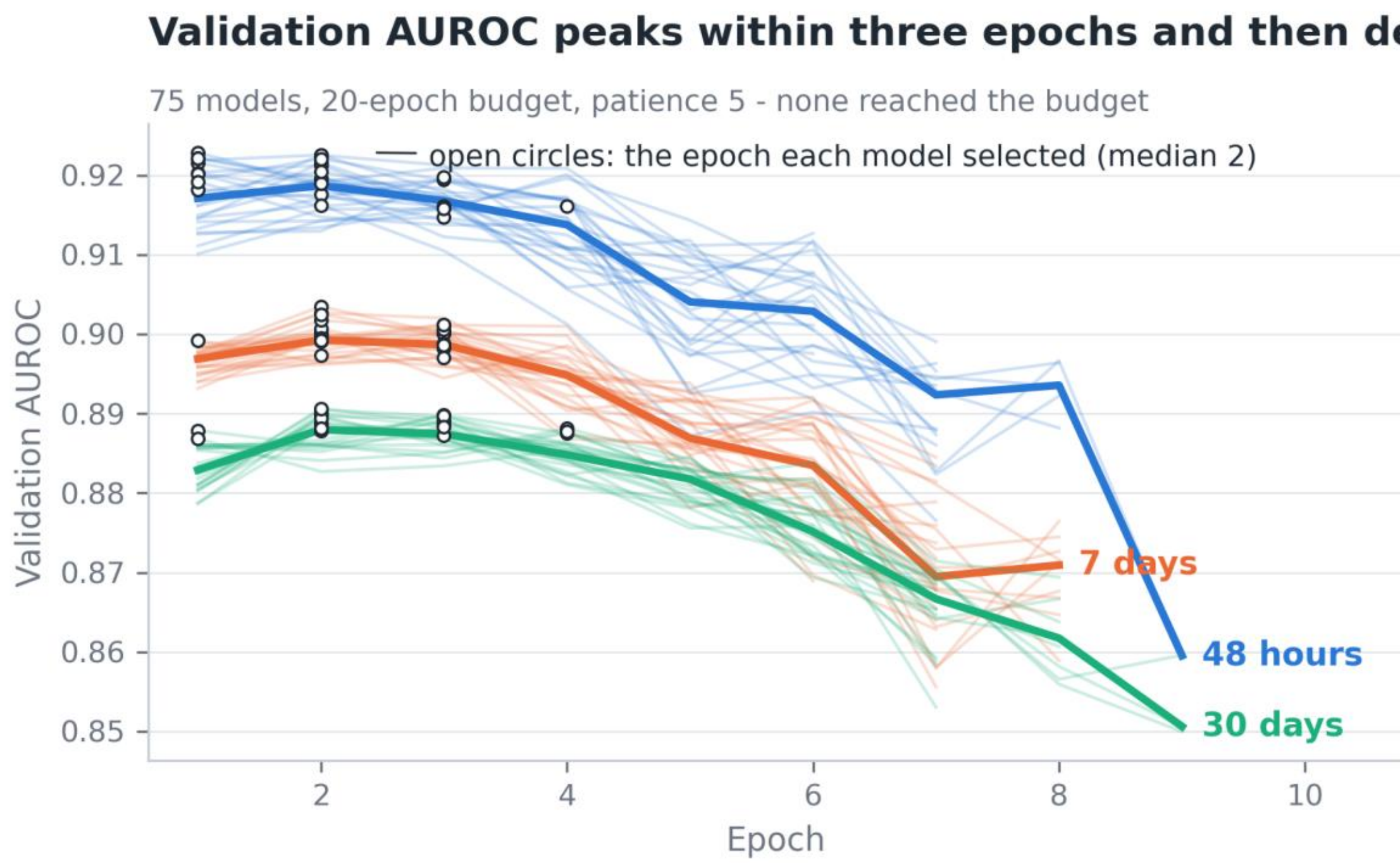

Supplementary Figure S2. Validation AUROC against epoch for the 75 models of the main grid, with the epoch each model selected marked. The curves peak within the first three epochs and then decline, and no model reaches the 20-epoch budget; the selection is nonetheless within noise (Section 3.1). The 10-model sensitivity arm is excluded from the plot but included in Supplementary Table S2.

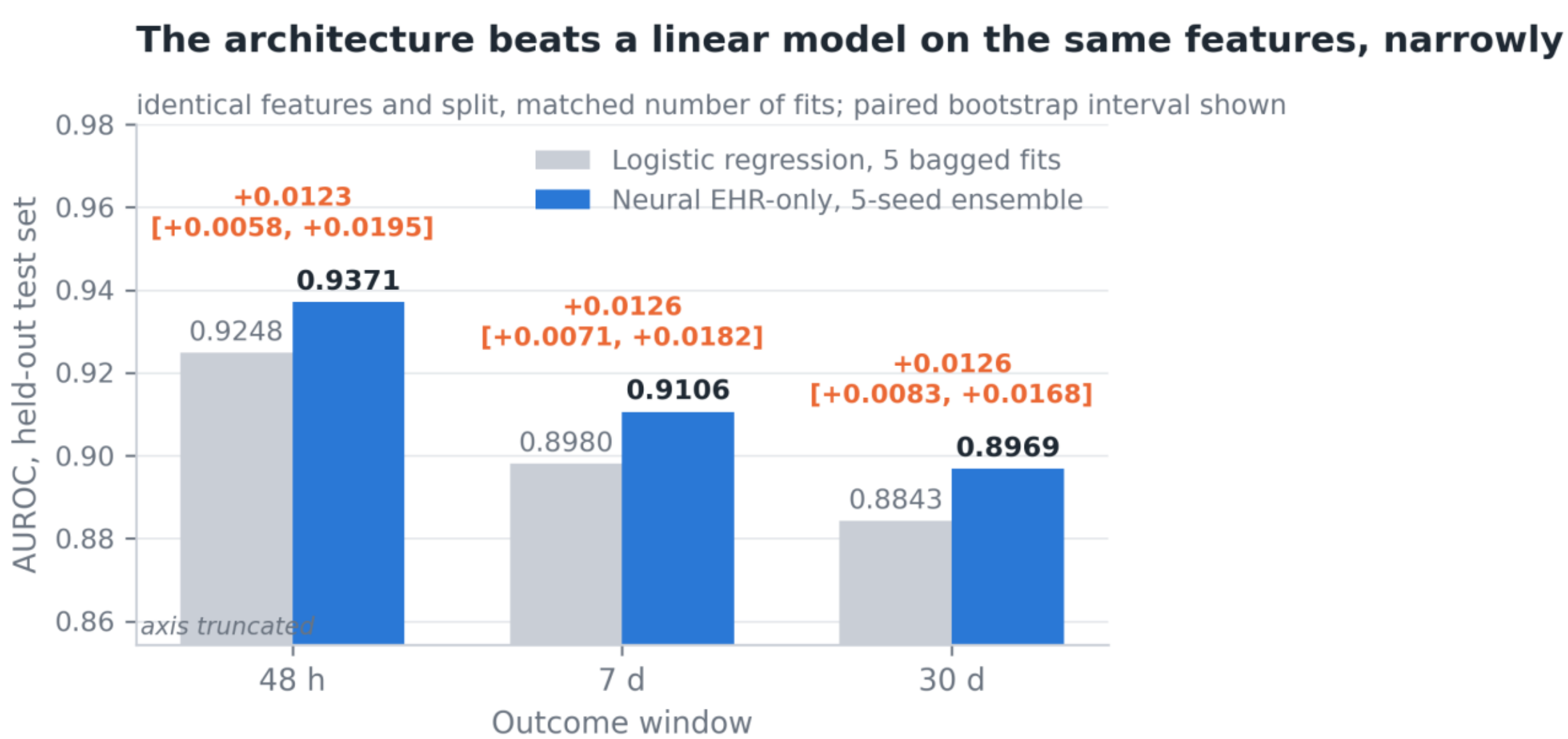

Supplementary Figure S3. The neural EHR-only model against L2 logistic regression on the identical feature matrix and split, at all three outcome windows.

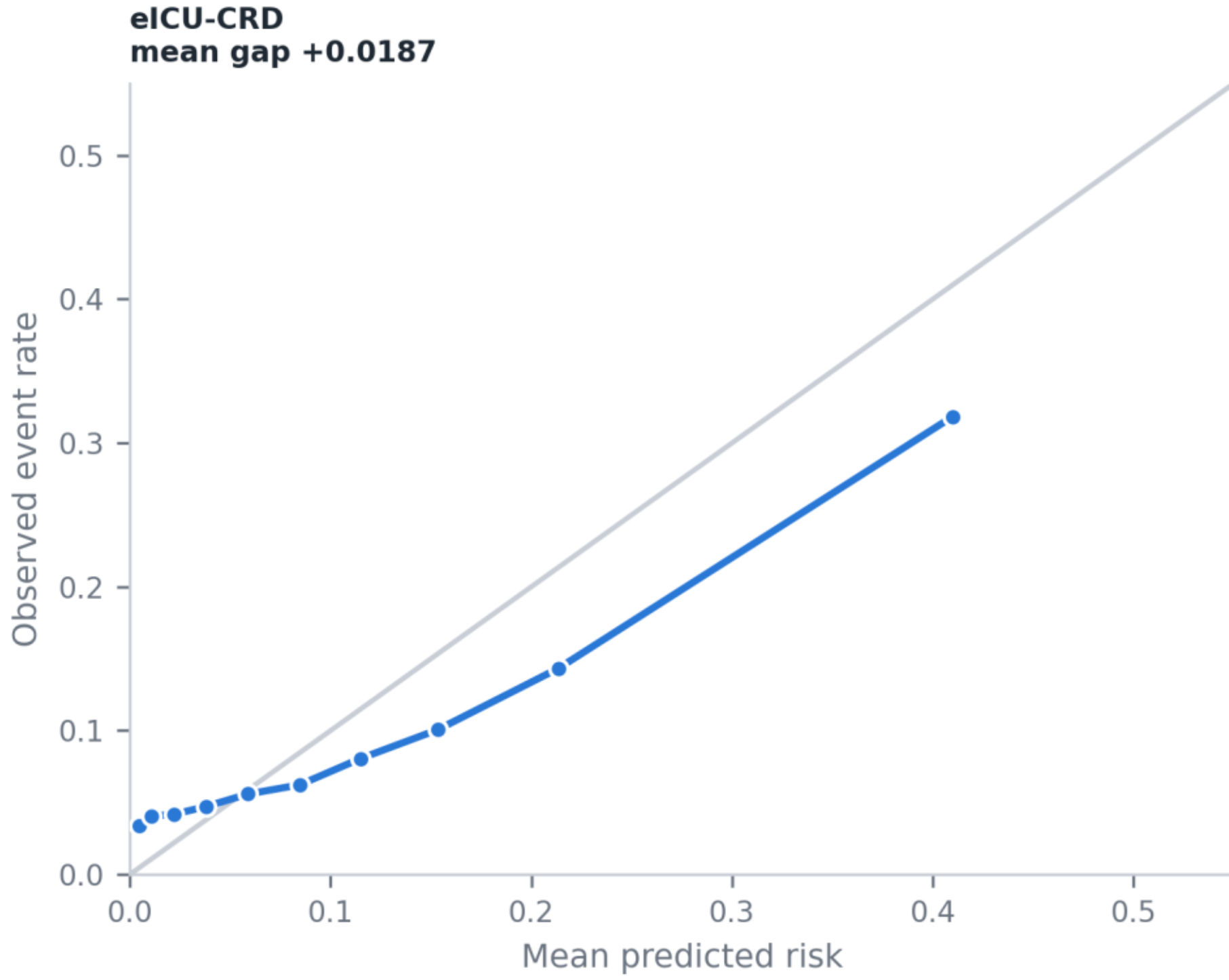

Supplementary Figure S4. Reliability diagram for the EHR-only model on eICU-CRD, the largest external cohort, by decile of predicted risk. After aligning the missing-value rule between training and external inference the mean calibration gap is +0.0187. The mean conceals a gradient: by decile the gap runs from −0.0288 in the lowest to +0.0916 in the highest, so the model under-predicts in the low-risk band and over-predicts in the high-risk band where the alerting thresholds of Section 3.8 sit. Discrimination on all cohorts and windows is given in Table 6; the other three cohorts are in **Figure 8**. This panel is drawn without the per-decile bootstrap band that Figure 8 carries; the intervals for every decile of every cohort are in the released result tables.

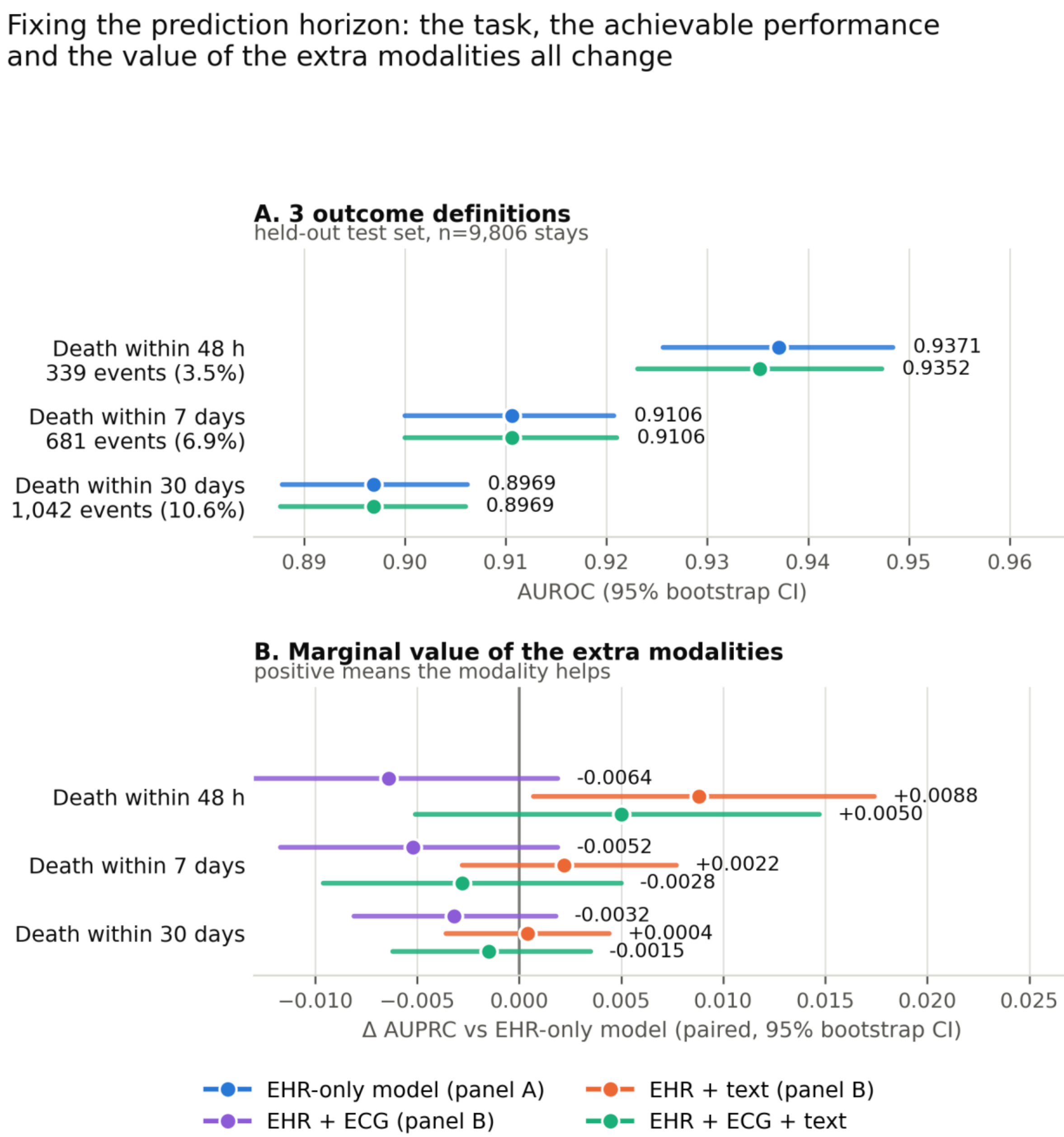

Supplementary Figure S5. Effect of the outcome window. Panel a: discrimination of the EHR-only model under each outcome definition, with the number of events; the same model and features score AUROC 0.9371 against a 48-hour outcome and 0.8969 against a 30-day one. Panel b: the marginal value of the text and ECG branches under each definition. The text arm at 48 hours is positive with its interval clear of zero (+0.0088 [+0.0007, +0.0174]); every other contrast against the EHR-only model contains zero at every window.

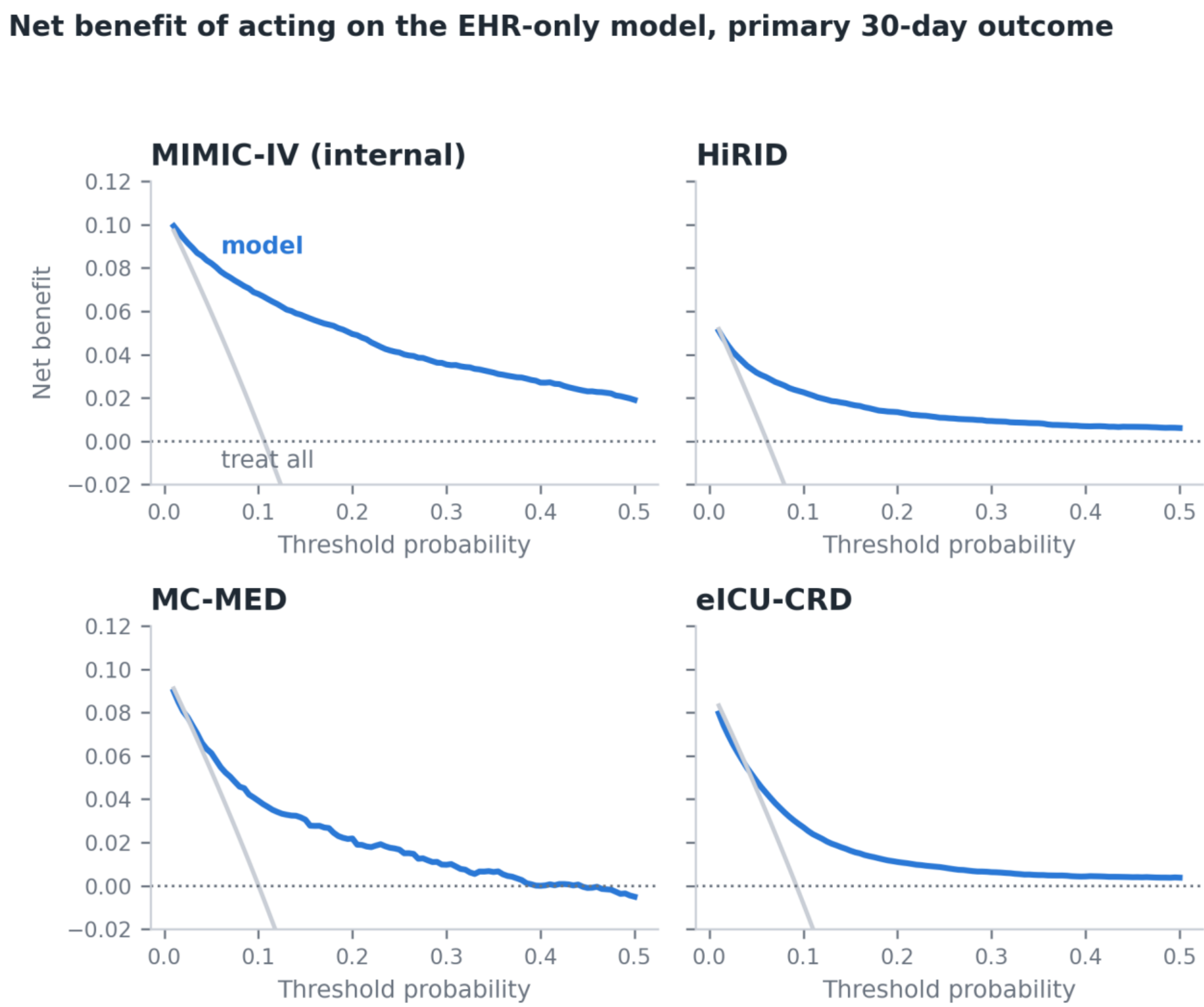

Supplementary Figure S6. Decision-curve analysis at the primary 30-day horizon: net benefit of acting on the EHR-only model (blue) against treating every patient (gray) and treating none (the zero line), across threshold probabilities. The model exceeds both defaults over the range quoted in Section 3.8 on all four cohorts.

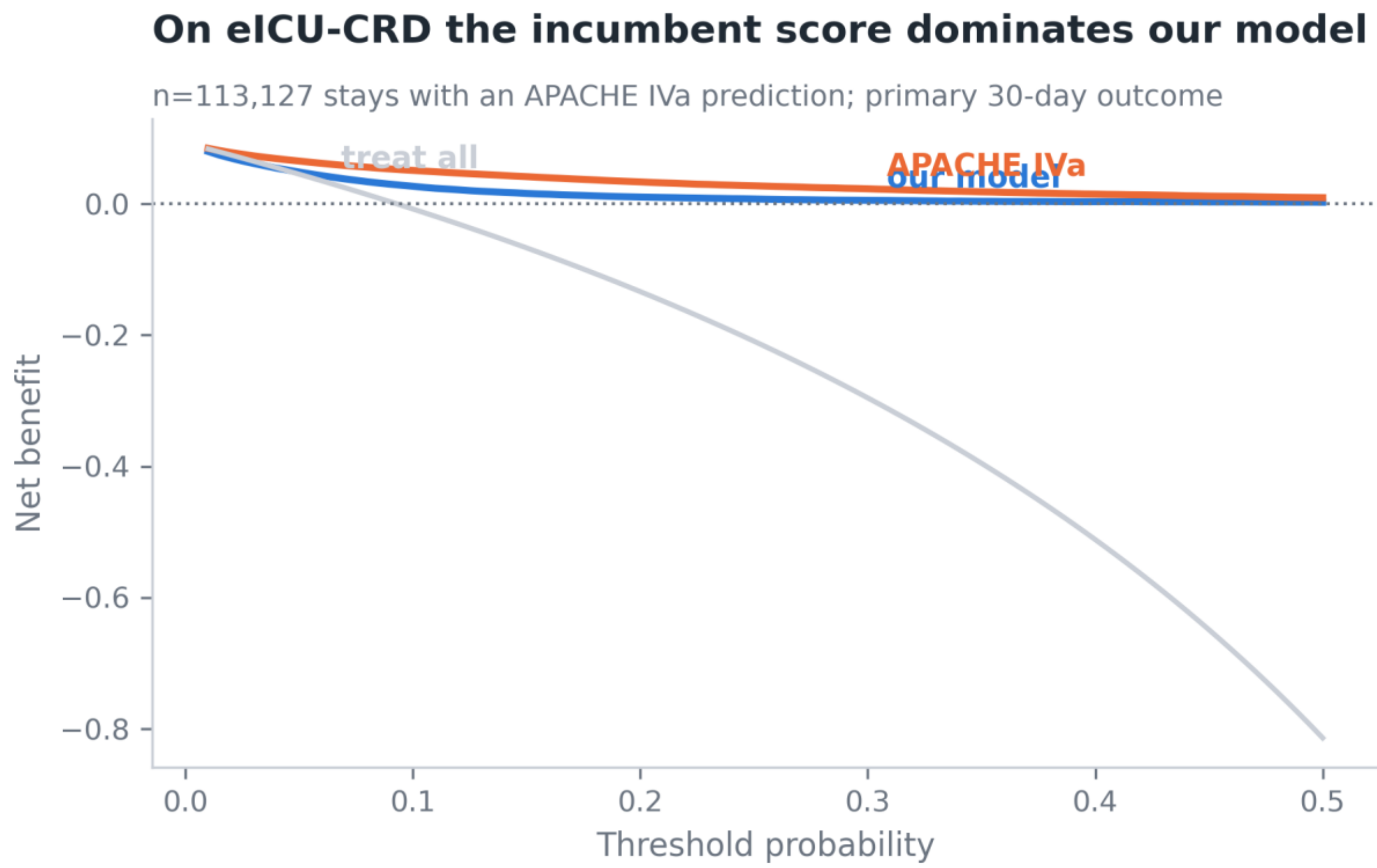

Supplementary Figure S7. Net benefit on eICU-CRD of the EHR-only model against APACHE IVa, the score the database already provides, on the 113,127 stays carrying an APACHE prediction. The incumbent dominates at every threshold examined.

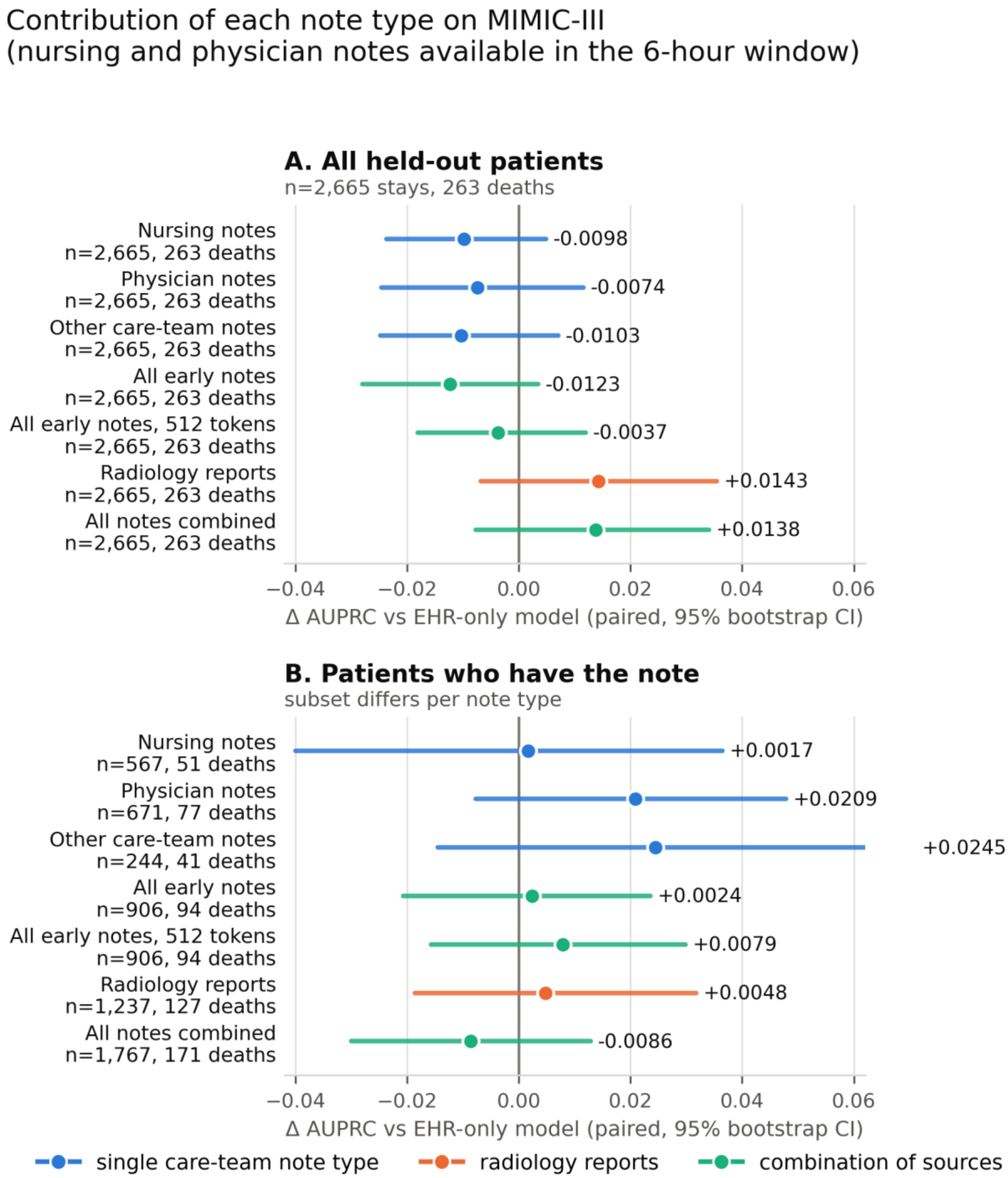

Supplementary Figure S8. Marginal value of each note type in MIMIC-III (Supplementary S1). Paired AUPRC differences against the EHR-only model for seven text definitions — nursing notes, physician notes, other early notes, all early notes, radiology reports, all note types merged, and a 512-token variant of the all-early-notes arm — on the full test set and on the subset carrying the note type in question. No confidence interval excludes zero (0 of 14 comparisons).

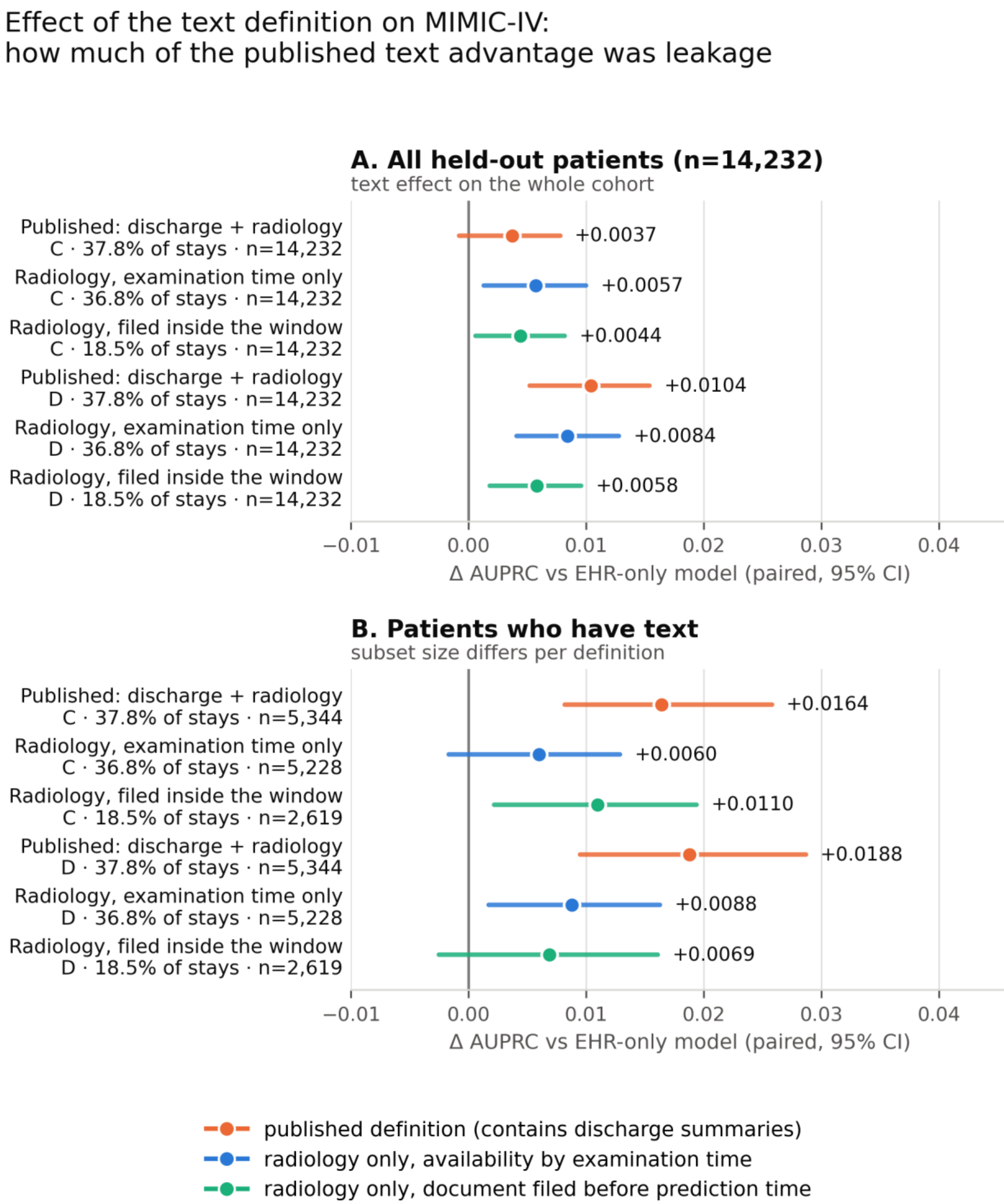

Supplementary Figure S9. Effect of the text-admissibility definition in MIMIC-IV. The paired AUPRC difference against the EHR-only model under the published definition (discharge summaries and radiology reports selected by charting time), under the charting-time-only radiology arm, and under the strict rule adopted here (radiology reports whose charting and filing times both fall inside the six-hour window). The apparent benefit of clinical text falls as the definition is tightened. The three definitions were compared on one fixed held-out cohort of 14,232 stays, which is why the sample differs from the one the main analysis reports; the comparison is between definitions, not between cohorts.

Supplementary S1. Note-type experiment in MIMIC-III

Supplementary analysis S1: do early nursing and physician notes carry signal that radiology reports do not?

Note types written during the prediction window (nursing notes, physician notes, early progress notes) do not exist in MIMIC-IV, but they do exist in MIMIC-III. The full experiment was therefore repeated on MIMIC-III as a supplementary study, to test whether the limited value of text in MIMIC-IV reflects the note inventory of that database rather than the six-hour task.

S1.1 Design

Cohort. MIMIC-III v1.4, restricted to the MetaVision era (DBSOURCE ∈ {metavision, both}): 23,756 ICU stays, reduced to 17,763 by the inclusion criteria of the main study (age 18 or over at ICU admission and the first ICU stay of each patient), with 1,777 deaths within 30 days of ICU admission (10.0%). The MetaVision era is the half of MIMIC-III whose chart item identifiers are the same as in MIMIC-IV, which lets us keep the entire feature pipeline unchanged; the CareVue era (2001–2008) uses a different item vocabulary and was excluded. Split at patient level 70/15/15 (12,434/2,664/2,665 stays; 263 deaths in the held-out set).

Features. The same 1,587-column design matrix as the main study, built with the same aggregation (mean/min/max/last/count within `[intime, intime + 6 h]`), the same missing-value convention, and a z-scaler refitted on the MIMIC-III training split. Of the 1,587 columns, 1,302 (82.0%) could be populated from MIMIC-III. Total ICU length of stay is not a predictor, as in the main study.

Modalities. MIMIC-III has no linked twelve-lead ECG resource, so the ECG arm of the main study cannot be reproduced; the supplementary study compares the EHR-only model A against EHR + text models C, one per text source. Every arm is an ensemble of five seeds, trained with the hyperparameters of the main study.

This arm has been rebuilt under the rules of the main analysis: the outcome is death within 30 days of ICU admission rather than open-ended in-hospital death, the cohort is the first ICU stay of each adult patient rather than every MetaVision stay, total length of stay is not a predictor, and chart observations are admitted only if they had been filed before the six-hour window closed. Both the arm built under the earlier rules and the rebuilt arm are retained in the released result tables, so the two can be compared. One rule could not be carried over: MIMIC-III records no filing time for laboratory results or for radiology notes, so those two sources are admitted on charting time alone, and the count of affected observations is given above.

Text sources. All are restricted to CHARTTIME inside the 6-hour window; for the care-team notes we additionally require STORETIME inside the window, i.e., the document has been filed at prediction time. The same filing-time rule is applied to the structured record: 201,263 of 2,650,050 in-window chart events (7.6%) were filed after the window closed and are dropped, and 136,597 that carry no filing timestamp are kept. It cannot be applied to laboratory results: MIMIC-III's LABEVENTS table has no STORETIME column, so all 519,235 in-window laboratory observations are admitted on charting time alone. MIMIC-III radiology notes carry no filing timestamp either. Discharge summaries cannot leak into the window in MIMIC-III at all: none of the 59,652 summaries carries a `CHARTTIME`.

Arm	Note source	Notes in window	Stays with text	Test stays	Mean characters
C_nursing	`Nursing`, `Nursing/other`	7,360	3,685 (20.8%)	567	2,454
C_physician	`Physician`	8,262	4,346 (24.5%)	671	13,752
C_other	respiratory, general, nutrition, social	1,932	1,600 (9.0%)	244	1,934

	work, rehab, consult, pharmacy, case management				
C_early	all of the above	17,380	5,877 (33.1%)	906	12,124
C_early512	as C_early, 512-token budget instead of 256	17,380	5,877 (33.1%)	906	12,124
C_rad	Radiology	11,889	8,046 (45.3%)	1,237	2,640
C_all	early + radiology	29,028	11,474 (64.6%)	1,767	7,995

The physician arm is the closest analogue of an admission note: these are the MICU/SICU admission and daily assessment notes, and they are by far the longest documents (mean 13,752 characters). We therefore added the 512-token arm: a null result at 256 tokens could otherwise be dismissed as a truncation artifact.

S1.2 Internal results (held-out test set, n=2,665, 263 deaths)

The EHR-only baseline reaches AUROC 0.8713 and AUPRC 0.5133. Each row is the paired difference of the corresponding text model against that baseline, computed on identical patients (1,000-resample bootstrap; DeLong for AUROC).

Arm	Subset	n (events)	AUROC A → C	ΔAUROC (DeLong p)	ΔAUPRC [95% CI]
C_nursing	full test set	2,665 (263)	0.8713 → 0.8725	+0.0013 (p = 0.48)	-0.0098 [−0.0237, 0.0049]
C_physician	full test set	2,665 (263)	0.8713 → 0.8699	-0.0013 (p = 0.56)	-0.0074 [−0.0246, 0.0116]
C_other	full test set	2,665 (263)	0.8713 → 0.8698	-0.0014 (p = 0.45)	-0.0103 [−0.0248, 0.0071]
C_early	full test set	2,665 (263)	0.8713 → 0.8688	-0.0025 (p = 0.26)	-0.0123 [−0.0280, 0.0035]
C_early512	full test set	2,665 (263)	0.8713 → 0.8722	+0.0009 (p = 0.65)	-0.0037 [−0.0181, 0.0120]
C_rad	full test set	2,665 (263)	0.8713 → 0.8770	+0.0058 (p = 0.04)	+0.0143 [−0.0068, 0.0355]
C_all	full test set	2,665 (263)	0.8713 → 0.8734	+0.0021 (p = 0.52)	+0.0138 [−0.0077, 0.0341]
C_nursing	stays with a nursing note	567 (51)	0.8800 → 0.8880	+0.0080 (p = 0.05)	+0.0017 [−0.0400, 0.0365]
C_physician	stays with a physician note	671 (77)	0.8665 → 0.8731	+0.0066 (p = 0.09)	+0.0209 [−0.0077, 0.0479]
C_other	stays with such a note	244 (41)	0.9255 → 0.9254	-0.0001 (p = 0.98)	+0.0245 [−0.0145, 0.0710]
C_early	stays with any early note	906 (94)	0.8750 → 0.8765	+0.0015 (p = 0.71)	+0.0024 [−0.0207, 0.0236]
C_early512	stays with any early note	906 (94)	0.8750 → 0.8759	+0.0009 (p = 0.81)	+0.0079 [−0.0157, 0.0299]
C_rad	stays with a radiology report	1,237 (127)	0.8680 → 0.8725	+0.0046 (p = 0.21)	+0.0048 [−0.0186, 0.0318]
C_all	stays with any note	1,767 (171)	0.8720 → 0.8724	+0.0004 (p = 0.92)	-0.0086 [−0.0300, 0.0129]

No confidence interval in this table excludes zero (0 of 14 comparisons). Supplementary Figure S8 shows the same comparisons graphically: every interval crosses the zero line, and no note type separates from the others.

Three observations.

Discrimination barely changes. No arm moves AUROC by more than 0.0058 on the full test set. One of the fourteen comparisons reaches a nominal p below 0.05: the C_rad arm, ΔAUROC +0.0058, p = 0.039. It is one of fourteen tests, does not survive correction, and its AUPRC interval, [−0.0068, 0.0355], contains zero. The direction of the effect is not consistent either: five of the seven arms have

a negative ΔAUPRC. This is the same answer the MIMIC-IV analysis gives, now obtained with the note types MIMIC-IV does not contain.

Precision-recall effects are small and no interval excludes zero. The largest point estimate on the full test set is +0.0143 AUPRC for the C_rad arm ([−0.0068, 0.0355]); every interval in the table above contains zero. For comparison, the rebuilt MIMIC-IV analysis finds +0.0004 [−0.0036, 0.0044] for radiology text on the full cohort against the primary 30-day outcome**.** In other words the care-team notes behave like the radiology reports: a small positive nudge in the high-specificity region, nothing more.

Adding more note types does not accumulate. Merging all early notes (C_early) or all notes (C_all) performs *worse* than the single best source, presumably because concatenating several long documents and truncating at a fixed token budget dilutes each of them. Raising the budget to 512 tokens (C_early512) does not change the conclusion, so the null result is not an artifact of truncation.

To compare note types on the same patients rather than on their own subsets, we also scored every arm on fixed subsets. In this secondary analysis 4 of 42 arm × subset intervals excluded zero, close to what multiplicity alone would produce (≈2 expected at the 5% level); none of the four corresponded to a significant result in the primary per-arm analysis above, and no note type was consistently ahead of the others. On the patients who have both a nursing and a physician note (n=491, 58 deaths), all arms lie within ΔAUPRC ±0.022 of the EHR-only baseline with intervals spanning zero.

Power. The MIMIC-III held-out set contains 263 deaths against 1,042 30-day events in the MIMIC-IV held-out set, so the bootstrap intervals here are roughly 2.5 times as wide (mean half-width ±0.0099 vs ±0.0036 AUPRC on the full test set). This analysis can therefore exclude an effect larger than about one AUPRC point, but not a genuinely small one; the point estimates, not the significance tests, are what should be compared across the two databases.

S1.3 Conclusion of the supplementary analysis

With nursing and physician notes documented within the first six ICU hours, available for 21% and 24% of stays, the incremental value of clinical text over a structured-EHR baseline remains small and statistically indistinguishable from zero, and no larger than what radiology reports provide. This arm is supportive, hypothesis-generating evidence rather than an independent replication: MIMIC-III and MIMIC-IV originate from the same institution and cover overlapping calendar years, the arm has no ECG branch, and its held-out set is small enough that only effects larger than about one AUPRC point could be excluded. Read within those limits, it is consistent with the conclusion of the main study not being an artifact of MIMIC-IV’s restricted note inventory: across six definitions of the text modality at a six-hour horizon, free-text documentation adds at most a fraction of a point of AUPRC and no discrimination.

S1.4 Reproduction

The supplementary analysis runs in seven stages, in this order: an inventory of the note tables and of their coverage of the six-hour window; construction of the cohort and of the structured feature matrix in the schema of the main analysis; construction of the seven text definitions; training of forty models, the EHR-only arm together with seven text arms at five seeds each; internal evaluation of every arm on the held-out set; a same-patient comparison across arms on fixed subsets; and assembly of the summary tables. The script that performs each stage, and the aggregate tables each one writes, are in the public repository named in the Data Availability Statement.

Model A is trained once and reused across arms (it never reads the text branch); we verified that its predictions are bit-identical in all seven arm-specific evaluations.
